# Supplementary material for: Disclosing the native blueberry rhizosphere community in Portugal—an integrated metagenomic and isolation approach
Source: PeerJ. 2023 Jun 27;11:e15525. doi: 10.7717/peerj.15525 (PMC10312161; doi:10.7717/peerj.15525)
Supplement: Supplemental Information 1 — k: kingdom; p: phylum; c: class; o: order; f: family; g: genus; s:species. [file peerj-11-15525-s001.docx]

**Table S1**. ITS common OTUs between samples, and their taxonomic identification. k: kingdom; p: phylum; c: class; o: order; f: family; g: genus; s:species.

| OTU Number | Taxonomic identification |
| --- | --- |
| OTU_2 | k__Fungi; p__Basidiomycota; c__Agaricomycetes |
| OTU_4 | No blast hit |
| OTU_6 | No blast hit |
| OTU_7 | k__Fungi; p__Basidiomycota; c__Agaricomycetes; o__Sebacinales; f__Serendipitaceae; g__Serendipita |
| OTU_9 | k__Fungi; p__Basidiomycota; c__Agaricomycetes; o__Agaricales; f__Strophariaceae; g__Galerina; s__Galerina_atkinsoniana |
| OTU_15 | k__Fungi; p__Basidiomycota; c__Agaricomycetes; o__Sebacinales; f__Serendipitaceae; g__Serendipita |
| OTU_20 | No blast hit |
| OTU_23 | k__Fungi; p__Basidiomycota; c__Agaricomycetes |
| OTU_27 | k__Fungi; p__Ascomycota; c__Eurotiomycetes; o__Chaetothyriales |
| OTU_28 | No blast hit |
| OTU_42 | k__Fungi; p__Basidiomycota; c__Agaricomycetes; o__Sebacinales; f__Serendipitaceae |
| OTU_48 | No blast hit |
| OTU_56 | k__Fungi; p__Ascomycota; c__Eurotiomycetes; o__Eurotiales; f__Aspergillaceae; g__Aspergillus; s__Aspergillus_niger |
| OTU_64 | k__Fungi; p__Mucoromycota; c__Umbelopsidomycetes; o__Umbelopsidales; f__Umbelopsidaceae; g__Umbelopsis; s__Umbelopsis_vinacea |
| OTU_73 | k__Fungi; p__Ascomycota; c__Sordariomycetes; o__Hypocreales; f__Hypocreaceae; g__Trichoderma; s__Trichoderma_atroviride |
| OTU_94 | k__Fungi; p__Ascomycota; c__Dothideomycetes; o__Pleosporales; f__Didymosphaeriaceae; g__Pseudopithomyces |
| OTU_123 | k__Fungi; p__Ascomycota; c__Eurotiomycetes; o__Chaetothyriales; f__Herpotrichiellaceae; g__Cladophialophora |
| OTU_124 | k__Fungi; p__Ascomycota; c__Sordariomycetes; o__Hypocreales; f__Hypocreaceae; g__Trichoderma; s__Trichoderma_harzianum |
| OTU_139 | k__Fungi; p__Ascomycota; c__Sordariomycetes; o__Hypocreales; f__Hypocreaceae; g__Trichoderma; s__Trichoderma_harzianum |
| OTU_1210 | k__Fungi; p__Basidiomycota; c__Agaricomycetes; o__Sebacinales; f__Serendipitaceae; g__Serendipita |
