# Supplementary material for: Disclosing the native blueberry rhizosphere community in Portugal—an integrated metagenomic and isolation approach
Source: PeerJ. 2023 Jun 27;11:e15525. doi: 10.7717/peerj.15525 (PMC10312161; doi:10.7717/peerj.15525)
Supplement: Supplemental Information 2 — k: kingdom; p: phylum; c: class; o: order; f: family; g: genus; s:species. [file peerj-11-15525-s002.docx]

**Table S2**. 16s common OTUs between samples, and their taxonomic identification. k: kingdom; p: phylum; c: class; o: order; f: family; g: genus; s:species.

| OTU Number | Taxonomic identification |
| --- | --- |
| OTU_1 | k__Bacteria; p__Proteobacteria; c__Alphaproteobacteria; o__Rhizobiales; f__Xanthobacteraceae; g__Bradyrhizobium; s__Bradyrhizobium_elkanii |
| OTU_2 | k__Bacteria; p__Spirochaetota; c__Brevinematia; o__Brevinematales; f__Brevinemataceae; g__Brevinema; s__ |
| OTU_3 | k__Bacteria; p__Proteobacteria; c__Alphaproteobacteria; o__Rhizobiales; f__Xanthobacteraceae |
| OTU_4 | k__Bacteria; p__Acidobacteriota; c__Acidobacteriae; o__Acidobacteriales; f__; g__; s__ |
| OTU_5 | k__Bacteria; p__Actinobacteriota; c__Actinobacteria; o__Frankiales; f__Acidothermaceae; g__Acidothermus; s__ |
| OTU_6 | k__Bacteria; p__Actinobacteriota; c__Actinobacteria; o__0319-7L14; f__0319-7L14; g__0319-7L14; s__ |
| OTU_7 | k__Bacteria; p__Cyanobacteria; c__Cyanobacteriia; o__Chloroplast; f__Chloroplast; g__Chloroplast |
| OTU_8 | k__Bacteria; p__Acidobacteriota; c__Acidobacteriae; o__Acidobacteriales; f__; g__; s__ |
| OTU_9 | k__Bacteria; p__Proteobacteria; c__Alphaproteobacteria; o__Rhizobiales; f__Rhizobiaceae; g__Allorhizobium-Neorhizobium-Pararhizobium-Rhizobium |
| OTU_10 | k__Bacteria; p__Proteobacteria; c__Alphaproteobacteria; o__Rhizobiales; f__Rhizobiaceae; g__Allorhizobium-Neorhizobium-Pararhizobium-Rhizobium |
| OTU_11 | k__Bacteria; p__Actinobacteriota; c__Acidimicrobiia |
| OTU_12 | k__Bacteria; p__Actinobacteriota; c__Actinobacteria; o__Frankiales; f__Acidothermaceae; g__Acidothermus; s__ |
| OTU_13 | k__Bacteria; p__Chloroflexi; c__AD3; o__AD3; f__AD3; g__AD3; s__ |
| OTU_14 | k__Bacteria; p__Actinobacteriota; c__Actinobacteria; o__Pseudonocardiales; f__Pseudonocardiaceae; g__Crossiella; s__ |
| OTU_15 | k__Bacteria; p__Acidobacteriota; c__Acidobacteriae; o__Subgroup_2; f__Subgroup_2; g__Subgroup_2; s__ |
| OTU_16 | k__Bacteria; p__Gemmatimonadota; c__Gemmatimonadetes; o__Gemmatimonadales; f__Gemmatimonadaceae; g__; s__ |
| OTU_17 | k__Bacteria; p__Proteobacteria; c__Alphaproteobacteria; o__Sphingomonadales; f__Sphingomonadaceae; g__Sphingomonas; s__ |
| OTU_18 | k__Bacteria; p__Acidobacteriota; c__Acidobacteriae; o__Acidobacteriales; f__; g__; s__ |
| OTU_19 | k__Bacteria; p__Gemmatimonadota; c__Gemmatimonadetes; o__Gemmatimonadales; f__Gemmatimonadaceae; g__; s__ |
| OTU_20 | k__Bacteria; p__Proteobacteria; c__Alphaproteobacteria; o__Reyranellales; f__Reyranellaceae; g__Reyranella; s__ |
| OTU_21 | k__Bacteria; p__Myxococcota; c__Polyangia; o__Haliangiales; f__Haliangiaceae; g__Haliangium; s__ |
| OTU_22 | k__Bacteria; p__Acidobacteriota; c__Acidobacteriae; o__Bryobacterales; f__Bryobacteraceae; g__Bryobacter; s__ |
| OTU_23 | k__Bacteria; p__Proteobacteria; c__Alphaproteobacteria; o__Acetobacterales; f__Acetobacteraceae; g__; s__ |
| OTU_24 | k__Bacteria; p__Proteobacteria; c__Alphaproteobacteria; o__Micropepsales; f__Micropepsaceae; g__; s__ |
| OTU_25 | k__Bacteria; p__Proteobacteria; c__Alphaproteobacteria; o__; f__; g__; s__ |
| OTU_26 | k__Bacteria; p__Proteobacteria; c__Alphaproteobacteria; o__Rhizobiales; f__Hyphomicrobiaceae; g__Pedomicrobium; s__ |
| OTU_27 | k__Bacteria; p__Proteobacteria; c__Gammaproteobacteria; o__Gammaproteobacteria_Incertae_Sedis; f__Unknown_Family; g__Acidibacter; s__ |
| OTU_28 | k__Bacteria; p__Acidobacteriota; c__Acidobacteriae; o__Subgroup_2; f__Subgroup_2; g__Subgroup_2; s__ |
| OTU_29 | k__Bacteria; p__Proteobacteria; c__Alphaproteobacteria; o__Rhizobiales; f__Hyphomicrobiaceae; g__Hyphomicrobium; s__Hyphomicrobium_facile |
| OTU_30 | k__Bacteria; p__Proteobacteria; c__Alphaproteobacteria; o__; f__; g__ |
| OTU_31 | k__Bacteria; p__Proteobacteria; c__Alphaproteobacteria; o__Rhizobiales; f__Xanthobacteraceae |
| OTU_32 | k__Bacteria; p__Acidobacteriota; c__Acidobacteriae; o__Bryobacterales; f__Bryobacteraceae; g__Bryobacter; s__ |
| OTU_33 | k__Bacteria; p__Chloroflexi; c__AD3; o__AD3; f__AD3; g__AD3; s__ |
| OTU_36 | k__Bacteria; p__Acidobacteriota; c__Acidobacteriae; o__Acidobacteriales; f__; g__; s__ |
| OTU_37 | k__Bacteria; p__Actinobacteriota; c__Actinobacteria; o__Pseudonocardiales; f__Pseudonocardiaceae; g__Amycolatopsis; s__Amycolatopsis_mediterranei |
| OTU_38 | k__Bacteria; p__Acidobacteriota; c__Acidobacteriae; o__Bryobacterales; f__Bryobacteraceae; g__Bryobacter; s__ |
| OTU_39 | k__Bacteria; p__Actinobacteriota; c__Actinobacteria; o__Frankiales; f__Acidothermaceae; g__Acidothermus |
| OTU_40 | k__Bacteria; p__Actinobacteriota; c__Actinobacteria; o__Micrococcales; f__Micrococcaceae; g__Pseudarthrobacter; s__Arthrobacter_sp. |
| OTU_41 | k__Archaea; p__Crenarchaeota; c__Nitrososphaeria; o__Nitrosotaleales; f__Nitrosotaleaceae; g__Nitrosotaleaceae; s__ |
| OTU_42 | k__Bacteria; p__Proteobacteria; c__Alphaproteobacteria; o__Elsterales; f__; g__; s__ |
| OTU_43 | k__Bacteria; p__Firmicutes; c__Bacilli; o__Bacillales; f__Bacillaceae; g__Bacillus; s__Bacillus_thuringiensis |
| OTU_44 | k__Bacteria; p__Proteobacteria; c__Alphaproteobacteria; o__Rhizobiales; f__Labraceae; g__Labrys |
| OTU_45 | k__Archaea; p__Crenarchaeota; c__Nitrososphaeria; o__Nitrosotaleales; f__Nitrosotaleaceae; g__Candidatus_Nitrosotalea; s__ |
| OTU_46 | k__Bacteria; p__Methylomirabilota; c__Methylomirabilia; o__Rokubacteriales; f__Rokubacteriales; g__Rokubacteriales; s__ |
| OTU_47 | k__Bacteria; p__Actinobacteriota; c__Actinobacteria; o__Frankiales; f__Acidothermaceae; g__Acidothermus |
| OTU_48 | k__Bacteria; p__Actinobacteriota; c__Actinobacteria; o__Corynebacteriales; f__Mycobacteriaceae; g__Mycobacterium |
| OTU_49 | k__Bacteria; p__Actinobacteriota; c__Actinobacteria; o__Propionibacteriales; f__Propionibacteriaceae; g__Cutibacterium; s__Cutibacterium_acnes |
| OTU_51 | k__Bacteria; p__Actinobacteriota; c__Acidimicrobiia; o__IMCC26256; f__IMCC26256; g__IMCC26256; s__ |
| OTU_52 | k__Archaea; p__Crenarchaeota; c__Nitrososphaeria; o__Nitrososphaerales; f__Nitrososphaeraceae; g__Nitrososphaeraceae; s__ |
| OTU_53 | k__Bacteria; p__Acidobacteriota; c__Acidobacteriae; o__Acidobacteriales; f__; g__; s__ |
| OTU_54 | k__Bacteria; p__Proteobacteria; c__Alphaproteobacteria; o__Dongiales; f__Dongiaceae; g__Dongia |
| OTU_56 | k__Bacteria; p__Acidobacteriota; c__Acidobacteriae; o__Subgroup_2; f__Subgroup_2; g__Subgroup_2; s__ |
| OTU_57 | k__Bacteria; p__Proteobacteria; c__Gammaproteobacteria; o__Gammaproteobacteria_Incertae_Sedis; f__Unknown_Family; g__Acidibacter; s__ |
| OTU_58 | k__Bacteria; p__Chloroflexi; c__AD3; o__AD3; f__AD3; g__AD3; s__ |
| OTU_59 | k__Bacteria; p__Actinobacteriota; c__Thermoleophilia; o__Gaiellales; f__; g__; s__ |
| OTU_60 | k__Bacteria; p__Proteobacteria; c__Alphaproteobacteria; o__; f__; g__; s__ |
| OTU_62 | k__Bacteria; p__Acidobacteriota; c__Acidobacteriae; o__Subgroup_2; f__Subgroup_2; g__Subgroup_2; s__ |
| OTU_63 | k__Bacteria; p__WPS-2; c__WPS-2; o__WPS-2; f__WPS-2; g__WPS-2; s__ |
| OTU_64 | k__Archaea; p__Thermoplasmatota; c__Thermoplasmata; o__Methanomassiliicoccales; f__; g__; s__ |
| OTU_65 | k__Bacteria; p__Proteobacteria; c__Alphaproteobacteria; o__Rhizobiales; f__Beijerinckiaceae; g__Roseiarcus; s__ |
| OTU_66 | k__Bacteria; p__Bacteroidota; c__Bacteroidia; o__Chitinophagales; f__Chitinophagaceae; g__Puia; s__ |
| OTU_68 | k__Bacteria; p__Acidobacteriota; c__Acidobacteriae; o__Subgroup_2; f__Subgroup_2; g__Subgroup_2; s__ |
| OTU_69 | k__Bacteria; p__Acidobacteriota; c__Acidobacteriae; o__Acidobacteriales; f__Acidobacteriaceae_(Subgroup_1) |
| OTU_71 | k__Bacteria; p__Proteobacteria; c__Alphaproteobacteria; o__Rhizobiales; f__Xanthobacteraceae; g__; s__ |
| OTU_72 | k__Bacteria; p__Actinobacteriota; c__Actinobacteria; o__Streptomycetales; f__Streptomycetaceae; g__Streptomyces |
| OTU_73 | k__Bacteria; p__Proteobacteria; c__Gammaproteobacteria; o__WD260; f__WD260; g__WD260; s__ |
| OTU_74 | k__Bacteria; p__Proteobacteria; c__Alphaproteobacteria; o__Rhizobiales; f__Devosiaceae; g__Devosia; s__ |
| OTU_75 | k__Bacteria; p__Acidobacteriota; c__Thermoanaerobaculia; o__Thermoanaerobaculales; f__Thermoanaerobaculaceae; g__Subgroup_10 |
| OTU_76 | k__Bacteria; p__Chloroflexi; c__AD3; o__AD3; f__AD3; g__AD3; s__ |
| OTU_77 | k__Bacteria; p__Proteobacteria; c__Gammaproteobacteria; o__Burkholderiales; f__Nitrosomonadaceae; g__IS-44; s__ |
| OTU_78 | k__Bacteria; p__Actinobacteriota; c__Actinobacteria; o__Streptomycetales; f__Streptomycetaceae; g__Streptomyces |
| OTU_79 | k__Bacteria; p__Proteobacteria; c__Alphaproteobacteria; o__Rhizobiales; f__KF-JG30-B3; g__KF-JG30-B3; s__ |
| OTU_80 | k__Bacteria; p__Actinobacteriota; c__Actinobacteria; o__Pseudonocardiales; f__Pseudonocardiaceae; g__Kutzneria; s__Kutzneria_albida |
| OTU_82 | k__Bacteria; p__Proteobacteria; c__Alphaproteobacteria; o__Rhizobiales; f__Rhizobiaceae; g__Mesorhizobium; s__Mesorhizobium_huakuii |
| OTU_84 | k__Bacteria; p__Actinobacteriota; c__Actinobacteria; o__Streptosporangiales; f__Streptosporangiaceae; g__Streptosporangium |
| OTU_85 | k__Bacteria; p__Actinobacteriota; c__Actinobacteria; o__Frankiales; f__Acidothermaceae; g__Acidothermus; s__Actinomycetales_bacterium |
| OTU_86 | k__Bacteria; p__Proteobacteria; c__Alphaproteobacteria; o__Rhizobiales; f__Xanthobacteraceae; g__; s__ |
| OTU_87 | k__Bacteria; p__Actinobacteriota; c__Actinobacteria; o__Corynebacteriales; f__Mycobacteriaceae; g__Mycobacterium |
| OTU_88 | k__Bacteria; p__Acidobacteriota; c__Acidobacteriae; o__Acidobacteriales; f__; g__; s__ |
| OTU_89 | k__Bacteria; p__Acidobacteriota; c__Acidobacteriae; o__Solibacterales; f__Solibacteraceae; g__Candidatus_Solibacter; s__ |
| OTU_90 | k__Bacteria; p__Acidobacteriota; c__Acidobacteriae; o__Bryobacterales; f__Bryobacteraceae; g__Bryobacter; s__ |
| OTU_91 | k__Bacteria; p__Nitrospirota; c__Nitrospiria; o__Nitrospirales; f__Nitrospiraceae; g__Nitrospira |
| OTU_92 | k__Bacteria; p__Actinobacteriota; c__Thermoleophilia; o__Gaiellales; f__; g__; s__bacterium_Ellin6515 |
| OTU_93 | k__Bacteria; p__Firmicutes; c__Bacilli; o__Bacillales; f__Bacillaceae; g__Bacillus |
| OTU_94 | k__Bacteria; p__Actinobacteriota; c__Thermoleophilia; o__Gaiellales; f__; g__; s__bacterium_Ellin6517 |
| OTU_95 | k__Bacteria; p__Acidobacteriota; c__Acidobacteriae; o__Solibacterales; f__Solibacteraceae; g__Candidatus_Solibacter; s__ |
| OTU_97 | k__Bacteria; p__Gemmatimonadota; c__Gemmatimonadetes; o__Gemmatimonadales; f__Gemmatimonadaceae; g__; s__ |
| OTU_99 | k__Bacteria; p__Actinobacteriota; c__MB-A2-108; o__MB-A2-108; f__MB-A2-108; g__MB-A2-108; s__ |
| OTU_100 | k__Bacteria; p__Actinobacteriota; c__MB-A2-108; o__MB-A2-108; f__MB-A2-108; g__MB-A2-108; s__ |
| OTU_101 | k__Bacteria; p__Acidobacteriota; c__Acidobacteriae; o__Bryobacterales; f__Bryobacteraceae; g__Bryobacter; s__ |
| OTU_102 | k__Bacteria; p__Actinobacteriota; c__Acidimicrobiia; o__IMCC26256; f__IMCC26256; g__IMCC26256; s__ |
| OTU_103 | k__Bacteria; p__Actinobacteriota; c__Actinobacteria; o__Streptosporangiales; f__Thermomonosporaceae; g__Actinoallomurus |
| OTU_104 | k__Bacteria; p__Methylomirabilota; c__Methylomirabilia; o__Rokubacteriales; f__Rokubacteriales; g__Rokubacteriales; s__ |
| OTU_105 | k__Bacteria; p__Actinobacteriota; c__Actinobacteria; o__Micromonosporales; f__Micromonosporaceae; g__Actinocatenispora |
| OTU_106 | k__Bacteria; p__Proteobacteria; c__Gammaproteobacteria; o__Burkholderiales; f__Nitrosomonadaceae; g__MND1; s__ |
| OTU_107 | k__Bacteria; p__Acidobacteriota; c__Acidobacteriae; o__Subgroup_2; f__Subgroup_2; g__Subgroup_2; s__ |
| OTU_108 | k__Bacteria; p__Cyanobacteria; c__Cyanobacteriia; o__Chloroplast; f__Chloroplast; g__Chloroplast |
| OTU_109 | k__Bacteria; p__Gemmatimonadota; c__Gemmatimonadetes; o__Gemmatimonadales; f__Gemmatimonadaceae |
| OTU_111 | k__Bacteria; p__Proteobacteria; c__Alphaproteobacteria; o__Elsterales; f__; g__; s__ |
| OTU_112 | k__Bacteria; p__Actinobacteriota; c__Acidimicrobiia; o__IMCC26256; f__IMCC26256; g__IMCC26256; s__ |
| OTU_113 | k__Bacteria; p__Proteobacteria; c__Gammaproteobacteria; o__Burkholderiales; f__Nitrosomonadaceae; g__GOUTA6; s__ |
| OTU_114 | k__Bacteria; p__Acidobacteriota; c__Acidobacteriae; o__Solibacterales; f__Solibacteraceae; g__Candidatus_Solibacter; s__ |
| OTU_116 | k__Bacteria; p__Proteobacteria; c__Alphaproteobacteria; o__Rhizobiales; f__Beijerinckiaceae; g__Methylobacterium-Methylorubrum |
| OTU_118 | k__Bacteria; p__Acidobacteriota; c__Acidobacteriae; o__Acidobacteriales |
| OTU_119 | k__Bacteria; p__Proteobacteria; c__Alphaproteobacteria; o__Elsterales; f__; g__; s__ |
| OTU_120 | k__Bacteria; p__Proteobacteria; c__Gammaproteobacteria; o__Burkholderiales; f__Burkholderiaceae; g__Burkholderia-Caballeronia-Paraburkholderia; s__Paraburkholderia_terrae |
| OTU_121 | k__Bacteria; p__Proteobacteria; c__Gammaproteobacteria; o__Burkholderiales; f__Burkholderiaceae; g__Burkholderia-Caballeronia-Paraburkholderia; s__Paraburkholderia_caledonica |
| OTU_122 | k__Bacteria; p__Proteobacteria; c__Alphaproteobacteria; o__Sphingomonadales; f__Sphingomonadaceae; g__Sphingomonas |
| OTU_123 | k__Bacteria; p__Acidobacteriota; c__Acidobacteriae; o__Solibacterales; f__Solibacteraceae; g__Candidatus_Solibacter; s__ |
| OTU_124 | k__Bacteria; p__Actinobacteriota; c__Actinobacteria; o__Micromonosporales; f__Micromonosporaceae |
| OTU_125 | k__Bacteria; p__Actinobacteriota; c__Acidimicrobiia; o__IMCC26256; f__IMCC26256; g__IMCC26256; s__ |
| OTU_126 | k__Bacteria; p__Proteobacteria; c__Gammaproteobacteria; o__Burkholderiales; f__Sutterellaceae; g__; s__ |
| OTU_127 | k__Bacteria; p__Proteobacteria; c__Gammaproteobacteria; o__Xanthomonadales; f__Rhodanobacteraceae |
| OTU_128 | k__Bacteria; p__Myxococcota; c__Myxococcia; o__Myxococcales; f__Anaeromyxobacteraceae; g__Anaeromyxobacter; s__ |
| OTU_129 | k__Bacteria; p__Actinobacteriota; c__Thermoleophilia; o__Solirubrobacterales; f__67-14; g__67-14; s__ |
| OTU_130 | k__Bacteria; p__Acidobacteriota; c__Acidobacteriae; o__Acidobacteriales; f__Acidobacteriaceae_(Subgroup_1); g__Acidipila; s__ |
| OTU_131 | k__Bacteria; p__Actinobacteriota; c__Thermoleophilia; o__Gaiellales |
| OTU_132 | k__Bacteria; p__Proteobacteria; c__Alphaproteobacteria; o__Rhizobiales; f__Xanthobacteraceae; g__Pseudolabrys; s__ |
| OTU_133 | k__Bacteria; p__Firmicutes; c__Bacilli; o__Bacillales; f__Bacillaceae; g__Bacillus |
| OTU_134 | k__Bacteria; p__Proteobacteria; c__Alphaproteobacteria; o__Caulobacterales; f__Caulobacteraceae; g__; s__ |
| OTU_135 | k__Bacteria; p__Acidobacteriota; c__Acidobacteriae; o__Acidobacteriales; f__; g__; s__ |
| OTU_138 | k__Bacteria; p__Acidobacteriota; c__Acidobacteriae; o__Acidobacteriales; f__Acidobacteriaceae_(Subgroup_1); g__Occallatibacter; s__ |
| OTU_139 | k__Bacteria; p__Actinobacteriota; c__Actinobacteria; o__Propionibacteriales; f__Nocardioidaceae; g__Nocardioides; s__ |
| OTU_140 | k__Archaea; p__Crenarchaeota; c__Nitrososphaeria; o__Group_1.1c; f__Group_1.1c; g__Group_1.1c; s__ |
| OTU_141 | k__Bacteria; p__Acidobacteriota; c__Acidobacteriae; o__Acidobacteriales; f__; g__; s__ |
| OTU_143 | k__Bacteria; p__Acidobacteriota; c__Acidobacteriae; o__Bryobacterales; f__Bryobacteraceae; g__Bryobacter; s__ |
| OTU_144 | k__Bacteria; p__Actinobacteriota; c__Acidimicrobiia; o__; f__; g__; s__ |
| OTU_146 | k__Bacteria; p__Actinobacteriota; c__Thermoleophilia; o__Gaiellales; f__Gaiellaceae; g__Gaiella; s__ |
| OTU_147 | k__Bacteria; p__Proteobacteria; c__Gammaproteobacteria; o__Burkholderiales; f__Nitrosomonadaceae; g__MND1; s__ |
| OTU_148 | k__Bacteria; p__Actinobacteriota; c__Acidimicrobiia; o__IMCC26256; f__IMCC26256; g__IMCC26256; s__ |
| OTU_149 | k__Bacteria; p__Proteobacteria; c__Alphaproteobacteria; o__Elsterales; f__; g__; s__ |
| OTU_150 | k__Bacteria; p__Acidobacteriota; c__Acidobacteriae; o__Solibacterales; f__Solibacteraceae; g__Candidatus_Solibacter; s__ |
| OTU_151 | k__Bacteria; p__Actinobacteriota; c__Thermoleophilia; o__Gaiellales |
| OTU_152 | k__Bacteria; p__Proteobacteria; c__Gammaproteobacteria; o__Burkholderiales; f__Burkholderiaceae; g__Burkholderia-Caballeronia-Paraburkholderia |
| OTU_154 | k__Bacteria; p__Chloroflexi; c__AD3; o__AD3; f__AD3; g__AD3; s__ |
| OTU_155 | k__Bacteria; p__Chloroflexi; c__TK10; o__TK10; f__TK10; g__TK10 |
| OTU_156 | k__Bacteria; p__Verrucomicrobiota; c__Verrucomicrobiae; o__Pedosphaerales; f__Pedosphaeraceae; g__ADurb.Bin063-1; s__ |
| OTU_157 | k__Bacteria; p__Acidobacteriota; c__Acidobacteriae; o__Acidobacteriales; f__Koribacteraceae; g__Candidatus_Koribacter; s__ |
| OTU_158 | k__Bacteria; p__Nitrospirota; c__Nitrospiria; o__Nitrospirales; f__Nitrospiraceae; g__Nitrospira; s__ |
| OTU_159 | k__Bacteria; p__Gemmatimonadota; c__Gemmatimonadetes; o__Gemmatimonadales; f__Gemmatimonadaceae; g__; s__ |
| OTU_160 | k__Bacteria; p__Bacteroidota; c__Bacteroidia; o__Chitinophagales; f__Chitinophagaceae; g__Puia; s__ |
| OTU_161 | k__Bacteria; p__Proteobacteria; c__Alphaproteobacteria; o__Elsterales; f__; g__; s__ |
| OTU_163 | k__Bacteria; p__Actinobacteriota; c__Actinobacteria; o__Frankiales; f__Acidothermaceae; g__Acidothermus; s__ |
| OTU_164 | k__Bacteria; p__Proteobacteria; c__Alphaproteobacteria; o__Micropepsales; f__Micropepsaceae; g__; s__ |
| OTU_165 | k__Bacteria; p__Chloroflexi; c__Ktedonobacteria; o__Ktedonobacterales; f__JG30-KF-AS9; g__JG30-KF-AS9; s__Chloroflexi_bacterium |
| OTU_166 | k__Bacteria; p__Chloroflexi; c__KD4-96; o__KD4-96; f__KD4-96; g__KD4-96; s__ |
| OTU_167 | k__Bacteria; p__Actinobacteriota; c__Actinobacteria; o__Catenulisporales; f__Catenulisporaceae; g__Catenulispora |
| OTU_168 | k__Bacteria; p__Proteobacteria; c__Gammaproteobacteria; o__Burkholderiales; f__Nitrosomonadaceae; g__Ellin6067; s__ |
| OTU_169 | k__Bacteria; p__Gemmatimonadota; c__Gemmatimonadetes; o__Gemmatimonadales; f__Gemmatimonadaceae; g__; s__ |
| OTU_170 | k__Bacteria; p__Acidobacteriota; c__Vicinamibacteria; o__Vicinamibacterales; f__; g__; s__ |
| OTU_171 | k__Bacteria; p__Myxococcota; c__Polyangia; o__Polyangiales; f__Phaselicystidaceae; g__Phaselicystis; s__ |
| OTU_172 | k__Bacteria; p__Proteobacteria; c__Gammaproteobacteria; o__Burkholderiales; f__Nitrosomonadaceae; g__mle1-7; s__ |
| OTU_174 | k__Bacteria; p__Acidobacteriota; c__Thermoanaerobaculia; o__Thermoanaerobaculales; f__Thermoanaerobaculaceae; g__Subgroup_10 |
| OTU_175 | k__Bacteria; p__Acidobacteriota; c__Acidobacteriae; o__Solibacterales; f__Solibacteraceae; g__Candidatus_Solibacter |
| OTU_176 | k__Bacteria; p__Actinobacteriota; c__Actinobacteria; o__Propionibacteriales; f__Nocardioidaceae; g__Marmoricola; s__ |
| OTU_177 | k__Bacteria; p__Proteobacteria; c__Alphaproteobacteria; o__Rhizobiales; f__Methyloligellaceae; g__; s__ |
| OTU_178 | k__Bacteria; p__Acidobacteriota; c__Acidobacteriae; o__Acidobacteriales; f__Acidobacteriaceae_(Subgroup_1); g__Occallatibacter; s__ |
| OTU_179 | k__Bacteria; p__Proteobacteria; c__Alphaproteobacteria; o__Dongiales; f__Dongiaceae; g__Dongia; s__ |
| OTU_180 | k__Bacteria; p__Chloroflexi; c__AD3; o__AD3; f__AD3; g__AD3; s__ |
| OTU_181 | k__Bacteria; p__Proteobacteria; c__Alphaproteobacteria; o__Acetobacterales; f__Acetobacteraceae; g__; s__ |
| OTU_182 | k__Bacteria; p__Proteobacteria; c__Alphaproteobacteria; o__Rhizobiales; f__Rhizobiales_Incertae_Sedis; g__Bauldia; s__ |
| OTU_183 | k__Bacteria; p__Chloroflexi; c__Ktedonobacteria; o__Ktedonobacterales; f__Ktedonobacteraceae; g__1959-1; s__ |
| OTU_184 | k__Bacteria; p__Actinobacteriota; c__Acidimicrobiia; o__Microtrichales; f__; g__; s__ |
| OTU_185 | k__Bacteria; p__Actinobacteriota; c__Thermoleophilia; o__Gaiellales; f__; g__ |
| OTU_186 | k__Bacteria; p__Actinobacteriota; c__Acidimicrobiia; o__IMCC26256; f__IMCC26256; g__IMCC26256; s__ |
| OTU_187 | k__Bacteria; p__Proteobacteria; c__Gammaproteobacteria; o__Burkholderiales; f__A21b; g__A21b; s__ |
| OTU_188 | k__Bacteria; p__Verrucomicrobiota; c__Verrucomicrobiae; o__Chthoniobacterales; f__Chthoniobacteraceae; g__Candidatus_Udaeobacter; s__ |
| OTU_190 | k__Bacteria; p__Acidobacteriota; c__Acidobacteriae; o__Acidobacteriales; f__Acidobacteriaceae_(Subgroup_1); g__Edaphobacter; s__ |
| OTU_191 | k__Bacteria; p__Acidobacteriota; c__Acidobacteriae; o__Subgroup_2; f__Subgroup_2; g__Subgroup_2; s__ |
| OTU_192 | k__Bacteria; p__Actinobacteriota; c__MB-A2-108; o__MB-A2-108; f__MB-A2-108; g__MB-A2-108; s__ |
| OTU_193 | k__Bacteria; p__Proteobacteria; c__Gammaproteobacteria; o__Burkholderiales; f__Nitrosomonadaceae; g__Nitrosospira |
| OTU_194 | k__Bacteria; p__Acidobacteriota; c__Vicinamibacteria; o__Vicinamibacterales; f__; g__; s__ |
| OTU_195 | k__Bacteria; p__Actinobacteriota; c__Thermoleophilia; o__Gaiellales; f__Gaiellaceae; g__Gaiella; s__ |
| OTU_197 | k__Bacteria; p__Myxococcota; c__Polyangia; o__Haliangiales; f__Haliangiaceae; g__Haliangium; s__ |
| OTU_198 | k__Bacteria; p__Proteobacteria; c__Alphaproteobacteria; o__Sphingomonadales; f__Sphingomonadaceae; g__Sphingomonas; s__Sphingomonas_faeni |
| OTU_199 | k__Bacteria; p__Actinobacteriota; c__Actinobacteria; o__Frankiales; f__Nakamurellaceae; g__Nakamurella; s__ |
| OTU_201 | k__Bacteria; p__Myxococcota; c__Polyangia; o__Polyangiales; f__Polyangiaceae; g__Pajaroellobacter; s__ |
| OTU_204 | k__Bacteria; p__Proteobacteria; c__Alphaproteobacteria; o__Rhizobiales; f__Devosiaceae; g__; s__ |
| OTU_205 | k__Bacteria; p__Actinobacteriota; c__Actinobacteria; o__Micrococcales; f__Intrasporangiaceae |
| OTU_206 | k__Bacteria; p__Actinobacteriota; c__Acidimicrobiia; o__; f__; g__; s__ |
| OTU_207 | k__Bacteria; p__Methylomirabilota; c__Methylomirabilia; o__Rokubacteriales; f__Rokubacteriales; g__Rokubacteriales; s__ |
| OTU_210 | k__Bacteria; p__Acidobacteriota; c__Acidobacteriae; o__Acidobacteriales; f__Acidobacteriaceae_(Subgroup_1); g__Acidipila; s__ |
| OTU_212 | k__Bacteria; p__Actinobacteriota; c__Acidimicrobiia; o__Microtrichales |
| OTU_216 | k__Bacteria; p__Proteobacteria; c__Alphaproteobacteria; o__Rhizobiales; f__Rhizobiales_Incertae_Sedis; g__; s__ |
| OTU_217 | k__Bacteria; p__Actinobacteriota; c__Acidimicrobiia; o__Microtrichales; f__Iamiaceae; g__Iamia; s__ |
| OTU_218 | k__Bacteria; p__Myxococcota; c__Myxococcia; o__Myxococcales; f__Anaeromyxobacteraceae; g__Anaeromyxobacter; s__ |
| OTU_219 | k__Bacteria; p__Actinobacteriota; c__Actinobacteria; o__Frankiales; f__Frankiaceae; g__Jatrophihabitans; s__ |
| OTU_220 | k__Bacteria; p__Proteobacteria; c__Alphaproteobacteria; o__Elsterales; f__; g__; s__ |
| OTU_221 | k__Bacteria; p__Acidobacteriota; c__Holophagae; o__Subgroup_7; f__Subgroup_7; g__Subgroup_7; s__ |
| OTU_222 | k__Archaea; p__Crenarchaeota; c__Nitrososphaeria; o__Nitrososphaerales; f__Nitrososphaeraceae; g__Nitrososphaeraceae; s__ |
| OTU_223 | k__Bacteria; p__Actinobacteriota; c__Thermoleophilia; o__Solirubrobacterales; f__67-14; g__67-14; s__ |
| OTU_224 | k__Bacteria; p__Proteobacteria; c__Alphaproteobacteria; o__Elsterales; f__URHD0088; g__URHD0088; s__ |
| OTU_225 | k__Bacteria; p__Actinobacteriota; c__Actinobacteria; o__Frankiales; f__; g__; s__ |
| OTU_226 | k__Bacteria; p__Myxococcota; c__Polyangia; o__Polyangiales; f__Polyangiaceae |
| OTU_227 | k__Bacteria; p__Desulfobacterota; c__; o__; f__; g__; s__ |
| OTU_228 | k__Bacteria; p__Actinobacteriota; c__Actinobacteria; o__Pseudonocardiales; f__Pseudonocardiaceae; g__Pseudonocardia; s__ |
| OTU_229 | k__Bacteria; p__Actinobacteriota; c__Thermoleophilia; o__Solirubrobacterales; f__Solirubrobacteraceae; g__Conexibacter; s__ |
| OTU_230 | k__Bacteria; p__Acidobacteriota; c__Acidobacteriae; o__Acidobacteriales; f__; g__; s__ |
| OTU_231 | k__Bacteria; p__Actinobacteriota; c__Actinobacteria; o__Frankiales; f__Geodermatophilaceae |
| OTU_232 | k__Bacteria; p__Gemmatimonadota; c__Gemmatimonadetes; o__Gemmatimonadales; f__Gemmatimonadaceae; g__; s__ |
| OTU_233 | k__Bacteria; p__Proteobacteria; c__Gammaproteobacteria; o__Xanthomonadales; f__Rhodanobacteraceae; g__Dokdonella; s__ |
| OTU_234 | k__Bacteria; p__Proteobacteria; c__Gammaproteobacteria; o__Burkholderiales; f__Nitrosomonadaceae; g__MND1; s__ |
| OTU_235 | k__Bacteria; p__Actinobacteriota; c__Acidimicrobiia; o__Acidimicrobiales; f__Acidimicrobiaceae; g__; s__ |
| OTU_236 | k__Bacteria; p__Proteobacteria; c__Gammaproteobacteria; o__Burkholderiales; f__Burkholderiaceae; g__Burkholderia-Caballeronia-Paraburkholderia |
| OTU_238 | k__Bacteria; p__Actinobacteriota; c__Thermoleophilia; o__Gaiellales; f__; g__; s__ |
| OTU_240 | k__Bacteria; p__Actinobacteriota; c__Thermoleophilia; o__Gaiellales; f__; g__; s__ |
| OTU_241 | k__Bacteria; p__GAL15; c__GAL15; o__GAL15; f__GAL15; g__GAL15; s__ |
| OTU_242 | k__Bacteria; p__Actinobacteriota; c__Actinobacteria; o__Corynebacteriales; f__Mycobacteriaceae; g__Mycobacterium |
| OTU_247 | k__Bacteria; p__Acidobacteriota; c__Holophagae; o__Subgroup_7; f__Subgroup_7; g__Subgroup_7; s__ |
| OTU_248 | k__Bacteria; p__Chloroflexi; c__Ktedonobacteria; o__Ktedonobacterales; f__JG30-KF-AS9; g__JG30-KF-AS9 |
| OTU_249 | k__Bacteria; p__Acidobacteriota; c__Acidobacteriae; o__Acidobacteriales; f__Acidobacteriaceae_(Subgroup_1); g__Occallatibacter; s__ |
| OTU_250 | k__Bacteria; p__Chloroflexi; c__Ktedonobacteria; o__Ktedonobacterales; f__JG30-KF-AS9; g__JG30-KF-AS9; s__ |
| OTU_252 | k__Bacteria; p__Proteobacteria; c__Gammaproteobacteria; o__Burkholderiales; f__Burkholderiaceae; g__Burkholderia-Caballeronia-Paraburkholderia |
| OTU_253 | k__Bacteria; p__Proteobacteria; c__Gammaproteobacteria; o__Gammaproteobacteria_Incertae_Sedis; f__Unknown_Family; g__Acidibacter; s__ |
| OTU_255 | k__Bacteria; p__Proteobacteria; c__Alphaproteobacteria; o__Caulobacterales; f__Hyphomonadaceae; g__SWB02; s__ |
| OTU_256 | k__Bacteria; p__Proteobacteria; c__Alphaproteobacteria; o__Acetobacterales; f__Acetobacteraceae |
| OTU_257 | k__Bacteria; p__Actinobacteriota; c__Actinobacteria; o__Frankiales; f__Acidothermaceae; g__Acidothermus; s__ |
| OTU_258 | k__Bacteria; p__Chloroflexi; c__Ktedonobacteria; o__Ktedonobacterales; f__Ktedonobacteraceae; g__; s__ |
| OTU_259 | k__Bacteria; p__Firmicutes; c__Bacilli; o__Bacillales; f__Bacillaceae; g__Anoxybacillus |
| OTU_260 | k__Bacteria; p__Proteobacteria; c__Alphaproteobacteria; o__Rhizobiales; f__Beijerinckiaceae; g__Roseiarcus; s__ |
| OTU_261 | k__Bacteria; p__Actinobacteriota; c__Thermoleophilia; o__Gaiellales; f__; g__; s__ |
| OTU_263 | k__Bacteria; p__Proteobacteria; c__Gammaproteobacteria; o__Gammaproteobacteria_Incertae_Sedis; f__Unknown_Family; g__Acidibacter |
| OTU_264 | k__Bacteria; p__Proteobacteria; c__Alphaproteobacteria; o__Sphingomonadales; f__Sphingomonadaceae; g__Parablastomonas; s__ |
| OTU_265 | k__Bacteria; p__Acidobacteriota; c__Acidobacteriae; o__Subgroup_2; f__Subgroup_2; g__Subgroup_2; s__ |
| OTU_266 | k__Bacteria; p__Proteobacteria; c__Gammaproteobacteria; o__Burkholderiales; f__Burkholderiaceae; g__Burkholderia-Caballeronia-Paraburkholderia |
| OTU_268 | k__Bacteria; p__Actinobacteriota; c__Actinobacteria; o__Streptosporangiales; f__Streptosporangiaceae; g__Nonomuraea; s__Nonomuraea_sp. |
| OTU_269 | k__Bacteria; p__Gemmatimonadota; c__Gemmatimonadetes; o__Gemmatimonadales; f__Gemmatimonadaceae; g__; s__ |
| OTU_270 | k__Bacteria; p__Proteobacteria; c__Alphaproteobacteria; o__Caulobacterales; f__Caulobacteraceae; g__Phenylobacterium; s__ |
| OTU_271 | k__Bacteria; p__Proteobacteria; c__Gammaproteobacteria; o__Burkholderiales; f__A21b; g__A21b; s__ |
| OTU_272 | k__Bacteria; p__Verrucomicrobiota; c__Verrucomicrobiae; o__Chthoniobacterales; f__Chthoniobacteraceae; g__Candidatus_Udaeobacter; s__ |
| OTU_274 | k__Bacteria; p__Chloroflexi; c__Anaerolineae; o__Anaerolineae; f__Anaerolineae; g__Anaerolineae; s__ |
| OTU_275 | k__Bacteria; p__Myxococcota; c__Polyangia; o__Haliangiales; f__Haliangiaceae; g__Haliangium; s__ |
| OTU_278 | k__Bacteria; p__Acidobacteriota; c__Acidobacteriae; o__Bryobacterales; f__Bryobacteraceae; g__Bryobacter; s__ |
| OTU_279 | k__Bacteria; p__Proteobacteria; c__Gammaproteobacteria; o__Burkholderiales; f__Burkholderiaceae; g__Burkholderia-Caballeronia-Paraburkholderia |
| OTU_280 | k__Bacteria; p__Bacteroidota; c__Bacteroidia; o__Chitinophagales; f__Chitinophagaceae; g__Puia; s__ |
| OTU_281 | k__Bacteria; p__Verrucomicrobiota; c__Verrucomicrobiae; o__Pedosphaerales; f__Pedosphaeraceae; g__; s__ |
| OTU_282 | k__Bacteria; p__Methylomirabilota; c__Methylomirabilia; o__Rokubacteriales; f__Rokubacteriales; g__Rokubacteriales; s__ |
| OTU_283 | k__Bacteria; p__Bacteroidota; c__Bacteroidia; o__Chitinophagales; f__Chitinophagaceae; g__; s__ |
| OTU_287 | k__Bacteria; p__Proteobacteria; c__Alphaproteobacteria; o__Rhizobiales; f__Methyloligellaceae; g__Methyloligellaceae; s__ |
| OTU_288 | k__Bacteria; p__Actinobacteriota; c__Acidimicrobiia |
| OTU_289 | k__Bacteria; p__Proteobacteria; c__Alphaproteobacteria; o__Acetobacterales; f__Acetobacteraceae; g__Acidiphilium; s__ |
| OTU_290 | k__Bacteria; p__Chloroflexi; c__KD4-96; o__KD4-96; f__KD4-96; g__KD4-96; s__ |
| OTU_291 | k__Bacteria; p__Acidobacteriota; c__Vicinamibacteria; o__Vicinamibacterales; f__; g__; s__ |
| OTU_292 | k__Bacteria; p__Myxococcota; c__Polyangia; o__Polyangiales; f__BIrii41; g__BIrii41; s__ |
| OTU_293 | k__Bacteria; p__Actinobacteriota; c__Actinobacteria; o__Micromonosporales; f__Micromonosporaceae; g__Dactylosporangium; s__ |
| OTU_294 | k__Bacteria; p__Acidobacteriota; c__Acidobacteriae; o__Subgroup_2; f__Subgroup_2; g__Subgroup_2; s__ |
| OTU_296 | k__Bacteria; p__Myxococcota; c__Polyangia; o__Haliangiales; f__Haliangiaceae; g__Haliangium; s__ |
| OTU_297 | k__Bacteria; p__Proteobacteria; c__Gammaproteobacteria; o__Gammaproteobacteria_Incertae_Sedis; f__Unknown_Family; g__Acidibacter; s__ |
| OTU_298 | k__Bacteria; p__Proteobacteria; c__Gammaproteobacteria; o__Burkholderiales; f__SC-I-84; g__SC-I-84 |
| OTU_300 | k__Bacteria; p__Proteobacteria; c__Gammaproteobacteria; o__Burkholderiales; f__Nitrosomonadaceae; g__IS-44; s__ |
| OTU_302 | k__Bacteria; p__RCP2-54; c__RCP2-54; o__RCP2-54; f__RCP2-54; g__RCP2-54; s__ |
| OTU_303 | k__Bacteria |
| OTU_304 | k__Bacteria; p__Chloroflexi; c__Ktedonobacteria; o__Ktedonobacterales; f__JG30-KF-AS9; g__JG30-KF-AS9; s__ |
| OTU_305 | k__Bacteria; p__Actinobacteriota; c__Actinobacteria; o__Kineosporiales; f__Kineosporiaceae |
| OTU_306 | k__Bacteria; p__Chloroflexi; c__Ktedonobacteria; o__Ktedonobacterales; f__JG30-KF-AS9; g__JG30-KF-AS9; s__ |
| OTU_307 | k__Bacteria; p__Chloroflexi; c__Ktedonobacteria; o__Ktedonobacterales; f__JG30-KF-AS9; g__JG30-KF-AS9; s__ |
| OTU_309 | k__Bacteria; p__Actinobacteriota; c__Actinobacteria; o__Frankiales; f__Nakamurellaceae; g__Nakamurella |
| OTU_310 | k__Bacteria; p__Acidobacteriota; c__Acidobacteriae; o__Solibacterales; f__Solibacteraceae; g__Candidatus_Solibacter; s__ |
| OTU_311 | k__Bacteria; p__Actinobacteriota; c__Acidimicrobiia; o__IMCC26256; f__IMCC26256; g__IMCC26256; s__ |
| OTU_312 | k__Bacteria; p__Proteobacteria; c__Alphaproteobacteria; o__Elsterales; f__; g__; s__ |
| OTU_313 | k__Bacteria; p__Verrucomicrobiota; c__Verrucomicrobiae; o__Pedosphaerales; f__Pedosphaeraceae; g__ADurb.Bin063-1; s__ |
| OTU_314 | k__Bacteria; p__Bacteroidota; c__Bacteroidia; o__Sphingobacteriales; f__Sphingobacteriaceae; g__Mucilaginibacter; s__ |
| OTU_316 | k__Bacteria; p__Gemmatimonadota; c__Gemmatimonadetes; o__Gemmatimonadales; f__Gemmatimonadaceae; g__; s__ |
| OTU_319 | k__Bacteria; p__Proteobacteria; c__Gammaproteobacteria; o__Burkholderiales; f__Comamonadaceae; g__Aquabacterium; s__ |
| OTU_321 | k__Bacteria; p__Proteobacteria; c__Alphaproteobacteria; o__Rhizobiales; f__Xanthobacteraceae; g__Xanthobacteraceae; s__Rhizobiales_bacterium |
| OTU_322 | k__Bacteria; p__Gemmatimonadota; c__Gemmatimonadetes; o__Gemmatimonadales; f__Gemmatimonadaceae; g__; s__ |
| OTU_323 | k__Bacteria; p__Bacteroidota; c__Bacteroidia; o__Chitinophagales; f__Chitinophagaceae; g__Puia; s__ |
| OTU_326 | k__Bacteria; p__Proteobacteria; c__Alphaproteobacteria; o__Caulobacterales; f__Caulobacteraceae; g__Phenylobacterium; s__ |
| OTU_327 | k__Bacteria; p__Myxococcota; c__Polyangia; o__Haliangiales; f__Haliangiaceae; g__Haliangium; s__ |
| OTU_328 | k__Bacteria; p__Acidobacteriota; c__Acidobacteriae; o__Bryobacterales; f__Bryobacteraceae; g__Bryobacter; s__ |
| OTU_329 | k__Bacteria; p__Acidobacteriota; c__Acidobacteriae; o__Acidobacteriales; f__Acidobacteriaceae_(Subgroup_1); g__Granulicella; s__ |
| OTU_330 | k__Bacteria; p__Chloroflexi; c__Dehalococcoidia; o__S085; f__S085; g__S085; s__ |
| OTU_332 | k__Bacteria; p__Actinobacteriota; c__Actinobacteria; o__Catenulisporales; f__Actinospicaceae; g__Actinospica |
| OTU_333 | k__Archaea; p__Crenarchaeota; c__Nitrososphaeria; o__Nitrososphaerales; f__Nitrososphaeraceae; g__Candidatus_Nitrocosmicus; s__ |
| OTU_334 | k__Bacteria; p__Actinobacteriota; c__MB-A2-108; o__MB-A2-108; f__MB-A2-108; g__MB-A2-108; s__ |
| OTU_336 | k__Bacteria; p__Proteobacteria; c__Gammaproteobacteria; o__Xanthomonadales; f__Rhodanobacteraceae; g__Rhodanobacter; s__ |
| OTU_337 | k__Bacteria; p__Actinobacteriota; c__Acidimicrobiia; o__IMCC26256; f__IMCC26256; g__IMCC26256; s__ |
| OTU_338 | k__Bacteria; p__Proteobacteria; c__Alphaproteobacteria; o__Rhizobiales; f__Rhizobiales_Incertae_Sedis; g__Alsobacter |
| OTU_339 | k__Bacteria |
| OTU_341 | k__Bacteria; p__Actinobacteriota; c__Acidimicrobiia; o__; f__; g__; s__ |
| OTU_342 | k__Bacteria; p__Myxococcota; c__Myxococcia; o__Myxococcales; f__Anaeromyxobacteraceae; g__Anaeromyxobacter; s__ |
| OTU_343 | k__Bacteria; p__Acidobacteriota; c__Acidobacteriae; o__Solibacterales; f__Solibacteraceae; g__Candidatus_Solibacter; s__ |
| OTU_344 | k__Bacteria; p__Acidobacteriota; c__Vicinamibacteria; o__Vicinamibacterales; f__; g__; s__ |
| OTU_345 | k__Bacteria; p__Chloroflexi; c__Chloroflexia; o__Chloroflexales; f__Roseiflexaceae; g__; s__ |
| OTU_346 | k__Bacteria; p__Proteobacteria; c__Gammaproteobacteria; o__Burkholderiales; f__SC-I-84; g__SC-I-84; s__ |
| OTU_347 | k__Bacteria; p__Proteobacteria; c__Alphaproteobacteria; o__Rhizobiales; f__Beijerinckiaceae; g__Roseiarcus; s__ |
| OTU_348 | k__Bacteria; p__Proteobacteria; c__Gammaproteobacteria; o__Burkholderiales; f__Nitrosomonadaceae; g__MND1; s__ |
| OTU_349 | k__Bacteria; p__Actinobacteriota; c__Thermoleophilia; o__Gaiellales |
| OTU_352 | k__Bacteria; p__Verrucomicrobiota; c__Verrucomicrobiae; o__Chthoniobacterales; f__Chthoniobacteraceae; g__Candidatus_Udaeobacter; s__ |
| OTU_353 | k__Bacteria; p__Acidobacteriota; c__Acidobacteriae; o__Acidobacteriales |
| OTU_354 | k__Bacteria; p__Acidobacteriota; c__Acidobacteriae; o__Subgroup_2; f__Subgroup_2; g__Subgroup_2; s__ |
| OTU_356 | k__Bacteria; p__Acidobacteriota; c__Holophagae; o__Subgroup_7; f__Subgroup_7; g__Subgroup_7; s__ |
| OTU_359 | k__Bacteria; p__Myxococcota; c__Myxococcia; o__Myxococcales; f__Anaeromyxobacteraceae; g__Anaeromyxobacter; s__ |
| OTU_360 | k__Bacteria; p__Actinobacteriota; c__Actinobacteria; o__Frankiales; f__Geodermatophilaceae |
| OTU_361 | k__Bacteria; p__Actinobacteriota; c__Thermoleophilia; o__Solirubrobacterales; f__67-14; g__67-14; s__ |
| OTU_362 | k__Bacteria; p__Proteobacteria; c__Alphaproteobacteria; o__Micropepsales; f__Micropepsaceae; g__; s__ |
| OTU_364 | k__Bacteria; p__Proteobacteria; c__Gammaproteobacteria; o__Salinisphaerales; f__Solimonadaceae; g__Nevskia |
| OTU_365 | k__Bacteria; p__Chloroflexi; c__P2-11E; o__P2-11E; f__P2-11E; g__P2-11E; s__ |
| OTU_368 | k__Bacteria; p__Chloroflexi; c__AD3; o__AD3; f__AD3; g__AD3; s__ |
| OTU_372 | k__Bacteria; p__Actinobacteriota; c__Thermoleophilia; o__Solirubrobacterales; f__Solirubrobacteraceae |
| OTU_373 | k__Bacteria; p__Bacteroidota; c__Bacteroidia; o__Chitinophagales; f__Chitinophagaceae; g__; s__ |
| OTU_374 | k__Bacteria; p__Proteobacteria; c__Gammaproteobacteria; o__Burkholderiales; f__Comamonadaceae |
| OTU_375 | k__Bacteria; p__Proteobacteria; c__Gammaproteobacteria; o__Burkholderiales; f__Sutterellaceae; g__; s__ |
| OTU_377 | k__Bacteria; p__Proteobacteria; c__Gammaproteobacteria; o__Burkholderiales; f__Nitrosomonadaceae; g__Ellin6067; s__ |
| OTU_378 | k__Bacteria; p__Acidobacteriota; c__Acidobacteriae; o__Bryobacterales; f__Bryobacteraceae; g__Bryobacter; s__ |
| OTU_379 | k__Bacteria; p__Myxococcota; c__Polyangia; o__Polyangiales; f__Polyangiaceae; g__Pajaroellobacter; s__ |
| OTU_381 | k__Bacteria; p__Proteobacteria; c__Gammaproteobacteria; o__Burkholderiales; f__TRA3-20; g__TRA3-20; s__ |
| OTU_382 | k__Bacteria; p__Proteobacteria; c__Gammaproteobacteria; o__Gammaproteobacteria_Incertae_Sedis; f__Unknown_Family; g__Acidibacter; s__ |
| OTU_383 | k__Bacteria; p__Proteobacteria; c__Gammaproteobacteria; o__Burkholderiales; f__Burkholderiaceae; g__Cupriavidus; s__Cupriavidus_basilensis |
| OTU_385 | k__Bacteria; p__Chloroflexi; c__Ktedonobacteria; o__Ktedonobacterales; f__Ktedonobacteraceae; g__Thermosporothrix; s__ |
| OTU_386 | k__Bacteria; p__Myxococcota; c__Polyangia; o__Haliangiales; f__Haliangiaceae; g__Haliangium; s__ |
| OTU_388 | k__Bacteria; p__Acidobacteriota; c__Blastocatellia; o__Pyrinomonadales; f__Pyrinomonadaceae; g__RB41; s__ |
| OTU_389 | k__Bacteria; p__Acidobacteriota; c__Acidobacteriae; o__Acidobacteriales; f__; g__; s__ |
| OTU_393 | k__Bacteria; p__Gemmatimonadota; c__Gemmatimonadetes; o__Gemmatimonadales; f__Gemmatimonadaceae; g__; s__ |
| OTU_394 | k__Bacteria; p__Actinobacteriota; c__Thermoleophilia; o__Solirubrobacterales; f__67-14; g__67-14; s__ |
| OTU_397 | k__Bacteria; p__Acidobacteriota; c__Vicinamibacteria; o__Vicinamibacterales; f__Vicinamibacteraceae; g__Vicinamibacteraceae; s__ |
| OTU_398 | k__Bacteria; p__Proteobacteria; c__Alphaproteobacteria; o__Rhizobiales; f__Beijerinckiaceae; g__Rhodoblastus; s__ |
| OTU_399 | k__Bacteria; p__Actinobacteriota; c__Actinobacteria |
| OTU_401 | k__Bacteria; p__Proteobacteria; c__Gammaproteobacteria; o__JG36-TzT-191; f__JG36-TzT-191; g__JG36-TzT-191; s__ |
| OTU_403 | k__Bacteria; p__Myxococcota; c__Polyangia; o__Polyangiales; f__BIrii41; g__BIrii41; s__ |
| OTU_404 | k__Bacteria; p__Acidobacteriota; c__Vicinamibacteria; o__Vicinamibacterales; f__; g__; s__ |
| OTU_405 | k__Bacteria; p__Acidobacteriota; c__Acidobacteriae; o__Solibacterales; f__Solibacteraceae; g__Candidatus_Solibacter; s__ |
| OTU_407 | k__Bacteria; p__Proteobacteria; c__Alphaproteobacteria; o__Elsterales; f__; g__; s__ |
| OTU_408 | k__Bacteria; p__Proteobacteria; c__Gammaproteobacteria; o__CCD24; f__CCD24; g__CCD24; s__ |
| OTU_410 | k__Bacteria; p__Actinobacteriota; c__Thermoleophilia; o__Gaiellales; f__; g__; s__ |
| OTU_412 | k__Bacteria; p__Nitrospirota; c__Nitrospiria; o__Nitrospirales; f__Nitrospiraceae; g__Nitrospira; s__ |
| OTU_415 | k__Bacteria; p__Proteobacteria; c__Alphaproteobacteria; o__Micropepsales; f__Micropepsaceae; g__; s__ |
| OTU_416 | k__Bacteria; p__Acidobacteriota; c__Acidobacteriae; o__Acidobacteriales; f__; g__; s__ |
| OTU_419 | k__Bacteria; p__Chloroflexi; c__TK10; o__TK10; f__TK10; g__TK10 |
| OTU_420 | k__Bacteria; p__Actinobacteriota; c__Thermoleophilia; o__Solirubrobacterales; f__67-14; g__67-14; s__ |
| OTU_421 | k__Bacteria; p__Verrucomicrobiota; c__Verrucomicrobiae; o__Chthoniobacterales; f__Chthoniobacteraceae; g__Candidatus_Udaeobacter; s__ |
| OTU_422 | k__Bacteria; p__Proteobacteria; c__Alphaproteobacteria; o__Caulobacterales; f__Caulobacteraceae; g__; s__ |
| OTU_423 | k__Bacteria; p__Gemmatimonadota; c__Gemmatimonadetes; o__Gemmatimonadales; f__Gemmatimonadaceae; g__Gemmatimonas; s__ |
| OTU_424 | k__Bacteria; p__Proteobacteria; c__Alphaproteobacteria; o__Caulobacterales; f__Caulobacteraceae; g__; s__ |
| OTU_425 | k__Bacteria; p__Myxococcota; c__Polyangia; o__Polyangiales; f__Sandaracinaceae; g__ |
| OTU_426 | k__Bacteria; p__Bacteroidota; c__Bacteroidia; o__Chitinophagales; f__Chitinophagaceae; g__Flavitalea; s__ |
| OTU_429 | k__Bacteria; p__Acidobacteriota; c__Vicinamibacteria; o__Vicinamibacterales; f__; g__; s__ |
| OTU_430 | k__Bacteria; p__Proteobacteria; c__Alphaproteobacteria; o__; f__; g__ |
| OTU_432 | k__Bacteria; p__Proteobacteria; c__Alphaproteobacteria; o__Rhizobiales; f__Rhizobiales_Incertae_Sedis; g__Nordella; s__Nordella_oligomobilis |
| OTU_433 | k__Bacteria; p__Proteobacteria; c__Alphaproteobacteria; o__Acetobacterales; f__Acetobacteraceae; g__Acidisoma |
| OTU_434 | k__Bacteria; p__Bacteroidota; c__Bacteroidia; o__Cytophagales; f__Microscillaceae |
| OTU_436 | k__Bacteria; p__Chloroflexi; c__KD4-96; o__KD4-96; f__KD4-96; g__KD4-96; s__ |
| OTU_438 | k__Bacteria; p__Actinobacteriota; c__Actinobacteria; o__Propionibacteriales; f__Nocardioidaceae; g__Kribbella; s__Kribbella_sp. |
| OTU_439 | k__Bacteria; p__Acidobacteriota; c__Acidobacteriae; o__Subgroup_2; f__Subgroup_2; g__Subgroup_2; s__ |
| OTU_440 | k__Bacteria; p__Actinobacteriota; c__Actinobacteria; o__Frankiales; f__Acidothermaceae; g__Acidothermus |
| OTU_441 | k__Bacteria; p__Myxococcota; c__Polyangia; o__Haliangiales; f__Haliangiaceae; g__Haliangium; s__ |
| OTU_442 | k__Bacteria; p__Proteobacteria; c__Alphaproteobacteria; o__Acetobacterales; f__Acetobacteraceae; g__Roseomonas |
| OTU_444 | k__Bacteria; p__Actinobacteriota; c__Actinobacteria; o__Pseudonocardiales; f__Pseudonocardiaceae; g__Pseudonocardia |
| OTU_445 | k__Bacteria; p__Proteobacteria; c__Alphaproteobacteria; o__Elsterales; f__; g__; s__ |
| OTU_446 | k__Bacteria; p__Myxococcota; c__Polyangia; o__Polyangiales; f__BIrii41; g__BIrii41; s__ |
| OTU_447 | k__Bacteria; p__Proteobacteria; c__Alphaproteobacteria; o__Rhizobiales; f__A0839; g__A0839; s__ |
| OTU_448 | k__Bacteria; p__Chloroflexi; c__Ktedonobacteria; o__Ktedonobacterales; f__Ktedonobacteraceae; g__1921-3; s__ |
| OTU_451 | k__Bacteria; p__Actinobacteriota; c__Actinobacteria; o__Frankiales; f__Frankiaceae; g__Jatrophihabitans |
| OTU_452 | k__Bacteria; p__Actinobacteriota; c__Thermoleophilia; o__Solirubrobacterales; f__Solirubrobacteraceae; g__Conexibacter; s__ |
| OTU_454 | k__Bacteria; p__Proteobacteria; c__Gammaproteobacteria; o__Gammaproteobacteria_Incertae_Sedis; f__Unknown_Family; g__Acidibacter; s__ |
| OTU_455 | k__Bacteria; p__Proteobacteria; c__Alphaproteobacteria; o__Azospirillales; f__Inquilinaceae; g__Inquilinus; s__ |
| OTU_456 | k__Bacteria; p__Chloroflexi; c__Chloroflexia; o__Thermomicrobiales; f__JG30-KF-CM45; g__JG30-KF-CM45 |
| OTU_457 | k__Bacteria; p__Proteobacteria; c__Alphaproteobacteria; o__; f__; g__ |
| OTU_458 | k__Bacteria; p__Proteobacteria; c__Alphaproteobacteria; o__Reyranellales; f__Reyranellaceae; g__Reyranella; s__ |
| OTU_459 | k__Bacteria; p__Proteobacteria; c__Alphaproteobacteria; o__Acetobacterales; f__Acetobacteraceae; g__; s__ |
| OTU_461 | k__Bacteria; p__Proteobacteria; c__Alphaproteobacteria; o__Rhizobiales; f__Beijerinckiaceae |
| OTU_464 | k__Bacteria; p__Chloroflexi; c__Ktedonobacteria; o__Ktedonobacterales; f__JG30-KF-AS9; g__JG30-KF-AS9; s__ |
| OTU_465 | k__Bacteria; p__Proteobacteria; c__Gammaproteobacteria; o__Burkholderiales; f__Comamonadaceae |
| OTU_466 | k__Bacteria; p__Actinobacteriota; c__Actinobacteria; o__Streptosporangiales; f__Streptosporangiaceae |
| OTU_468 | k__Bacteria; p__Acidobacteriota; c__Holophagae; o__Subgroup_7; f__Subgroup_7; g__Subgroup_7; s__ |
| OTU_470 | k__Bacteria; p__Acidobacteriota; c__Acidobacteriae; o__Subgroup_2; f__Subgroup_2; g__Subgroup_2; s__ |
| OTU_473 | k__Bacteria; p__Proteobacteria; c__Alphaproteobacteria; o__Micropepsales; f__Micropepsaceae; g__Micropepsis; s__ |
| OTU_474 | k__Bacteria; p__Proteobacteria; c__Gammaproteobacteria; o__Xanthomonadales; f__Rhodanobacteraceae; g__Rhodanobacter; s__ |
| OTU_477 | k__Bacteria; p__Actinobacteriota; c__Actinobacteria; o__Pseudonocardiales; f__Pseudonocardiaceae; g__Pseudonocardia; s__Pseudonocardia_sp. |
| OTU_479 | k__Bacteria; p__Proteobacteria; c__Gammaproteobacteria; o__Burkholderiales; f__Oxalobacteraceae; g__Collimonas; s__Collimonas_sp. |
| OTU_480 | k__Bacteria; p__Acidobacteriota; c__Acidobacteriae; o__Acidobacteriales |
| OTU_481 | k__Bacteria; p__Myxococcota; c__bacteriap25; o__bacteriap25; f__bacteriap25; g__bacteriap25; s__ |
| OTU_483 | k__Bacteria; p__Proteobacteria; c__Gammaproteobacteria; o__Burkholderiales; f__Oxalobacteraceae; g__Massilia |
| OTU_484 | k__Bacteria |
| OTU_488 | k__Bacteria; p__Actinobacteriota; c__Actinobacteria; o__Propionibacteriales; f__Nocardioidaceae; g__Nocardioides; s__ |
| OTU_489 | k__Bacteria; p__Myxococcota; c__Myxococcia; o__Myxococcales; f__Myxococcaceae; g__; s__ |
| OTU_490 | k__Bacteria |
| OTU_491 | k__Bacteria; p__Actinobacteriota; c__Actinobacteria; o__Corynebacteriales; f__Nocardiaceae; g__Nocardia |
| OTU_492 | k__Bacteria; p__Gemmatimonadota; c__Gemmatimonadetes; o__Gemmatimonadales; f__Gemmatimonadaceae; g__Gemmatimonas; s__ |
| OTU_493 | k__Bacteria; p__Bacteroidota; c__Bacteroidia; o__Sphingobacteriales; f__Sphingobacteriaceae; g__Mucilaginibacter; s__ |
| OTU_494 | k__Bacteria; p__Actinobacteriota; c__Actinobacteria; o__Micromonosporales; f__Micromonosporaceae; g__Actinoplanes; s__Actinoplanes_derwentensis |
| OTU_495 | k__Bacteria; p__Chloroflexi; c__Gitt-GS-136; o__Gitt-GS-136; f__Gitt-GS-136; g__Gitt-GS-136; s__ |
| OTU_499 | k__Bacteria; p__Acidobacteriota; c__Holophagae; o__Subgroup_7; f__Subgroup_7; g__Subgroup_7; s__ |
| OTU_500 | k__Bacteria; p__Actinobacteriota; c__Thermoleophilia; o__Solirubrobacterales; f__Solirubrobacteraceae; g__Solirubrobacter; s__ |
| OTU_501 | k__Bacteria; p__Proteobacteria; c__Gammaproteobacteria; o__Burkholderiales; f__Nitrosomonadaceae; g__mle1-7; s__ |
| OTU_502 | k__Bacteria; p__Chloroflexi; c__Ktedonobacteria; o__Ktedonobacterales; f__Ktedonobacteraceae |
| OTU_503 | k__Bacteria; p__Patescibacteria; c__Microgenomatia; o__Candidatus_Levybacteria; f__Candidatus_Levybacteria; g__Candidatus_Levybacteria; s__ |
| OTU_504 | k__Bacteria; p__Actinobacteriota; c__MB-A2-108; o__MB-A2-108; f__MB-A2-108; g__MB-A2-108; s__ |
| OTU_506 | k__Bacteria; p__Acidobacteriota; c__Acidobacteriae; o__Acidobacteriales; f__; g__; s__ |
| OTU_507 | k__Bacteria; p__Acidobacteriota; c__Acidobacteriae; o__Bryobacterales; f__Bryobacteraceae; g__Bryobacter; s__ |
| OTU_508 | k__Bacteria; p__Chloroflexi; c__Ktedonobacteria; o__Ktedonobacterales; f__Ktedonobacteraceae |
| OTU_509 | k__Bacteria; p__Actinobacteriota; c__Thermoleophilia; o__Gaiellales; f__Gaiellaceae; g__Gaiella; s__ |
| OTU_510 | k__Bacteria; p__Proteobacteria; c__Alphaproteobacteria; o__Elsterales; f__; g__; s__ |
| OTU_511 | k__Bacteria; p__Proteobacteria; c__Alphaproteobacteria; o__Rhizobiales; f__KF-JG30-B3; g__KF-JG30-B3; s__ |
| OTU_513 | k__Bacteria; p__Chloroflexi; c__Dehalococcoidia; o__S085; f__S085; g__S085; s__ |
| OTU_514 | k__Bacteria; p__Verrucomicrobiota; c__Verrucomicrobiae; o__Pedosphaerales; f__Pedosphaeraceae; g__Ellin516; s__ |
| OTU_516 | k__Bacteria; p__Chloroflexi; c__Ktedonobacteria; o__Ktedonobacterales; f__Ktedonobacteraceae; g__HSB_OF53-F07; s__ |
| OTU_517 | k__Bacteria; p__Chloroflexi; c__Ktedonobacteria; o__C0119; f__C0119; g__C0119; s__ |
| OTU_518 | k__Archaea; p__Crenarchaeota; c__Nitrososphaeria; o__Nitrosotaleales; f__Nitrosotaleaceae; g__Nitrosotaleaceae; s__ |
| OTU_519 | k__Bacteria; p__Acidobacteriota; c__Acidobacteriae; o__Acidobacteriales |
| OTU_520 | k__Bacteria; p__Proteobacteria; c__Gammaproteobacteria; o__Burkholderiales; f__Comamonadaceae |
| OTU_521 | k__Bacteria; p__Proteobacteria; c__Alphaproteobacteria |
| OTU_525 | k__Bacteria; p__Bacteroidota; c__Bacteroidia; o__Chitinophagales; f__Chitinophagaceae; g__Puia; s__ |
| OTU_526 | k__Bacteria; p__Acidobacteriota; c__Acidobacteriae; o__Acidobacteriales; f__Acidobacteriaceae_(Subgroup_1); g__Granulicella; s__ |
| OTU_529 | k__Bacteria; p__Myxococcota; c__Polyangia; o__Polyangiales; f__Sandaracinaceae; g__Sandaracinus; s__ |
| OTU_530 | k__Bacteria; p__Chloroflexi; c__JG30-KF-CM66; o__JG30-KF-CM66; f__JG30-KF-CM66; g__JG30-KF-CM66; s__ |
| OTU_531 | k__Bacteria; p__Proteobacteria; c__Gammaproteobacteria; o__Steroidobacterales; f__Steroidobacteraceae; g__Steroidobacter |
| OTU_532 | k__Bacteria; p__Gemmatimonadota; c__Gemmatimonadetes; o__Gemmatimonadales; f__Gemmatimonadaceae; g__Gemmatimonas; s__ |
| OTU_533 | k__Bacteria; p__Acidobacteriota; c__Acidobacteriae |
| OTU_534 | k__Bacteria; p__Actinobacteriota; c__Actinobacteria; o__Propionibacteriales; f__Nocardioidaceae |
| OTU_536 | k__Bacteria; p__Chloroflexi; c__Ktedonobacteria; o__Ktedonobacterales; f__Ktedonobacteraceae; g__HSB_OF53-F07; s__ |
| OTU_537 | k__Bacteria; p__Verrucomicrobiota; c__Verrucomicrobiae; o__Pedosphaerales; f__Pedosphaeraceae; g__; s__ |
| OTU_538 | k__Bacteria; p__Actinobacteriota; c__Thermoleophilia; o__Solirubrobacterales; f__67-14; g__67-14; s__ |
| OTU_543 | k__Bacteria; p__Bacteroidota; c__Bacteroidia; o__Chitinophagales; f__Chitinophagaceae; g__Flavisolibacter; s__ |
| OTU_546 | k__Bacteria; p__Proteobacteria; c__Gammaproteobacteria; o__Burkholderiales |
| OTU_548 | k__Bacteria; p__Acidobacteriota; c__Acidobacteriae; o__Subgroup_2; f__Subgroup_2; g__Subgroup_2; s__ |
| OTU_550 | k__Bacteria; p__Myxococcota; c__Myxococcia; o__Myxococcales; f__Anaeromyxobacteraceae; g__Anaeromyxobacter; s__ |
| OTU_552 | k__Bacteria; p__Myxococcota; c__Polyangia; o__Haliangiales; f__Haliangiaceae; g__Haliangium; s__ |
| OTU_556 | k__Bacteria; p__Proteobacteria; c__Gammaproteobacteria; o__Xanthomonadales; f__Rhodanobacteraceae; g__Dyella |
| OTU_557 | k__Bacteria; p__Myxococcota; c__Polyangia; o__Polyangiales; f__BIrii41; g__BIrii41; s__ |
| OTU_559 | k__Bacteria; p__Proteobacteria; c__Alphaproteobacteria; o__Elsterales; f__Elsteraceae; g__; s__ |
| OTU_561 | k__Bacteria; p__Chloroflexi; c__AD3; o__AD3; f__AD3; g__AD3; s__ |
| OTU_562 | k__Bacteria; p__Verrucomicrobiota; c__Verrucomicrobiae; o__Pedosphaerales; f__Pedosphaeraceae; g__Pedosphaera; s__ |
| OTU_563 | k__Bacteria; p__Chloroflexi; c__JG30-KF-CM66; o__JG30-KF-CM66; f__JG30-KF-CM66; g__JG30-KF-CM66; s__ |
| OTU_565 | k__Bacteria; p__Verrucomicrobiota; c__Verrucomicrobiae; o__Pedosphaerales; f__Pedosphaeraceae |
| OTU_566 | k__Bacteria; p__Chloroflexi; c__KD4-96; o__KD4-96; f__KD4-96; g__KD4-96; s__ |
| OTU_567 | k__Bacteria; p__Myxococcota; c__Polyangia; o__Polyangiales; f__Polyangiaceae; g__Pajaroellobacter; s__ |
| OTU_568 | k__Bacteria; p__Actinobacteriota; c__Thermoleophilia; o__Gaiellales; f__; g__ |
| OTU_570 | k__Bacteria; p__Proteobacteria; c__Gammaproteobacteria; o__KF-JG30-C25; f__KF-JG30-C25; g__KF-JG30-C25; s__ |
| OTU_571 | k__Bacteria; p__Actinobacteriota; c__Actinobacteria; o__Pseudonocardiales; f__Pseudonocardiaceae; g__Amycolatopsis |
| OTU_572 | k__Bacteria; p__Actinobacteriota; c__Actinobacteria; o__Catenulisporales; f__Actinospicaceae; g__Actinospica; s__Persicaria_minor |
| OTU_573 | k__Bacteria; p__Myxococcota; c__Polyangia; o__Haliangiales; f__Haliangiaceae; g__Haliangium; s__ |
| OTU_575 | k__Bacteria; p__Chloroflexi; c__SHA-26; o__SHA-26; f__SHA-26; g__SHA-26; s__ |
| OTU_576 | k__Bacteria; p__Firmicutes; c__Clostridia; o__Clostridiales; f__Clostridiaceae; g__Clostridium_sensu_stricto_9; s__ |
| OTU_578 | k__Bacteria; p__Proteobacteria; c__Alphaproteobacteria; o__Rhizobiales; f__Xanthobacteraceae; g__Rhodopseudomonas; s__ |
| OTU_579 | k__Bacteria; p__Proteobacteria; c__Gammaproteobacteria; o__Burkholderiales; f__SC-I-84; g__SC-I-84 |
| OTU_580 | k__Bacteria; p__Actinobacteriota; c__MB-A2-108; o__MB-A2-108; f__MB-A2-108; g__MB-A2-108; s__ |
| OTU_581 | k__Bacteria; p__Actinobacteriota; c__Acidimicrobiia; o__Microtrichales |
| OTU_582 | k__Bacteria; p__Nitrospirota; c__Nitrospiria; o__Nitrospirales; f__Nitrospiraceae; g__Nitrospira; s__ |
| OTU_585 | k__Bacteria; p__Gemmatimonadota; c__Gemmatimonadetes; o__Gemmatimonadales; f__Gemmatimonadaceae; g__Gemmatimonas; s__ |
| OTU_586 | k__Bacteria; p__WPS-2; c__WPS-2; o__WPS-2; f__WPS-2; g__WPS-2; s__ |
| OTU_587 | k__Bacteria; p__Acidobacteriota; c__Thermoanaerobaculia; o__Thermoanaerobaculales; f__Thermoanaerobaculaceae; g__Subgroup_10 |
| OTU_589 | k__Bacteria; p__Proteobacteria; c__Gammaproteobacteria; o__Burkholderiales; f__Nitrosomonadaceae; g__GOUTA6; s__ |
| OTU_590 | k__Bacteria; p__Actinobacteriota; c__MB-A2-108; o__MB-A2-108; f__MB-A2-108; g__MB-A2-108; s__ |
| OTU_591 | k__Bacteria; p__Gemmatimonadota; c__Gemmatimonadetes; o__Gemmatimonadales; f__Gemmatimonadaceae; g__Gemmatimonas; s__ |
| OTU_592 | k__Bacteria; p__Verrucomicrobiota; c__Verrucomicrobiae; o__Pedosphaerales; f__Pedosphaeraceae; g__Pedosphaeraceae; s__ |
| OTU_594 | k__Bacteria; p__Acidobacteriota; c__Vicinamibacteria; o__Vicinamibacterales; f__; g__; s__ |
| OTU_597 | k__Bacteria; p__Verrucomicrobiota; c__Verrucomicrobiae; o__Pedosphaerales; f__Pedosphaeraceae; g__; s__ |
| OTU_598 | k__Bacteria; p__Proteobacteria; c__Gammaproteobacteria; o__Burkholderiales; f__Oxalobacteraceae; g__Undibacterium |
| OTU_599 | k__Bacteria; p__Chloroflexi; c__Ktedonobacteria; o__Ktedonobacterales; f__JG30-KF-AS9; g__JG30-KF-AS9; s__ |
| OTU_600 | k__Bacteria; p__Actinobacteriota; c__Acidimicrobiia; o__Microtrichales; f__; g__; s__ |
| OTU_601 | k__Bacteria; p__Proteobacteria; c__Gammaproteobacteria; o__Salinisphaerales; f__Solimonadaceae |
| OTU_603 | k__Bacteria; p__Myxococcota; c__Polyangia; o__mle1-27; f__mle1-27; g__mle1-27 |
| OTU_604 | k__Bacteria; p__Acidobacteriota; c__Acidobacteriae; o__Acidobacteriales; f__Acidobacteriaceae_(Subgroup_1); g__Acidicapsa; s__Acidicapsa_sp. |
| OTU_608 | k__Bacteria; p__Proteobacteria; c__Alphaproteobacteria; o__Caulobacterales; f__Caulobacteraceae; g__Brevundimonas |
| OTU_609 | k__Bacteria; p__Chloroflexi; c__P2-11E; o__P2-11E; f__P2-11E; g__P2-11E; s__ |
| OTU_610 | k__Bacteria; p__Actinobacteriota; c__Actinobacteria; o__Corynebacteriales; f__Nocardiaceae; g__Nocardia |
| OTU_611 | k__Bacteria; p__Proteobacteria; c__Alphaproteobacteria; o__Micropepsales; f__Micropepsaceae; g__; s__ |
| OTU_612 | k__Bacteria; p__Chloroflexi; c__Ktedonobacteria; o__Ktedonobacterales; f__Ktedonobacteraceae; g__1921-2; s__ |
| OTU_613 | k__Bacteria; p__Gemmatimonadota; c__Gemmatimonadetes; o__Gemmatimonadales; f__Gemmatimonadaceae; g__; s__ |
| OTU_617 | k__Bacteria; p__Proteobacteria; c__Alphaproteobacteria; o__Rhodospirillales; f__Rhodospirillaceae; g__; s__ |
| OTU_619 | k__Bacteria; p__Gemmatimonadota; c__Gemmatimonadetes; o__Gemmatimonadales; f__Gemmatimonadaceae; g__Gemmatimonas; s__ |
| OTU_620 | k__Bacteria; p__Myxococcota; c__Myxococcia; o__Myxococcales; f__Myxococcaceae; g__Archangium |
| OTU_621 | k__Bacteria; p__Fibrobacterota; c__Fibrobacteria; o__Fibrobacterales; f__Fibrobacteraceae; g__possible_genus_04; s__ |
| OTU_622 | k__Bacteria; p__Actinobacteriota; c__Actinobacteria; o__Pseudonocardiales; f__Pseudonocardiaceae; g__Pseudonocardia |
| OTU_623 | k__Bacteria; p__Firmicutes; c__Bacilli; o__Paenibacillales; f__Paenibacillaceae; g__Paenibacillus; s__Paenibacillus_pectinilyticus |
| OTU_624 | k__Bacteria; p__Chloroflexi; c__KD4-96; o__KD4-96; f__KD4-96; g__KD4-96; s__ |
| OTU_625 | k__Bacteria; p__Proteobacteria; c__Alphaproteobacteria; o__Rhodospirillales; f__; g__; s__ |
| OTU_626 | k__Bacteria; p__Acidobacteriota; c__Acidobacteriae; o__Bryobacterales; f__Bryobacteraceae; g__Bryobacter; s__ |
| OTU_629 | k__Bacteria; p__Chloroflexi; c__Ktedonobacteria; o__Ktedonobacterales; f__Ktedonobacteraceae; g__Thermosporothrix; s__ |
| OTU_630 | k__Bacteria; p__Chloroflexi; c__AD3; o__AD3; f__AD3; g__AD3; s__ |
| OTU_631 | k__Bacteria; p__Proteobacteria; c__Gammaproteobacteria; o__Burkholderiales; f__Comamonadaceae |
| OTU_632 | k__Bacteria; p__Acidobacteriota; c__Thermoanaerobaculia; o__Thermoanaerobaculales; f__Thermoanaerobaculaceae; g__Subgroup_10; s__ |
| OTU_634 | k__Bacteria; p__Actinobacteriota; c__Acidimicrobiia; o__IMCC26256; f__IMCC26256; g__IMCC26256; s__ |
| OTU_635 | k__Bacteria; p__Acidobacteriota; c__Vicinamibacteria; o__Vicinamibacterales; f__; g__; s__ |
| OTU_636 | k__Bacteria; p__Chloroflexi; c__JG30-KF-CM66; o__JG30-KF-CM66; f__JG30-KF-CM66; g__JG30-KF-CM66; s__ |
| OTU_639 | k__Bacteria; p__Actinobacteriota; c__Actinobacteria; o__Micromonosporales; f__Micromonosporaceae; g__Actinoplanes; s__Actinoplanes_sp. |
| OTU_640 | k__Bacteria; p__Actinobacteriota; c__MB-A2-108; o__MB-A2-108; f__MB-A2-108; g__MB-A2-108; s__ |
| OTU_644 | k__Bacteria; p__Firmicutes; c__Bacilli; o__Paenibacillales; f__Paenibacillaceae; g__Paenibacillus; s__Paenibacillus_xylanexedens |
| OTU_647 | k__Bacteria; p__Actinobacteriota; c__Thermoleophilia; o__Solirubrobacterales; f__67-14; g__67-14; s__ |
| OTU_650 | k__Bacteria; p__Actinobacteriota; c__Actinobacteria; o__Pseudonocardiales; f__Pseudonocardiaceae; g__Pseudonocardia; s__ |
| OTU_653 | k__Bacteria; p__Actinobacteriota; c__Actinobacteria; o__Corynebacteriales; f__Nocardiaceae; g__Rhodococcus; s__Rhodococcus_erythropolis |
| OTU_657 | k__Bacteria; p__Acidobacteriota; c__Acidobacteriae; o__Acidobacteriales; f__Acidobacteriaceae_(Subgroup_1); g__; s__ |
| OTU_659 | k__Bacteria; p__Proteobacteria; c__Alphaproteobacteria; o__Elsterales; f__; g__; s__ |
| OTU_662 | k__Bacteria; p__Firmicutes; c__Bacilli; o__Bacillales; f__Bacillaceae; g__Bacillus; s__Bacillus_simplex |
| OTU_663 | k__Bacteria; p__Acidobacteriota; c__Thermoanaerobaculia; o__Thermoanaerobaculales; f__Thermoanaerobaculaceae; g__Subgroup_10; s__ |
| OTU_664 | k__Bacteria; p__Proteobacteria; c__Alphaproteobacteria; o__Rhodospirillales; f__; g__; s__ |
| OTU_665 | k__Bacteria; p__Actinobacteriota; c__Acidimicrobiia; o__IMCC26256; f__IMCC26256; g__IMCC26256; s__actinobacterium_BGR |
| OTU_668 | k__Bacteria; p__Firmicutes; c__Bacilli; o__Paenibacillales; f__Paenibacillaceae; g__Ammoniphilus; s__ |
| OTU_672 | k__Bacteria; p__Actinobacteriota; c__Actinobacteria; o__Pseudonocardiales; f__Pseudonocardiaceae; g__Actinomycetospora; s__ |
| OTU_673 | k__Bacteria; p__Acidobacteriota; c__Acidobacteriae; o__Subgroup_13; f__Subgroup_13; g__Subgroup_13; s__ |
| OTU_677 | k__Bacteria; p__Proteobacteria; c__Gammaproteobacteria; o__Steroidobacterales; f__Steroidobacteraceae; g__; s__ |
| OTU_680 | k__Bacteria; p__Actinobacteriota; c__MB-A2-108; o__MB-A2-108; f__MB-A2-108; g__MB-A2-108; s__ |
| OTU_681 | k__Bacteria; p__Actinobacteriota; c__Actinobacteria; o__Frankiales; f__Nakamurellaceae; g__Nakamurella; s__ |
| OTU_682 | k__Bacteria; p__Actinobacteriota; c__Actinobacteria; o__Micrococcales; f__Cellulomonadaceae; g__Cellulomonas; s__Cellulomonas_sp. |
| OTU_683 | k__Bacteria; p__Actinobacteriota; c__Thermoleophilia; o__Solirubrobacterales; f__Solirubrobacteraceae; g__Solirubrobacter |
| OTU_687 | k__Bacteria; p__Actinobacteriota; c__Thermoleophilia; o__Gaiellales; f__; g__ |
| OTU_688 | k__Bacteria; p__Proteobacteria; c__Alphaproteobacteria; o__Rhizobiales; f__Beijerinckiaceae |
| OTU_690 | k__Bacteria; p__Actinobacteriota; c__Acidimicrobiia; o__IMCC26256; f__IMCC26256; g__IMCC26256; s__ |
| OTU_694 | k__Bacteria; p__Myxococcota; c__Polyangia; o__Polyangiales; f__Polyangiaceae; g__Pajaroellobacter; s__ |
| OTU_695 | k__Bacteria; p__Planctomycetota; c__Planctomycetes; o__Isosphaerales; f__Isosphaeraceae; g__Aquisphaera; s__ |
| OTU_696 | k__Bacteria; p__Proteobacteria; c__Gammaproteobacteria; o__Burkholderiales; f__Nitrosomonadaceae; g__Ellin6067; s__ |
| OTU_697 | k__Bacteria; p__Proteobacteria; c__Gammaproteobacteria; o__Pseudomonadales; f__Pseudomonadaceae; g__Pseudomonas; s__Pseudomonas_vancouverensis |
| OTU_698 | k__Bacteria; p__Chloroflexi; c__Ktedonobacteria; o__Ktedonobacterales; f__Ktedonobacteraceae |
| OTU_699 | k__Bacteria; p__Proteobacteria; c__Gammaproteobacteria; o__Gammaproteobacteria_Incertae_Sedis; f__Unknown_Family; g__Acidibacter; s__ |
| OTU_701 | k__Bacteria; p__Gemmatimonadota; c__Gemmatimonadetes; o__Gemmatimonadales; f__Gemmatimonadaceae; g__; s__ |
| OTU_703 | k__Bacteria; p__Proteobacteria; c__Alphaproteobacteria; o__Caulobacterales; f__Caulobacteraceae; g__ |
| OTU_705 | k__Bacteria; p__Methylomirabilota; c__Methylomirabilia; o__Rokubacteriales; f__Rokubacteriales; g__Rokubacteriales; s__ |
| OTU_708 | k__Bacteria; p__Planctomycetota; c__Phycisphaerae; o__Tepidisphaerales; f__WD2101_soil_group; g__WD2101_soil_group; s__ |
| OTU_709 | k__Bacteria; p__Acidobacteriota; c__Holophagae; o__Subgroup_7; f__Subgroup_7; g__Subgroup_7; s__ |
| OTU_711 | k__Bacteria; p__Actinobacteriota; c__Acidimicrobiia |
| OTU_713 | k__Bacteria; p__WPS-2; c__WPS-2; o__WPS-2; f__WPS-2; g__WPS-2; s__ |
| OTU_714 | k__Bacteria |
| OTU_715 | k__Bacteria; p__Actinobacteriota; c__Acidimicrobiia; o__Microtrichales; f__; g__; s__ |
| OTU_716 | k__Bacteria; p__Proteobacteria; c__Alphaproteobacteria; o__Dongiales; f__Dongiaceae; g__Dongia; s__ |
| OTU_719 | k__Bacteria; p__Actinobacteriota; c__Thermoleophilia; o__Gaiellales; f__; g__; s__bacterium_Ellin6515 |
| OTU_722 | k__Bacteria; p__Actinobacteriota; c__Thermoleophilia; o__; f__; g__; s__ |
| OTU_723 | k__Bacteria; p__Proteobacteria; c__Alphaproteobacteria; o__Acetobacterales; f__Acetobacteraceae |
| OTU_724 | k__Bacteria; p__Gemmatimonadota; c__Gemmatimonadetes; o__Gemmatimonadales; f__Gemmatimonadaceae; g__; s__ |
| OTU_726 | k__Bacteria; p__Myxococcota; c__Polyangia; o__Haliangiales; f__Haliangiaceae; g__Haliangium; s__ |
| OTU_728 | k__Bacteria; p__Verrucomicrobiota; c__Verrucomicrobiae; o__Pedosphaerales; f__Pedosphaeraceae; g__Pedosphaeraceae; s__ |
| OTU_729 | k__Archaea; p__Crenarchaeota; c__Nitrososphaeria; o__Nitrosotaleales; f__Nitrosotaleaceae; g__Nitrosotaleaceae; s__ |
| OTU_733 | k__Bacteria; p__Actinobacteriota; c__Thermoleophilia; o__Solirubrobacterales; f__Solirubrobacteraceae; g__; s__ |
| OTU_735 | k__Bacteria; p__Chloroflexi; c__Chloroflexia; o__Chloroflexales; f__Roseiflexaceae; g__; s__ |
| OTU_737 | k__Bacteria; p__Bacteroidota; c__Bacteroidia; o__Sphingobacteriales; f__Sphingobacteriaceae; g__Mucilaginibacter |
| OTU_738 | k__Bacteria; p__Verrucomicrobiota; c__Verrucomicrobiae; o__Chthoniobacterales; f__Chthoniobacteraceae; g__Candidatus_Udaeobacter; s__ |
| OTU_739 | k__Bacteria; p__Actinobacteriota; c__Acidimicrobiia; o__Microtrichales; f__Ilumatobacteraceae; g__; s__ |
| OTU_740 | k__Bacteria; p__Bacteroidota; c__Bacteroidia; o__Chitinophagales; f__Chitinophagaceae; g__Puia; s__ |
| OTU_741 | k__Bacteria; p__Proteobacteria; c__Alphaproteobacteria; o__; f__; g__; s__ |
| OTU_742 | k__Bacteria; p__Nitrospirota; c__Nitrospiria; o__Nitrospirales; f__Nitrospiraceae; g__Nitrospira |
| OTU_745 | k__Bacteria; p__Actinobacteriota; c__Actinobacteria; o__Streptomycetales; f__Streptomycetaceae; g__Streptomyces |
| OTU_747 | k__Bacteria; p__Proteobacteria; c__Alphaproteobacteria; o__Elsterales; f__; g__; s__ |
| OTU_748 | k__Bacteria; p__Acidobacteriota; c__Acidobacteriae; o__Bryobacterales; f__Bryobacteraceae; g__Bryobacter; s__ |
| OTU_749 | k__Bacteria; p__Proteobacteria; c__Alphaproteobacteria; o__Elsterales |
| OTU_751 | k__Bacteria; p__WPS-2; c__WPS-2; o__WPS-2; f__WPS-2; g__WPS-2; s__ |
| OTU_753 | k__Bacteria; p__Proteobacteria; c__Gammaproteobacteria; o__Xanthomonadales; f__Rhodanobacteraceae; g__Rhodanobacter; s__ |
| OTU_755 | k__Bacteria; p__Proteobacteria; c__Gammaproteobacteria; o__Xanthomonadales |
| OTU_757 | k__Bacteria; p__Acidobacteriota; c__Vicinamibacteria; o__Subgroup_17; f__Subgroup_17; g__Subgroup_17; s__ |
| OTU_758 | k__Bacteria; p__Acidobacteriota; c__Acidobacteriae; o__Subgroup_2; f__Subgroup_2; g__Subgroup_2; s__ |
| OTU_759 | k__Bacteria; p__Myxococcota; c__Polyangia; o__Haliangiales; f__Haliangiaceae; g__Haliangium; s__ |
| OTU_760 | k__Bacteria |
| OTU_762 | k__Bacteria; p__Acidobacteriota; c__Subgroup_5; o__Subgroup_5; f__Subgroup_5; g__Subgroup_5; s__ |
| OTU_764 | k__Bacteria; p__Actinobacteriota; c__MB-A2-108; o__MB-A2-108; f__MB-A2-108; g__MB-A2-108; s__ |
| OTU_765 | k__Bacteria; p__Proteobacteria; c__Alphaproteobacteria; o__Reyranellales; f__Reyranellaceae; g__; s__ |
| OTU_766 | k__Bacteria; p__Acidobacteriota; c__Blastocatellia; o__Pyrinomonadales; f__Pyrinomonadaceae; g__RB41; s__ |
| OTU_772 | k__Bacteria; p__Proteobacteria; c__Alphaproteobacteria; o__Rhizobiales; f__Rhizobiales_Incertae_Sedis; g__Nordella; s__ |
| OTU_774 | k__Bacteria; p__Proteobacteria; c__Gammaproteobacteria; o__Burkholderiales; f__Nitrosomonadaceae; g__MND1; s__ |
| OTU_776 | k__Bacteria; p__Fibrobacterota; c__Fibrobacteria; o__Fibrobacterales; f__Fibrobacteraceae; g__; s__bacterium_enrichment |
| OTU_778 | k__Bacteria; p__Actinobacteriota; c__Thermoleophilia; o__Gaiellales; f__; g__; s__ |
| OTU_780 | k__Bacteria; p__Acidobacteriota; c__Thermoanaerobaculia; o__Thermoanaerobaculales; f__Thermoanaerobaculaceae; g__Subgroup_10; s__Acidobacteria_bacterium |
| OTU_781 | k__Bacteria; p__Proteobacteria; c__Gammaproteobacteria; o__WD260; f__WD260; g__WD260; s__ |
| OTU_783 | k__Bacteria; p__Proteobacteria; c__Alphaproteobacteria; o__Rhizobiales; f__Hyphomicrobiaceae; g__Pedomicrobium; s__ |
| OTU_790 | k__Bacteria; p__Firmicutes; c__Bacilli; o__Alicyclobacillales; f__Alicyclobacillaceae; g__Tumebacillus |
| OTU_792 | k__Bacteria; p__Proteobacteria; c__Gammaproteobacteria; o__Burkholderiales; f__Oxalobacteraceae; g__Noviherbaspirillum; s__ |
| OTU_794 | k__Bacteria; p__Myxococcota; c__Polyangia; o__Haliangiales; f__Haliangiaceae; g__Haliangium; s__ |
| OTU_797 | k__Bacteria; p__Proteobacteria; c__Alphaproteobacteria; o__Sphingomonadales; f__Sphingomonadaceae; g__Sphingomonas |
| OTU_799 | k__Bacteria; p__Actinobacteriota; c__Thermoleophilia; o__Gaiellales; f__; g__; s__ |
| OTU_800 | k__Bacteria; p__Latescibacterota; c__Latescibacterota; o__Latescibacterota; f__Latescibacterota; g__Latescibacterota; s__ |
| OTU_801 | k__Bacteria; p__Actinobacteriota; c__Thermoleophilia; o__Gaiellales; f__; g__; s__ |
| OTU_802 | k__Bacteria; p__Actinobacteriota; c__Thermoleophilia; o__Solirubrobacterales; f__Solirubrobacteraceae; g__Conexibacter; s__ |
| OTU_803 | k__Bacteria; p__Gemmatimonadota; c__Gemmatimonadetes; o__Gemmatimonadales; f__Gemmatimonadaceae; g__; s__ |
| OTU_805 | k__Bacteria; p__Desulfobacterota; c__; o__; f__; g__ |
| OTU_806 | k__Bacteria; p__Chloroflexi; c__Ktedonobacteria; o__Ktedonobacterales; f__Ktedonobacteraceae; g__HSB_OF53-F07; s__ |
| OTU_808 | k__Bacteria; p__Myxococcota; c__Polyangia; o__Polyangiales; f__Polyangiaceae; g__Minicystis; s__ |
| OTU_812 | k__Bacteria; p__Chloroflexi; c__KD4-96; o__KD4-96; f__KD4-96; g__KD4-96 |
| OTU_813 | k__Bacteria; p__Proteobacteria; c__Alphaproteobacteria; o__Rhizobiales; f__Rhizobiaceae; g__Mesorhizobium; s__Mesorhizobium_ciceri |
| OTU_814 | k__Bacteria; p__WPS-2; c__WPS-2; o__WPS-2; f__WPS-2; g__WPS-2; s__ |
| OTU_816 | k__Bacteria; p__Verrucomicrobiota; c__Verrucomicrobiae; o__Opitutales; f__Opitutaceae; g__Opitutus; s__ |
| OTU_817 | k__Bacteria; p__Myxococcota; c__Polyangia; o__Polyangiales; f__Polyangiaceae; g__Sorangium; s__ |
| OTU_818 | k__Bacteria; p__Myxococcota; c__Polyangia; o__Haliangiales; f__Haliangiaceae; g__Haliangium; s__ |
| OTU_820 | k__Bacteria; p__Proteobacteria; c__Gammaproteobacteria; o__KF-JG30-C25; f__KF-JG30-C25; g__KF-JG30-C25; s__ |
| OTU_822 | k__Bacteria; p__Actinobacteriota; c__MB-A2-108; o__MB-A2-108; f__MB-A2-108; g__MB-A2-108; s__ |
| OTU_823 | k__Bacteria; p__Verrucomicrobiota; c__Verrucomicrobiae; o__Pedosphaerales; f__Pedosphaeraceae; g__Pedosphaeraceae; s__ |
| OTU_826 | k__Bacteria; p__Bacteroidota; c__Bacteroidia; o__Cytophagales; f__Microscillaceae; g__ |
| OTU_830 | k__Bacteria; p__Acidobacteriota; c__Holophagae; o__Holophagales; f__Holophagaceae; g__Holophaga; s__ |
| OTU_832 | k__Bacteria; p__Proteobacteria; c__Alphaproteobacteria; o__Elsterales; f__Elsteraceae; g__; s__ |
| OTU_836 | k__Bacteria; p__Bacteroidota; c__Bacteroidia; o__Chitinophagales; f__Chitinophagaceae; g__Niastella; s__ |
| OTU_837 | k__Bacteria; p__Acidobacteriota; c__Blastocatellia; o__11-24; f__11-24; g__11-24; s__ |
| OTU_838 | k__Bacteria; p__Proteobacteria; c__Alphaproteobacteria; o__Rhizobiales; f__Beijerinckiaceae; g__Bosea |
| OTU_842 | k__Bacteria; p__Proteobacteria; c__Alphaproteobacteria; o__Acetobacterales; f__Acetobacteraceae; g__Acidicaldus; s__ |
| OTU_846 | k__Bacteria; p__Proteobacteria; c__Gammaproteobacteria; o__Burkholderiales; f__Comamonadaceae; g__Variovorax |
| OTU_847 | k__Bacteria; p__RCP2-54; c__RCP2-54; o__RCP2-54; f__RCP2-54; g__RCP2-54; s__ |
| OTU_850 | k__Bacteria; p__Myxococcota; c__Polyangia; o__Polyangiales; f__Sandaracinaceae; g__; s__ |
| OTU_851 | k__Bacteria; p__Acidobacteriota; c__Acidobacteriae; o__Subgroup_2; f__Subgroup_2; g__Subgroup_2; s__ |
| OTU_853 | k__Bacteria; p__Proteobacteria; c__Alphaproteobacteria; o__Rhizobiales; f__Rhizobiales_Incertae_Sedis; g__Bauldia; s__ |
| OTU_854 | k__Bacteria; p__Actinobacteriota; c__MB-A2-108; o__MB-A2-108; f__MB-A2-108; g__MB-A2-108; s__ |
| OTU_856 | k__Bacteria; p__Proteobacteria; c__Alphaproteobacteria; o__Elsterales; f__Elsteraceae; g__; s__ |
| OTU_858 | k__Archaea; p__Crenarchaeota; c__Nitrososphaeria; o__Group_1.1c; f__Group_1.1c; g__Group_1.1c; s__ |
| OTU_859 | k__Bacteria; p__Proteobacteria; c__Gammaproteobacteria; o__Burkholderiales; f__Oxalobacteraceae; g__Janthinobacterium |
| OTU_865 | k__Bacteria; p__Actinobacteriota; c__Acidimicrobiia; o__IMCC26256; f__IMCC26256; g__IMCC26256; s__ |
| OTU_866 | k__Bacteria; p__Myxococcota; c__Polyangia; o__Polyangiales; f__Polyangiaceae; g__Pajaroellobacter; s__ |
| OTU_870 | k__Bacteria; p__Verrucomicrobiota; c__Verrucomicrobiae; o__Pedosphaerales; f__Pedosphaeraceae; g__; s__ |
| OTU_871 | k__Bacteria; p__Actinobacteriota; c__Actinobacteria; o__Propionibacteriales; f__Nocardioidaceae; g__Nocardioides; s__Nocardioides_sp. |
| OTU_875 | k__Bacteria; p__Actinobacteriota; c__Acidimicrobiia; o__IMCC26256; f__IMCC26256; g__IMCC26256; s__ |
| OTU_876 | k__Bacteria; p__Acidobacteriota; c__Vicinamibacteria; o__Vicinamibacterales; f__; g__; s__ |
| OTU_878 | k__Bacteria; p__Proteobacteria; c__Alphaproteobacteria; o__Caulobacterales; f__Caulobacteraceae |
| OTU_879 | k__Bacteria; p__Actinobacteriota; c__Thermoleophilia; o__Solirubrobacterales; f__Solirubrobacteraceae; g__Conexibacter; s__ |
| OTU_880 | k__Bacteria; p__Verrucomicrobiota; c__Verrucomicrobiae; o__Pedosphaerales; f__Pedosphaeraceae; g__Pedosphaeraceae; s__ |
| OTU_881 | k__Bacteria; p__Proteobacteria; c__Alphaproteobacteria; o__Caulobacterales; f__Caulobacteraceae; g__; s__ |
| OTU_882 | k__Bacteria; p__Methylomirabilota; c__Methylomirabilia; o__Rokubacteriales; f__Rokubacteriales; g__Rokubacteriales; s__ |
| OTU_885 | k__Bacteria; p__Gemmatimonadota; c__Gemmatimonadetes; o__Gemmatimonadales; f__Gemmatimonadaceae; g__Gemmatimonas; s__ |
| OTU_898 | k__Bacteria; p__Actinobacteriota; c__Thermoleophilia; o__Gaiellales; f__; g__; s__ |
| OTU_902 | k__Bacteria; p__Myxococcota; c__Polyangia; o__Haliangiales; f__Haliangiaceae; g__Haliangium; s__ |
| OTU_903 | k__Bacteria; p__Chloroflexi; c__AD3; o__AD3; f__AD3; g__AD3; s__ |
| OTU_906 | k__Bacteria; p__Proteobacteria; c__Alphaproteobacteria; o__Micropepsales; f__Micropepsaceae; g__; s__ |
| OTU_910 | k__Bacteria; p__Desulfobacterota; c__; o__; f__; g__ |
| OTU_911 | k__Bacteria; p__Actinobacteriota; c__Thermoleophilia; o__Gaiellales; f__; g__; s__ |
| OTU_912 | k__Bacteria; p__Acidobacteriota; c__Acidobacteriae; o__Acidobacteriales; f__Koribacteraceae; g__Candidatus_Koribacter |
| OTU_913 | k__Bacteria; p__Gemmatimonadota; c__Gemmatimonadetes; o__Gemmatimonadales; f__Gemmatimonadaceae |
| OTU_914 | k__Bacteria; p__Actinobacteriota; c__MB-A2-108; o__MB-A2-108; f__MB-A2-108; g__MB-A2-108; s__ |
| OTU_915 | k__Bacteria; p__Actinobacteriota; c__Thermoleophilia; o__Solirubrobacterales; f__67-14; g__67-14; s__ |
| OTU_917 | k__Bacteria; p__Bdellovibrionota; c__Oligoflexia; o__0319-6G20; f__0319-6G20; g__0319-6G20 |
| OTU_918 | k__Bacteria; p__Latescibacterota; c__Latescibacterota; o__Latescibacterota; f__Latescibacterota; g__Latescibacterota; s__ |
| OTU_923 | k__Bacteria; p__Acidobacteriota; c__Acidobacteriae; o__Solibacterales; f__Solibacteraceae; g__Candidatus_Solibacter; s__ |
| OTU_925 | k__Bacteria; p__Actinobacteriota; c__MB-A2-108; o__MB-A2-108; f__MB-A2-108; g__MB-A2-108; s__ |
| OTU_926 | k__Bacteria; p__Proteobacteria; c__Gammaproteobacteria; o__Burkholderiales; f__Comamonadaceae; g__Rubrivivax; s__ |
| OTU_929 | k__Bacteria; p__Acidobacteriota; c__Acidobacteriae; o__Subgroup_13; f__Subgroup_13; g__Subgroup_13; s__ |
| OTU_938 | k__Bacteria; p__Acidobacteriota; c__Acidobacteriae; o__Bryobacterales; f__Bryobacteraceae; g__Bryobacter; s__ |
| OTU_941 | k__Bacteria; p__Chloroflexi; c__Ktedonobacteria; o__Ktedonobacterales; f__JG30-KF-AS9; g__JG30-KF-AS9; s__ |
| OTU_946 | k__Bacteria; p__Myxococcota; c__Polyangia; o__Polyangiales; f__Polyangiaceae; g__Pajaroellobacter; s__ |
| OTU_948 | k__Bacteria; p__Actinobacteriota; c__Acidimicrobiia; o__; f__; g__; s__ |
| OTU_949 | k__Bacteria; p__Myxococcota; c__Polyangia |
| OTU_950 | k__Bacteria; p__Actinobacteriota; c__Thermoleophilia; o__Gaiellales; f__; g__ |
| OTU_952 | k__Bacteria; p__Actinobacteriota; c__Actinobacteria; o__Frankiales; f__Cryptosporangiaceae; g__Cryptosporangium; s__Cryptosporangium_sp. |
| OTU_954 | k__Bacteria; p__Actinobacteriota; c__Thermoleophilia; o__Gaiellales; f__; g__ |
| OTU_955 | k__Bacteria; p__Actinobacteriota; c__Acidimicrobiia; o__Microtrichales; f__; g__; s__ |
| OTU_956 | k__Bacteria; p__Acidobacteriota; c__Vicinamibacteria; o__Vicinamibacterales; f__; g__; s__ |
| OTU_957 | k__Bacteria; p__Firmicutes; c__Bacilli; o__Bacillales; f__Planococcaceae; g__Psychrobacillus |
| OTU_959 | k__Bacteria; p__Verrucomicrobiota; c__Verrucomicrobiae; o__Opitutales; f__Opitutaceae; g__Lacunisphaera; s__ |
| OTU_960 | k__Bacteria; p__Actinobacteriota; c__Actinobacteria; o__Frankiales; f__Acidothermaceae; g__Acidothermus; s__ |
| OTU_961 | k__Bacteria; p__Verrucomicrobiota; c__Verrucomicrobiae; o__Opitutales; f__Opitutaceae |
| OTU_963 | k__Bacteria; p__Actinobacteriota; c__Actinobacteria; o__Pseudonocardiales; f__Pseudonocardiaceae; g__Actinophytocola; s__ |
| OTU_966 | k__Bacteria; p__Acidobacteriota; c__Acidobacteriae; o__Acidobacteriales; f__; g__; s__ |
| OTU_970 | k__Bacteria; p__Desulfobacterota; c__; o__; f__; g__; s__ |
| OTU_974 | k__Archaea; p__Crenarchaeota; c__Nitrososphaeria; o__Group_1.1c; f__Group_1.1c; g__Group_1.1c; s__ |
| OTU_977 | k__Bacteria; p__Chloroflexi; c__Chloroflexia; o__Chloroflexales; f__Roseiflexaceae; g__; s__ |
| OTU_978 | k__Bacteria; p__Verrucomicrobiota; c__Verrucomicrobiae; o__Chthoniobacterales; f__Chthoniobacteraceae; g__Chthoniobacter |
| OTU_981 | k__Bacteria; p__Actinobacteriota; c__Thermoleophilia; o__Gaiellales; f__; g__; s__ |
| OTU_982 | k__Bacteria; p__Proteobacteria; c__Alphaproteobacteria; o__Elsterales; f__; g__; s__ |
| OTU_987 | k__Bacteria; p__Acidobacteriota; c__Vicinamibacteria; o__Vicinamibacterales; f__; g__; s__ |
| OTU_988 | k__Bacteria; p__Proteobacteria; c__Alphaproteobacteria; o__Acetobacterales; f__Acetobacteraceae |
| OTU_992 | k__Bacteria; p__Gemmatimonadota; c__Gemmatimonadetes; o__Gemmatimonadales; f__Gemmatimonadaceae |
| OTU_996 | k__Bacteria; p__Firmicutes; c__Bacilli; o__Bacillales; f__Sporolactobacillaceae; g__Pullulanibacillus; s__ |
| OTU_999 | k__Bacteria; p__Proteobacteria; c__Alphaproteobacteria; o__Acetobacterales; f__Acetobacteraceae |
| OTU_1004 | k__Bacteria; p__Proteobacteria; c__Alphaproteobacteria; o__Caulobacterales; f__Caulobacteraceae; g__Asticcacaulis |
| OTU_1009 | k__Bacteria; p__Proteobacteria; c__Alphaproteobacteria; o__Elsterales; f__; g__; s__ |
| OTU_1018 | k__Bacteria; p__Acidobacteriota; c__Vicinamibacteria; o__Vicinamibacterales; f__; g__; s__ |
| OTU_1023 | k__Bacteria; p__Firmicutes; c__Bacilli; o__Paenibacillales; f__Paenibacillaceae; g__Paenibacillus; s__ |
| OTU_1029 | k__Bacteria; p__Chloroflexi; c__TK10; o__TK10; f__TK10; g__TK10; s__ |
| OTU_1031 | k__Bacteria; p__Chloroflexi; c__OLB14; o__OLB14; f__OLB14; g__OLB14; s__ |
| OTU_1033 | k__Bacteria; p__Proteobacteria; c__Alphaproteobacteria; o__Acetobacterales; f__Acetobacteraceae; g__Endobacter; s__ |
| OTU_1034 | k__Bacteria; p__Proteobacteria; c__Alphaproteobacteria; o__Rhizobiales; f__; g__; s__ |
| OTU_1035 | k__Bacteria; p__Proteobacteria; c__Gammaproteobacteria; o__Burkholderiales; f__Comamonadaceae; g__Ramlibacter; s__ |
| OTU_1039 | k__Bacteria; p__Chloroflexi |
| OTU_1040 | k__Bacteria; p__Proteobacteria; c__Gammaproteobacteria; o__Gammaproteobacteria_Incertae_Sedis; f__Unknown_Family; g__Acidibacter; s__ |
| OTU_1047 | k__Bacteria; p__Proteobacteria; c__Alphaproteobacteria; o__Elsterales; f__; g__; s__ |
| OTU_1049 | k__Bacteria; p__Actinobacteriota; c__Actinobacteria; o__Corynebacteriales; f__Nocardiaceae; g__Smaragdicoccus; s__ |
| OTU_1050 | k__Bacteria; p__Proteobacteria; c__Alphaproteobacteria; o__Caulobacterales; f__Hyphomonadaceae; g__Hirschia; s__ |
| OTU_1053 | k__Bacteria; p__Proteobacteria; c__Alphaproteobacteria; o__Caulobacterales; f__Caulobacteraceae; g__; s__ |
| OTU_1055 | k__Bacteria; p__Gemmatimonadota; c__Gemmatimonadetes; o__Gemmatimonadales; f__Gemmatimonadaceae |
| OTU_1060 | k__Bacteria; p__Proteobacteria; c__Gammaproteobacteria; o__Burkholderiales; f__Comamonadaceae; g__Ramlibacter |
| OTU_1067 | k__Bacteria; p__Actinobacteriota; c__Thermoleophilia; o__Solirubrobacterales; f__67-14; g__67-14; s__ |
| OTU_1068 | k__Bacteria; p__Chloroflexi; c__KD4-96; o__KD4-96; f__KD4-96; g__KD4-96; s__ |
| OTU_1074 | k__Bacteria; p__Myxococcota; c__Polyangia; o__Polyangiales; f__Phaselicystidaceae; g__Phaselicystis; s__ |
| OTU_1079 | k__Bacteria; p__Proteobacteria; c__Alphaproteobacteria; o__Rhizobiales |
| OTU_1084 | k__Bacteria; p__Proteobacteria; c__Alphaproteobacteria; o__Sphingomonadales; f__Sphingomonadaceae; g__Novosphingobium; s__ |
| OTU_1088 | k__Bacteria; p__Proteobacteria; c__Alphaproteobacteria; o__Micropepsales; f__Micropepsaceae |
| OTU_1089 | k__Bacteria; p__Chloroflexi; c__Ktedonobacteria; o__C0119; f__C0119; g__C0119; s__ |
| OTU_1092 | k__Bacteria; p__Proteobacteria; c__Gammaproteobacteria; o__Pseudomonadales; f__Pseudomonadaceae; g__Pseudomonas |
| OTU_1094 | k__Bacteria; p__Acidobacteriota; c__Acidobacteriae |
| OTU_1100 | k__Bacteria; p__Acidobacteriota; c__Acidobacteriae; o__Bryobacterales; f__Bryobacteraceae; g__Bryobacter; s__ |
| OTU_1102 | k__Bacteria; p__Verrucomicrobiota; c__Chlamydiae; o__Chlamydiales |
| OTU_1106 | k__Bacteria; p__Actinobacteriota; c__Actinobacteria; o__Corynebacteriales; f__Mycobacteriaceae; g__Mycobacterium |
| OTU_1109 | k__Bacteria; p__Proteobacteria; c__Gammaproteobacteria; o__Burkholderiales; f__A21b; g__A21b; s__ |
| OTU_1111 | k__Bacteria; p__Chloroflexi; c__Dehalococcoidia; o__S085; f__S085; g__S085; s__ |
| OTU_1113 | k__Bacteria; p__Chloroflexi; c__Ktedonobacteria; o__Ktedonobacterales; f__Ktedonobacteraceae; g__G12-WMSP1; s__ |
| OTU_1114 | k__Bacteria; p__Chloroflexi; c__Ktedonobacteria; o__Ktedonobacterales; f__JG30-KF-AS9; g__JG30-KF-AS9 |
| OTU_1118 | k__Bacteria; p__MBNT15; c__MBNT15; o__MBNT15; f__MBNT15; g__MBNT15 |
| OTU_1119 | k__Bacteria; p__Actinobacteriota; c__Thermoleophilia; o__Solirubrobacterales; f__67-14; g__67-14; s__ |
| OTU_1123 | k__Bacteria; p__Proteobacteria; c__Alphaproteobacteria; o__Rhizobiales; f__Rhodomicrobiaceae; g__Rhodomicrobium; s__ |
| OTU_1127 | k__Bacteria; p__Actinobacteriota; c__Actinobacteria; o__Propionibacteriales; f__Nocardioidaceae; g__Actinopolymorpha |
| OTU_1129 | k__Bacteria; p__Proteobacteria; c__Alphaproteobacteria; o__Rhodospirillales; f__Rhodospirillaceae; g__; s__ |
| OTU_1130 | k__Bacteria; p__Myxococcota; c__Polyangia |
| OTU_1136 | k__Bacteria; p__Proteobacteria; c__Gammaproteobacteria; o__Burkholderiales; f__Comamonadaceae; g__Rhizobacter |
| OTU_1141 | k__Bacteria; p__Planctomycetota; c__Planctomycetes; o__Gemmatales; f__Gemmataceae; g__; s__ |
| OTU_1144 | k__Bacteria; p__Actinobacteriota; c__Actinobacteria; o__Micromonosporales; f__Micromonosporaceae; g__Luedemannella; s__ |
| OTU_1145 | k__Bacteria; p__Actinobacteriota; c__Thermoleophilia; o__Solirubrobacterales; f__67-14; g__67-14; s__ |
| OTU_1147 | k__Bacteria; p__Actinobacteriota; c__Thermoleophilia; o__Gaiellales; f__; g__ |
| OTU_1148 | k__Bacteria; p__Actinobacteriota; c__Acidimicrobiia; o__IMCC26256; f__IMCC26256; g__IMCC26256; s__ |
| OTU_1150 | k__Bacteria; p__Actinobacteriota; c__Actinobacteria; o__Frankiales; f__Acidothermaceae; g__Acidothermus; s__ |
| OTU_1152 | k__Bacteria; p__Actinobacteriota; c__Acidimicrobiia; o__Microtrichales; f__Microtrichaceae; g__; s__ |
| OTU_1160 | k__Bacteria; p__Actinobacteriota; c__Thermoleophilia; o__Gaiellales; f__; g__; s__ |
| OTU_1161 | k__Bacteria; p__Actinobacteriota; c__MB-A2-108; o__MB-A2-108; f__MB-A2-108; g__MB-A2-108; s__ |
| OTU_1163 | k__Bacteria; p__Actinobacteriota; c__Actinobacteria; o__Corynebacteriales; f__Mycobacteriaceae; g__Mycobacterium |
| OTU_1164 | k__Bacteria; p__Verrucomicrobiota; c__Verrucomicrobiae; o__Chthoniobacterales; f__Chthoniobacteraceae; g__Candidatus_Udaeobacter; s__ |
| OTU_1168 | k__Bacteria; p__Proteobacteria; c__Gammaproteobacteria; o__Salinisphaerales; f__Solimonadaceae |
| OTU_1170 | k__Bacteria; p__Gemmatimonadota; c__Gemmatimonadetes; o__Gemmatimonadales; f__Gemmatimonadaceae; g__; s__ |
| OTU_1174 | k__Bacteria; p__Actinobacteriota; c__Thermoleophilia; o__Gaiellales; f__Gaiellaceae; g__Gaiella; s__ |
| OTU_1176 | k__Bacteria; p__Acidobacteriota; c__Subgroup_5; o__Subgroup_5; f__Subgroup_5; g__Subgroup_5; s__ |
| OTU_1177 | k__Bacteria; p__Acidobacteriota; c__Acidobacteriae; o__Bryobacterales; f__Bryobacteraceae; g__Bryobacter; s__ |
| OTU_1180 | k__Bacteria; p__Gemmatimonadota; c__Gemmatimonadetes; o__Gemmatimonadales; f__Gemmatimonadaceae; g__Gemmatimonas; s__ |
| OTU_1181 | k__Bacteria; p__Firmicutes; c__Bacilli; o__Bacillales; f__Bacillaceae; g__Geobacillus |
| OTU_1182 | k__Bacteria; p__Verrucomicrobiota; c__Verrucomicrobiae; o__Pedosphaerales; f__Pedosphaeraceae; g__Pedosphaeraceae; s__ |
| OTU_1185 | k__Bacteria; p__Actinobacteriota; c__MB-A2-108; o__MB-A2-108; f__MB-A2-108; g__MB-A2-108; s__ |
| OTU_1188 | k__Bacteria; p__Bacteroidota; c__Kapabacteria; o__Kapabacteriales; f__Kapabacteriales; g__Kapabacteriales; s__ |
| OTU_1198 | k__Bacteria; p__Proteobacteria; c__Alphaproteobacteria; o__Acetobacterales; f__Acetobacteraceae |
| OTU_1199 | k__Bacteria; p__Proteobacteria; c__Gammaproteobacteria; o__Burkholderiales; f__TRA3-20; g__TRA3-20; s__ |
| OTU_1200 | k__Bacteria; p__Acidobacteriota; c__Subgroup_22; o__Subgroup_22; f__Subgroup_22; g__Subgroup_22; s__ |
| OTU_1203 | k__Bacteria; p__Verrucomicrobiota; c__Verrucomicrobiae; o__Chthoniobacterales; f__Xiphinematobacteraceae; g__Candidatus_Xiphinematobacter; s__ |
| OTU_1205 | k__Bacteria; p__Proteobacteria; c__Gammaproteobacteria; o__Burkholderiales; f__SC-I-84; g__SC-I-84; s__ |
| OTU_1207 | k__Bacteria; p__Proteobacteria; c__Gammaproteobacteria; o__Burkholderiales; f__Nitrosomonadaceae; g__MND1; s__ |
| OTU_1209 | k__Bacteria; p__Actinobacteriota; c__Acidimicrobiia; o__; f__; g__; s__ |
| OTU_1224 | k__Bacteria; p__Elusimicrobiota; c__Elusimicrobia; o__Lineage_IV; f__Lineage_IV; g__Lineage_IV; s__ |
| OTU_1228 | k__Bacteria; p__Chloroflexi; c__TK10; o__TK10; f__TK10; g__TK10; s__ |
| OTU_1232 | k__Bacteria; p__Actinobacteriota; c__Thermoleophilia; o__Gaiellales; f__; g__; s__ |
| OTU_1233 | k__Bacteria; p__Actinobacteriota; c__Acidimicrobiia |
| OTU_1234 | k__Bacteria; p__Firmicutes; c__Bacilli; o__Paenibacillales; f__Paenibacillaceae; g__Paenibacillus; s__Paenibacillus_alginolyticus |
| OTU_1244 | k__Bacteria; p__Bdellovibrionota; c__Bdellovibrionia; o__Bdellovibrionales; f__Bdellovibrionaceae; g__Bdellovibrio; s__ |
| OTU_1246 | k__Bacteria; p__Acidobacteriota; c__Acidobacteriae; o__Acidobacteriales; f__; g__; s__ |
| OTU_1247 | k__Bacteria; p__Acidobacteriota; c__Subgroup_5; o__Subgroup_5; f__Subgroup_5; g__Subgroup_5; s__ |
| OTU_1252 | k__Bacteria; p__Firmicutes; c__Bacilli; o__Paenibacillales; f__Paenibacillaceae; g__Paenibacillus; s__Paenibacillus_glycanilyticus |
| OTU_1259 | k__Bacteria; p__Acidobacteriota; c__Acidobacteriae; o__Bryobacterales; f__Bryobacteraceae; g__Bryobacter; s__ |
| OTU_1261 | k__Bacteria; p__Proteobacteria; c__Alphaproteobacteria; o__Rhizobiales; f__Xanthobacteraceae; g__; s__ |
| OTU_1262 | k__Bacteria; p__Proteobacteria; c__Gammaproteobacteria |
| OTU_1265 | k__Bacteria; p__Bacteroidota; c__Bacteroidia; o__Chitinophagales; f__Chitinophagaceae; g__Edaphobaculum; s__ |
| OTU_1266 | k__Bacteria; p__Proteobacteria; c__Alphaproteobacteria; o__Rhizobiales; f__Rhizobiales_Incertae_Sedis; g__Bauldia; s__ |
| OTU_1279 | k__Bacteria; p__Bacteroidota; c__Bacteroidia; o__Sphingobacteriales; f__CWT_CU03-E12; g__CWT_CU03-E12; s__ |
| OTU_1280 | k__Bacteria; p__Proteobacteria; c__Gammaproteobacteria; o__Burkholderiales; f__Burkholderiaceae; g__Polynucleobacter; s__Polynucleobacter_asymbioticus |
| OTU_1285 | k__Bacteria; p__Actinobacteriota; c__Actinobacteria; o__Catenulisporales; f__Catenulisporaceae; g__Catenulispora; s__Catenulispora_sp. |
| OTU_1289 | k__Bacteria; p__Actinobacteriota; c__Actinobacteria; o__Pseudonocardiales; f__Pseudonocardiaceae |
| OTU_1291 | k__Bacteria; p__Methylomirabilota; c__Methylomirabilia; o__Rokubacteriales; f__Rokubacteriales; g__Rokubacteriales; s__ |
| OTU_1301 | k__Bacteria; p__Actinobacteriota; c__Acidimicrobiia; o__IMCC26256; f__IMCC26256; g__IMCC26256; s__ |
| OTU_1302 | k__Bacteria; p__Myxococcota; c__Myxococcia; o__Myxococcales |
| OTU_1304 | k__Bacteria; p__Chloroflexi; c__Ktedonobacteria; o__Ktedonobacterales; f__Ktedonobacteraceae |
| OTU_1307 | k__Bacteria; p__Chloroflexi; c__JG30-KF-CM66; o__JG30-KF-CM66; f__JG30-KF-CM66; g__JG30-KF-CM66; s__ |
| OTU_1308 | k__Bacteria; p__Actinobacteriota; c__Acidimicrobiia; o__Microtrichales |
| OTU_1309 | k__Bacteria; p__Planctomycetota; c__Planctomycetes; o__Isosphaerales; f__Isosphaeraceae; g__Aquisphaera; s__ |
| OTU_1315 | k__Bacteria |
| OTU_1319 | k__Bacteria; p__Actinobacteriota; c__Acidimicrobiia; o__Microtrichales |
| OTU_1335 | k__Bacteria; p__Chloroflexi; c__JG30-KF-CM66; o__JG30-KF-CM66; f__JG30-KF-CM66; g__JG30-KF-CM66; s__ |
| OTU_1344 | k__Bacteria; p__Proteobacteria; c__Gammaproteobacteria; o__KF-JG30-C25; f__KF-JG30-C25; g__KF-JG30-C25; s__ |
| OTU_1348 | k__Bacteria; p__Actinobacteriota; c__Actinobacteria; o__Corynebacteriales; f__Nocardiaceae; g__Nocardia |
| OTU_1352 | k__Bacteria; p__Firmicutes; c__Bacilli; o__Bacillales; f__Bacillaceae; g__Bacillus |
| OTU_1353 | k__Bacteria; p__Actinobacteriota; c__Thermoleophilia; o__Gaiellales; f__; g__; s__ |
| OTU_1361 | k__Bacteria; p__Latescibacterota; c__Latescibacterota; o__Latescibacterota; f__Latescibacterota; g__Latescibacterota; s__ |
| OTU_1384 | k__Bacteria; p__Acidobacteriota; c__Acidobacteriae; o__Subgroup_2; f__Subgroup_2; g__Subgroup_2; s__ |
| OTU_1389 | k__Bacteria; p__Bdellovibrionota; c__Oligoflexia; o__0319-6G20; f__0319-6G20; g__0319-6G20; s__ |
| OTU_1393 | k__Bacteria; p__Acidobacteriota; c__Subgroup_22; o__Subgroup_22; f__Subgroup_22; g__Subgroup_22; s__ |
| OTU_1400 | k__Bacteria; p__Acidobacteriota; c__Acidobacteriae; o__Subgroup_13; f__Subgroup_13; g__Subgroup_13; s__ |
| OTU_1405 | k__Bacteria; p__Bacteroidota; c__Bacteroidia; o__Chitinophagales; f__Chitinophagaceae; g__Puia; s__ |
| OTU_1409 | k__Bacteria; p__Proteobacteria; c__Alphaproteobacteria; o__Rhizobiales; f__Beijerinckiaceae; g__Microvirga; s__ |
| OTU_1412 | k__Bacteria; p__Actinobacteriota; c__Actinobacteria |
| OTU_1417 | k__Bacteria; p__Proteobacteria; c__Gammaproteobacteria; o__Gammaproteobacteria_Incertae_Sedis; f__Unknown_Family; g__Acidibacter; s__ |
| OTU_1418 | k__Bacteria; p__Chloroflexi; c__TK10; o__TK10; f__TK10; g__TK10 |
| OTU_1420 | k__Bacteria; p__Actinobacteriota; c__Actinobacteria; o__Corynebacteriales; f__Nocardiaceae; g__Rhodococcus |
| OTU_1422 | k__Bacteria; p__Acidobacteriota; c__Acidobacteriae; o__PAUC26f; f__PAUC26f; g__PAUC26f; s__ |
| OTU_1429 | k__Bacteria; p__Proteobacteria; c__Gammaproteobacteria; o__Burkholderiales; f__Burkholderiaceae; g__Cupriavidus |
| OTU_1445 | k__Bacteria; p__Actinobacteriota; c__Thermoleophilia; o__Gaiellales; f__; g__ |
| OTU_1449 | k__Bacteria; p__Acidobacteriota; c__Vicinamibacteria; o__Vicinamibacterales; f__; g__; s__ |
| OTU_1459 | k__Bacteria; p__Myxococcota; c__Polyangia; o__Haliangiales; f__Haliangiaceae; g__Haliangium; s__ |
| OTU_1461 | k__Bacteria; p__Acidobacteriota; c__Subgroup_11; o__Subgroup_11; f__Subgroup_11; g__Subgroup_11; s__ |
| OTU_1472 | k__Bacteria; p__Actinobacteriota; c__Thermoleophilia; o__Solirubrobacterales; f__Solirubrobacteraceae; g__Conexibacter; s__ |
| OTU_1473 | k__Bacteria; p__Acidobacteriota; c__Acidobacteriae; o__Solibacterales; f__Solibacteraceae; g__Candidatus_Solibacter; s__ |
| OTU_1479 | k__Bacteria; p__Actinobacteriota; c__Thermoleophilia; o__Gaiellales; f__; g__; s__ |
| OTU_1491 | k__Bacteria; p__Acidobacteriota; c__Acidobacteriae; o__Solibacterales; f__Solibacteraceae; g__Candidatus_Solibacter; s__ |
| OTU_1493 | k__Bacteria; p__Proteobacteria; c__Alphaproteobacteria; o__Acetobacterales; f__Acetobacteraceae; g__; s__ |
| OTU_1497 | k__Bacteria; p__Proteobacteria; c__Gammaproteobacteria; o__Burkholderiales; f__TRA3-20; g__TRA3-20; s__ |
| OTU_1504 | k__Bacteria; p__Cyanobacteria; c__Sericytochromatia; o__Sericytochromatia; f__Sericytochromatia; g__Sericytochromatia; s__ |
| OTU_1506 | k__Bacteria; p__Chloroflexi; c__Anaerolineae; o__Anaerolineales; f__Anaerolineaceae; g__; s__ |
| OTU_1513 | k__Bacteria; p__Proteobacteria; c__Gammaproteobacteria; o__Gammaproteobacteria_Incertae_Sedis; f__Unknown_Family; g__Acidibacter |
| OTU_1518 | k__Bacteria; p__Acidobacteriota; c__Acidobacteriae; o__Solibacterales; f__Solibacteraceae; g__Candidatus_Solibacter; s__ |
| OTU_1521 | k__Bacteria; p__Verrucomicrobiota; c__Verrucomicrobiae; o__Opitutales; f__Opitutaceae |
| OTU_1532 | k__Bacteria; p__Gemmatimonadota; c__Gemmatimonadetes; o__Gemmatimonadales; f__Gemmatimonadaceae; g__; s__Acidobacteria_bacterium |
| OTU_1538 | k__Bacteria; p__Actinobacteriota; c__Actinobacteria; o__Kineosporiales; f__Kineosporiaceae |
| OTU_1543 | k__Bacteria; p__Acidobacteriota; c__Subgroup_5; o__Subgroup_5; f__Subgroup_5; g__Subgroup_5; s__ |
| OTU_1549 | k__Bacteria; p__Actinobacteriota; c__Actinobacteria; o__Propionibacteriales; f__Nocardioidaceae; g__Nocardioides |
| OTU_1552 | k__Bacteria; p__Proteobacteria; c__Gammaproteobacteria; o__Burkholderiales; f__A21b; g__A21b; s__ |
| OTU_1555 | k__Bacteria; p__Actinobacteriota; c__Actinobacteria; o__Frankiales; f__Geodermatophilaceae; g__Blastococcus; s__ |
| OTU_1560 | k__Bacteria; p__Acidobacteriota; c__Acidobacteriae |
| OTU_1573 | k__Bacteria; p__Acidobacteriota; c__Acidobacteriae; o__Bryobacterales; f__Bryobacteraceae; g__Bryobacter; s__ |
| OTU_1579 | k__Bacteria; p__Proteobacteria; c__Gammaproteobacteria; o__Burkholderiales; f__Nitrosomonadaceae; g__MND1; s__ |
| OTU_1587 | k__Bacteria; p__Chloroflexi; c__TK10; o__TK10; f__TK10; g__TK10; s__ |
| OTU_1588 | k__Bacteria; p__Actinobacteriota; c__Acidimicrobiia; o__; f__; g__; s__ |
| OTU_1589 | k__Bacteria; p__Chloroflexi; c__Anaerolineae; o__Anaerolineales; f__Anaerolineaceae; g__; s__ |
| OTU_1592 | k__Bacteria; p__Bacteroidota; c__Bacteroidia; o__Chitinophagales; f__Chitinophagaceae; g__Edaphobaculum; s__ |
| OTU_1593 | k__Bacteria; p__Proteobacteria; c__Alphaproteobacteria; o__Micropepsales; f__Micropepsaceae; g__; s__ |
| OTU_1596 | k__Bacteria; p__Proteobacteria; c__Gammaproteobacteria; o__Burkholderiales; f__A21b; g__A21b; s__ |
| OTU_1614 | k__Bacteria; p__Verrucomicrobiota; c__Verrucomicrobiae; o__Chthoniobacterales; f__Xiphinematobacteraceae; g__Candidatus_Xiphinematobacter; s__ |
| OTU_1615 | k__Bacteria; p__Gemmatimonadota; c__Gemmatimonadetes; o__Gemmatimonadales; f__Gemmatimonadaceae |
| OTU_1616 | k__Bacteria; p__Firmicutes; c__Bacilli; o__Alicyclobacillales; f__Alicyclobacillaceae; g__Tumebacillus; s__Tumebacillus_permanentifrigoris |
| OTU_1624 | k__Bacteria; p__Proteobacteria; c__Alphaproteobacteria; o__Acetobacterales; f__Acetobacteraceae; g__Acidiphilium; s__ |
| OTU_1626 | k__Bacteria; p__Actinobacteriota; c__Thermoleophilia; o__Solirubrobacterales; f__67-14; g__67-14; s__ |
| OTU_1631 | k__Bacteria; p__Actinobacteriota; c__Thermoleophilia; o__Solirubrobacterales; f__67-14; g__67-14; s__ |
| OTU_1640 | k__Bacteria; p__Bdellovibrionota; c__Bdellovibrionia; o__Bdellovibrionales; f__Bdellovibrionaceae; g__Bdellovibrio; s__ |
| OTU_1644 | k__Bacteria; p__Proteobacteria; c__Alphaproteobacteria; o__Rhizobiales; f__Xanthobacteraceae |
| OTU_1646 | k__Bacteria; p__Actinobacteriota; c__Thermoleophilia; o__Gaiellales; f__; g__; s__ |
| OTU_1650 | k__Bacteria; p__Proteobacteria; c__Alphaproteobacteria; o__Sphingomonadales; f__Sphingomonadaceae |
| OTU_1651 | k__Bacteria |
| OTU_1657 | k__Bacteria; p__Verrucomicrobiota; c__Verrucomicrobiae; o__Pedosphaerales; f__Pedosphaeraceae; g__; s__ |
| OTU_1663 | k__Bacteria; p__Acidobacteriota; c__Acidobacteriae; o__Acidobacteriales; f__; g__; s__ |
| OTU_1668 | k__Bacteria; p__Myxococcota; c__Polyangia; o__Haliangiales; f__Haliangiaceae; g__Haliangium; s__ |
| OTU_1670 | k__Bacteria; p__Bacteroidota; c__Bacteroidia; o__Sphingobacteriales; f__CWT_CU03-E12; g__CWT_CU03-E12; s__ |
| OTU_1674 | k__Bacteria; p__Proteobacteria; c__Gammaproteobacteria; o__Burkholderiales; f__Nitrosomonadaceae; g__MND1; s__ |
| OTU_1685 | k__Bacteria; p__Chloroflexi; c__Ktedonobacteria; o__Ktedonobacterales; f__JG30-KF-AS9; g__JG30-KF-AS9 |
| OTU_1696 | k__Bacteria; p__Myxococcota; c__Polyangia; o__Haliangiales; f__Haliangiaceae; g__Haliangium; s__ |
| OTU_1701 | k__Bacteria; p__Actinobacteriota; c__MB-A2-108; o__MB-A2-108; f__MB-A2-108; g__MB-A2-108; s__ |
| OTU_1720 | k__Bacteria; p__Proteobacteria; c__Alphaproteobacteria; o__Rhizobiales; f__Xanthobacteraceae; g__Afipia; s__ |
| OTU_1724 | k__Bacteria; p__Actinobacteriota; c__Actinobacteria; o__Frankiales; f__Sporichthyaceae; g__Sporichthya; s__Sporichthya_polymorpha |
| OTU_1734 | k__Bacteria; p__Verrucomicrobiota; c__Verrucomicrobiae; o__Pedosphaerales; f__Pedosphaeraceae; g__ADurb.Bin063-1; s__ |
| OTU_1741 | k__Bacteria; p__Proteobacteria; c__Alphaproteobacteria; o__Elsterales; f__; g__; s__ |
| OTU_1748 | k__Bacteria; p__Acidobacteriota; c__Acidobacteriae; o__Acidobacteriales; f__Acidobacteriaceae_(Subgroup_1); g__; s__ |
| OTU_1751 | k__Bacteria; p__Acidobacteriota; c__Vicinamibacteria; o__Vicinamibacterales; f__; g__; s__ |
| OTU_1761 | k__Bacteria; p__Elusimicrobiota; c__Lineage_IIa; o__Lineage_IIa; f__Lineage_IIa; g__Lineage_IIa; s__ |
| OTU_1762 | k__Bacteria; p__Proteobacteria; c__Alphaproteobacteria |
| OTU_1768 | k__Bacteria; p__Proteobacteria; c__Alphaproteobacteria; o__Elsterales; f__; g__; s__ |
| OTU_1782 | k__Bacteria; p__Firmicutes; c__Bacilli; o__Bacillales; f__Planococcaceae; g__Solibacillus |
| OTU_1788 | k__Bacteria; p__Myxococcota; c__Polyangia |
| OTU_1796 | k__Bacteria; p__Proteobacteria; c__Alphaproteobacteria; o__Rickettsiales; f__SM2D12; g__SM2D12; s__ |
| OTU_1814 | k__Bacteria; p__Actinobacteriota; c__Acidimicrobiia; o__IMCC26256; f__IMCC26256; g__IMCC26256; s__bacterium_enrichment |
| OTU_1827 | k__Bacteria; p__Proteobacteria; c__Alphaproteobacteria; o__Elsterales; f__; g__; s__ |
| OTU_1834 | k__Bacteria; p__Proteobacteria; c__Alphaproteobacteria; o__Rhizobiales; f__Xanthobacteraceae; g__Rhodoplanes; s__ |
| OTU_1835 | k__Bacteria; p__Acidobacteriota; c__Subgroup_22; o__Subgroup_22; f__Subgroup_22; g__Subgroup_22; s__ |
| OTU_1837 | k__Bacteria; p__Acidobacteriota; c__Acidobacteriae; o__Subgroup_2; f__Subgroup_2; g__Subgroup_2; s__ |
| OTU_1839 | k__Bacteria; p__Actinobacteriota; c__Actinobacteria; o__Frankiales; f__Frankiaceae; g__Jatrophihabitans |
| OTU_1852 | k__Bacteria; p__Proteobacteria; c__Alphaproteobacteria; o__Sphingomonadales; f__Sphingomonadaceae |
| OTU_1858 | k__Bacteria; p__Actinobacteriota; c__Thermoleophilia; o__Solirubrobacterales; f__Solirubrobacteraceae; g__Conexibacter; s__ |
| OTU_1859 | k__Bacteria; p__SAR324_clade(Marine_group_B); c__SAR324_clade(Marine_group_B); o__SAR324_clade(Marine_group_B); f__SAR324_clade(Marine_group_B); g__SAR324_clade(Marine_group_B); s__bacterium_enrichment |
| OTU_1865 | k__Bacteria; p__Acidobacteriota; c__Acidobacteriae; o__Subgroup_13; f__Subgroup_13; g__Subgroup_13; s__ |
| OTU_1882 | k__Bacteria; p__Proteobacteria; c__Alphaproteobacteria; o__Rhizobiales; f__Hyphomicrobiaceae; g__Hyphomicrobium; s__ |
| OTU_1887 | k__Bacteria; p__Chloroflexi; c__Anaerolineae; o__SBR1031; f__A4b; g__A4b; s__ |
| OTU_1898 | k__Bacteria; p__Planctomycetota; c__Planctomycetes; o__Isosphaerales; f__Isosphaeraceae; g__Tundrisphaera; s__ |
| OTU_1904 | k__Bacteria; p__Acidobacteriota; c__Subgroup_5; o__Subgroup_5; f__Subgroup_5; g__Subgroup_5; s__ |
| OTU_1927 | k__Bacteria; p__Actinobacteriota; c__Thermoleophilia; o__Solirubrobacterales; f__Solirubrobacteraceae; g__Conexibacter |
| OTU_1929 | k__Bacteria; p__Firmicutes; c__Bacilli; o__Paenibacillales; f__Paenibacillaceae; g__Paenibacillus; s__Paenibacillus_sacheonensis |
| OTU_1939 | k__Bacteria; p__Acidobacteriota; c__Holophagae; o__Subgroup_7; f__Subgroup_7; g__Subgroup_7; s__ |
| OTU_1951 | k__Bacteria; p__Latescibacterota; c__Latescibacterota; o__Latescibacterota; f__Latescibacterota; g__Latescibacterota; s__ |
| OTU_1952 | k__Bacteria; p__Proteobacteria; c__Alphaproteobacteria; o__Sphingomonadales; f__Sphingomonadaceae; g__Novosphingobium; s__Novosphingobium_capsulatum |
| OTU_1976 | k__Bacteria; p__Proteobacteria; c__Gammaproteobacteria; o__Burkholderiales; f__Comamonadaceae |
| OTU_1979 | k__Bacteria; p__Proteobacteria; c__Gammaproteobacteria; o__Burkholderiales; f__SC-I-84; g__SC-I-84; s__ |
| OTU_1983 | k__Bacteria; p__Verrucomicrobiota; c__Verrucomicrobiae; o__Pedosphaerales; f__Pedosphaeraceae; g__; s__ |
| OTU_1989 | k__Bacteria; p__Chloroflexi; c__Ktedonobacteria; o__Ktedonobacterales; f__Ktedonobacteraceae; g__HSB_OF53-F07; s__ |
| OTU_1994 | k__Bacteria; p__Acidobacteriota; c__Acidobacteriae; o__Bryobacterales; f__Bryobacteraceae; g__Bryobacter; s__ |
| OTU_1997 | k__Bacteria; p__Planctomycetota; c__Planctomycetes; o__Isosphaerales; f__Isosphaeraceae |
| OTU_2011 | k__Bacteria; p__Actinobacteriota; c__Actinobacteria; o__Frankiales; f__Acidothermaceae; g__Acidothermus; s__ |
| OTU_2019 | k__Bacteria; p__Verrucomicrobiota; c__Verrucomicrobiae; o__Pedosphaerales; f__Pedosphaeraceae; g__ |
| OTU_2042 | k__Bacteria; p__Proteobacteria; c__Alphaproteobacteria; o__Azospirillales; f__Inquilinaceae; g__Inquilinus; s__ |
| OTU_2043 | k__Bacteria; p__Chloroflexi; c__Ktedonobacteria; o__Ktedonobacterales; f__Ktedonobacteraceae; g__1921-3; s__ |
| OTU_2056 | k__Bacteria; p__Chloroflexi; c__TK10; o__TK10; f__TK10; g__TK10; s__ |
| OTU_2066 | k__Bacteria; p__Proteobacteria; c__Alphaproteobacteria; o__Rhizobiales; f__Beijerinckiaceae; g__Psychroglaciecola; s__ |
| OTU_2069 | k__Bacteria; p__Firmicutes; c__Bacilli; o__Paenibacillales; f__Paenibacillaceae; g__Cohnella |
| OTU_2086 | k__Bacteria; p__Proteobacteria; c__Alphaproteobacteria; o__Rhizobiales; f__Xanthobacteraceae |
| OTU_2107 | k__Bacteria; p__Chloroflexi; c__TK10; o__TK10; f__TK10; g__TK10; s__bacterium_Ellin6519 |
| OTU_2157 | k__Bacteria; p__Proteobacteria; c__Alphaproteobacteria; o__Rhizobiales; f__Xanthobacteraceae |
| OTU_2158 | k__Bacteria; p__Proteobacteria; c__Alphaproteobacteria; o__Acetobacterales; f__Acetobacteraceae; g__; s__ |
| OTU_2161 | k__Bacteria; p__Proteobacteria; c__Alphaproteobacteria; o__Elsterales; f__; g__; s__ |
| OTU_2163 | k__Bacteria; p__Actinobacteriota; c__Thermoleophilia; o__Solirubrobacterales |
| OTU_2175 | k__Bacteria; p__Planctomycetota; c__Planctomycetes; o__Gemmatales; f__Gemmataceae; g__; s__ |
| OTU_2177 | k__Bacteria; p__Chloroflexi; c__Ktedonobacteria; o__Ktedonobacterales; f__Ktedonobacteraceae |
| OTU_2187 | k__Bacteria; p__Proteobacteria; c__Alphaproteobacteria; o__Rhizobiales; f__Rhizobiales_Incertae_Sedis; g__Bauldia; s__ |
| OTU_2190 | k__Bacteria; p__Planctomycetota; c__Planctomycetes; o__Gemmatales; f__Gemmataceae; g__; s__ |
| OTU_2216 | k__Bacteria; p__Acidobacteriota; c__Acidobacteriae; o__Acidobacteriales; f__Acidobacteriaceae_(Subgroup_1); g__Bryocella; s__ |
| OTU_2224 | k__Bacteria; p__Acidobacteriota; c__Acidobacteriae; o__Bryobacterales; f__Bryobacteraceae; g__Bryobacter; s__ |
| OTU_2233 | k__Bacteria; p__Myxococcota; c__Polyangia; o__Haliangiales; f__Haliangiaceae; g__Haliangium; s__ |
| OTU_2235 | k__Bacteria; p__Proteobacteria; c__Alphaproteobacteria; o__Paracaedibacterales; f__Paracaedibacteraceae; g__Candidatus_Paracaedibacter; s__ |
| OTU_2245 | k__Bacteria; p__Methylomirabilota; c__Methylomirabilia; o__Rokubacteriales; f__Rokubacteriales; g__Rokubacteriales; s__ |
| OTU_2248 | k__Bacteria; p__Planctomycetota; c__Planctomycetes; o__Gemmatales; f__Gemmataceae; g__Gemmata; s__ |
| OTU_2262 | k__Bacteria; p__Verrucomicrobiota; c__Verrucomicrobiae; o__Opitutales; f__Opitutaceae; g__Opitutus; s__ |
| OTU_2271 | k__Bacteria; p__Acidobacteriota; c__Acidobacteriae; o__Subgroup_2; f__Subgroup_2; g__Subgroup_2; s__ |
| OTU_2280 | k__Bacteria; p__Myxococcota; c__Myxococcia; o__Myxococcales; f__Myxococcaceae; g__P3OB-42; s__ |
| OTU_2287 | k__Bacteria; p__Actinobacteriota; c__Actinobacteria; o__Frankiales |
| OTU_2312 | k__Bacteria; p__Bacteroidota; c__Bacteroidia; o__Chitinophagales; f__37-13; g__37-13; s__ |
| OTU_2317 | k__Bacteria; p__Proteobacteria; c__Gammaproteobacteria; o__WD260; f__WD260; g__WD260; s__ |
| OTU_2319 | k__Bacteria; p__Proteobacteria; c__Alphaproteobacteria; o__Acetobacterales; f__Acetobacteraceae; g__Acidiphilium |
| OTU_2324 | k__Bacteria; p__Actinobacteriota; c__Acidimicrobiia; o__Microtrichales; f__Ilumatobacteraceae; g__CL500-29_marine_group |
| OTU_2341 | k__Bacteria; p__Proteobacteria; c__Gammaproteobacteria; o__Burkholderiales; f__Comamonadaceae |
| OTU_2356 | k__Bacteria; p__Bacteroidota; c__Bacteroidia; o__Sphingobacteriales; f__Sphingobacteriaceae; g__Mucilaginibacter |
| OTU_2361 | k__Bacteria; p__Myxococcota; c__Myxococcia; o__Myxococcales; f__Myxococcaceae |
| OTU_2385 | k__Bacteria; p__Proteobacteria; c__Gammaproteobacteria; o__Burkholderiales; f__Oxalobacteraceae; g__Duganella; s__Duganella_sp. |
| OTU_2386 | k__Bacteria; p__Acidobacteriota; c__Acidobacteriae; o__Subgroup_2; f__Subgroup_2; g__Subgroup_2; s__ |
| OTU_2395 | k__Bacteria; p__Chloroflexi; c__Chloroflexia; o__Thermomicrobiales; f__JG30-KF-CM45; g__JG30-KF-CM45; s__bacterium_Ellin6537 |
| OTU_2406 | k__Bacteria; p__Acidobacteriota; c__Holophagae; o__Subgroup_7; f__Subgroup_7; g__Subgroup_7; s__ |
| OTU_2407 | k__Bacteria; p__Myxococcota; c__Polyangia; o__Polyangiales; f__Polyangiaceae; g__Pajaroellobacter; s__ |
| OTU_2408 | k__Bacteria; p__Proteobacteria; c__Alphaproteobacteria; o__Elsterales; f__; g__; s__ |
| OTU_2412 | k__Bacteria; p__Proteobacteria; c__Alphaproteobacteria; o__Rhizobiales |
| OTU_2419 | k__Bacteria; p__Firmicutes; c__Bacilli; o__Brevibacillales; f__Brevibacillaceae; g__Brevibacillus |
| OTU_2432 | k__Bacteria; p__Verrucomicrobiota; c__Verrucomicrobiae; o__Pedosphaerales; f__Pedosphaeraceae; g__ADurb.Bin063-1; s__ |
| OTU_2442 | k__Bacteria; p__Nitrospirota; c__Nitrospiria; o__Nitrospirales; f__Nitrospiraceae; g__Nitrospira; s__ |
| OTU_2444 | k__Bacteria; p__Actinobacteriota; c__Acidimicrobiia; o__IMCC26256; f__IMCC26256; g__IMCC26256; s__bacterium_enrichment |
| OTU_2447 | k__Bacteria; p__Armatimonadota; c__; o__; f__; g__; s__ |
| OTU_2457 | k__Bacteria; p__Proteobacteria; c__Alphaproteobacteria; o__Holosporales; f__Holosporaceae; g__; s__ |
| OTU_2465 | k__Bacteria; p__Proteobacteria; c__Alphaproteobacteria; o__Acetobacterales; f__Acetobacteraceae; g__Acidisoma; s__ |
| OTU_2471 | k__Bacteria; p__Myxococcota; c__Polyangia; o__Polyangiales; f__Polyangiaceae |
| OTU_2486 | k__Bacteria; p__Myxococcota; c__Polyangia; o__mle1-27; f__mle1-27; g__mle1-27 |
| OTU_2487 | k__Bacteria; p__Actinobacteriota; c__Actinobacteria; o__Propionibacteriales; f__Nocardioidaceae; g__Nocardioides |
| OTU_2507 | k__Bacteria; p__Planctomycetota; c__Planctomycetes; o__Planctomycetales; f__; g__; s__ |
| OTU_2525 | k__Bacteria; p__Acidobacteriota; c__Acidobacteriae; o__Acidobacteriales; f__Acidobacteriaceae_(Subgroup_1); g__Terriglobus |
| OTU_2534 | k__Bacteria; p__Proteobacteria; c__Alphaproteobacteria; o__Caulobacterales; f__Caulobacteraceae; g__Phenylobacterium |
| OTU_2538 | k__Bacteria; p__Verrucomicrobiota; c__Verrucomicrobiae; o__Chthoniobacterales; f__Chthoniobacteraceae; g__Candidatus_Udaeobacter |
| OTU_2558 | k__Bacteria; p__Myxococcota; c__Polyangia; o__Polyangiales; f__Polyangiaceae; g__Aetherobacter; s__ |
| OTU_2559 | k__Bacteria; p__Proteobacteria; c__Alphaproteobacteria; o__Elsterales; f__; g__; s__ |
| OTU_2561 | k__Bacteria; p__Bacteroidota; c__Bacteroidia; o__Chitinophagales; f__Chitinophagaceae |
| OTU_2589 | k__Bacteria; p__Proteobacteria; c__Gammaproteobacteria; o__Burkholderiales; f__Comamonadaceae |
| OTU_2590 | k__Bacteria; p__Proteobacteria; c__Gammaproteobacteria; o__Burkholderiales; f__SC-I-84; g__SC-I-84; s__ |
| OTU_2595 | k__Bacteria; p__Acidobacteriota; c__Acidobacteriae; o__Solibacterales; f__Solibacteraceae; g__Candidatus_Solibacter; s__ |
| OTU_2622 | k__Bacteria; p__Proteobacteria; c__Alphaproteobacteria |
| OTU_2635 | k__Bacteria; p__Actinobacteriota; c__Thermoleophilia; o__Gaiellales; f__; g__; s__ |
| OTU_2643 | k__Bacteria; p__Acidobacteriota; c__Acidobacteriae; o__Subgroup_2; f__Subgroup_2; g__Subgroup_2; s__ |
| OTU_2654 | k__Bacteria; p__Latescibacterota; c__Latescibacterota; o__Latescibacterota; f__Latescibacterota; g__Latescibacterota; s__ |
| OTU_2670 | k__Bacteria; p__Chloroflexi; c__AD3; o__AD3; f__AD3; g__AD3; s__ |
| OTU_2680 | k__Bacteria; p__Actinobacteriota; c__Actinobacteria; o__Frankiales; f__Acidothermaceae; g__Acidothermus; s__ |
| OTU_2685 | k__Bacteria; p__Acidobacteriota; c__Vicinamibacteria; o__Vicinamibacterales; f__; g__; s__ |
| OTU_2687 | k__Bacteria; p__Patescibacteria; c__Microgenomatia; o__Candidatus_Levybacteria; f__Candidatus_Levybacteria; g__Candidatus_Levybacteria; s__ |
| OTU_2689 | k__Bacteria; p__Acidobacteriota; c__Acidobacteriae; o__Acidobacteriales; f__; g__; s__ |
| OTU_2702 | k__Bacteria; p__Actinobacteriota; c__Acidimicrobiia; o__; f__; g__ |
| OTU_2723 | k__Bacteria; p__Proteobacteria; c__Alphaproteobacteria; o__Rhizobiales; f__Xanthobacteraceae; g__Pseudolabrys; s__ |
| OTU_2725 | k__Bacteria; p__Myxococcota; c__Polyangia; o__Polyangiales; f__Polyangiaceae; g__Pajaroellobacter; s__ |
| OTU_2728 | k__Bacteria; p__Myxococcota; c__Polyangia; o__Polyangiales; f__Polyangiaceae; g__Pajaroellobacter |
| OTU_2745 | k__Bacteria; p__Proteobacteria; c__Alphaproteobacteria; o__Rhizobiales; f__Xanthobacteraceae; g__Rhodoplanes; s__ |
| OTU_2746 | k__Bacteria; p__Chloroflexi; c__TK10; o__TK10; f__TK10; g__TK10; s__bacterium_Ellin6519 |
| OTU_2749 | k__Bacteria; p__MBNT15; c__MBNT15; o__MBNT15; f__MBNT15; g__MBNT15; s__ |
| OTU_2772 | k__Bacteria; p__Acidobacteriota; c__Blastocatellia; o__Blastocatellales; f__Blastocatellaceae; g__JGI_0001001-H03; s__ |
| OTU_2780 | k__Bacteria; p__Actinobacteriota; c__Thermoleophilia; o__Gaiellales; f__; g__; s__ |
| OTU_2786 | k__Bacteria; p__Proteobacteria; c__Alphaproteobacteria; o__; f__; g__; s__ |
| OTU_2805 | k__Bacteria; p__Acidobacteriota; c__Acidobacteriae; o__Bryobacterales; f__Bryobacteraceae; g__Bryobacter; s__ |
| OTU_2810 | k__Bacteria; p__Proteobacteria; c__Alphaproteobacteria; o__Rhodospirillales; f__Rhodospirillaceae; g__; s__ |
| OTU_2814 | k__Bacteria; p__Proteobacteria; c__Alphaproteobacteria; o__Elsterales; f__; g__; s__ |
| OTU_2820 | k__Bacteria; p__Proteobacteria; c__Alphaproteobacteria; o__Rhizobiales; f__Devosiaceae; g__Devosia |
| OTU_2823 | k__Bacteria; p__Elusimicrobiota; c__Lineage_IIa; o__Lineage_IIa; f__Lineage_IIa; g__Lineage_IIa |
| OTU_2843 | k__Bacteria; p__Desulfobacterota; c__; o__; f__; g__; s__ |
| OTU_2848 | k__Bacteria; p__Proteobacteria; c__Alphaproteobacteria; o__Rhizobiales; f__Xanthobacteraceae |
| OTU_2854 | k__Bacteria; p__Actinobacteriota; c__Thermoleophilia; o__Gaiellales |
| OTU_2867 | k__Bacteria; p__Proteobacteria; c__Gammaproteobacteria; o__Burkholderiales; f__Oxalobacteraceae; g__Massilia |
| OTU_2870 | k__Bacteria; p__Proteobacteria; c__Alphaproteobacteria; o__Micropepsales; f__Micropepsaceae |
| OTU_2881 | k__Bacteria; p__Proteobacteria; c__Gammaproteobacteria; o__Burkholderiales; f__Comamonadaceae; g__Polaromonas |
| OTU_2895 | k__Bacteria; p__Actinobacteriota; c__Actinobacteria; o__Propionibacteriales; f__Nocardioidaceae; g__Nocardioides |
| OTU_2903 | k__Bacteria; p__Acidobacteriota; c__Acidobacteriae; o__Bryobacterales; f__Bryobacteraceae; g__Bryobacter; s__ |
| OTU_2916 | k__Bacteria; p__Acidobacteriota; c__Acidobacteriae; o__Acidobacteriales; f__Acidobacteriaceae_(Subgroup_1) |
| OTU_2923 | k__Bacteria; p__Proteobacteria; c__Alphaproteobacteria; o__Rhizobiales; f__Beijerinckiaceae; g__Microvirga; s__Microvirga_sp. |
| OTU_2966 | k__Bacteria; p__Acidobacteriota; c__Acidobacteriae; o__Subgroup_2; f__Subgroup_2; g__Subgroup_2; s__ |
| OTU_2968 | k__Bacteria; p__Firmicutes; c__Bacilli; o__Bacillales; f__Bacillaceae; g__Bacillus |
| OTU_2997 | k__Bacteria; p__Actinobacteriota; c__Actinobacteria; o__Frankiales |
| OTU_3001 | k__Bacteria; p__Actinobacteriota; c__Actinobacteria; o__Frankiales; f__Frankiaceae; g__Jatrophihabitans; s__bacterium_Ellin6090 |
| OTU_3002 | k__Bacteria; p__Proteobacteria; c__Gammaproteobacteria; o__Gammaproteobacteria_Incertae_Sedis; f__Unknown_Family; g__Acidibacter; s__ |
| OTU_3018 | k__Bacteria; p__Actinobacteriota; c__Thermoleophilia; o__Solirubrobacterales; f__67-14; g__67-14; s__ |
| OTU_3027 | k__Bacteria; p__Verrucomicrobiota; c__Verrucomicrobiae; o__Chthoniobacterales; f__Chthoniobacteraceae; g__Chthoniobacter; s__ |
| OTU_3049 | k__Bacteria; p__Proteobacteria; c__Alphaproteobacteria; o__Rhizobiales |
| OTU_3071 | k__Bacteria; p__Proteobacteria; c__Alphaproteobacteria; o__Elsterales; f__; g__; s__ |
| OTU_3091 | k__Bacteria; p__Acidobacteriota; c__Holophagae; o__Subgroup_7; f__Subgroup_7; g__Subgroup_7; s__ |
| OTU_3092 | k__Bacteria; p__Proteobacteria; c__Alphaproteobacteria; o__Rhizobiales; f__Xanthobacteraceae; g__Rhodoplanes; s__ |
| OTU_3110 | k__Bacteria; p__Gemmatimonadota; c__Gemmatimonadetes; o__Gemmatimonadales; f__Gemmatimonadaceae; g__Gemmatimonas; s__ |
| OTU_3127 | k__Bacteria; p__Planctomycetota; c__Planctomycetes; o__Isosphaerales; f__Isosphaeraceae; g__Singulisphaera; s__ |
| OTU_3137 | k__Bacteria; p__Acidobacteriota; c__Acidobacteriae; o__Acidobacteriales |
| OTU_3144 | k__Bacteria; p__Planctomycetota; c__Planctomycetes; o__Gemmatales; f__Gemmataceae; g__; s__ |
| OTU_3145 | k__Bacteria; p__Chloroflexi; c__KD4-96; o__KD4-96; f__KD4-96; g__KD4-96; s__ |
| OTU_3148 | k__Bacteria; p__Actinobacteriota; c__Actinobacteria; o__Micrococcales; f__Microbacteriaceae; g__Leifsonia |
| OTU_3173 | k__Bacteria; p__Verrucomicrobiota; c__Verrucomicrobiae; o__Pedosphaerales; f__Pedosphaeraceae |
| OTU_3189 | k__Bacteria; p__Actinobacteriota; c__Actinobacteria; o__Frankiales; f__Acidothermaceae; g__Acidothermus |
| OTU_3224 | k__Bacteria; p__Actinobacteriota; c__Acidimicrobiia; o__Microtrichales |
| OTU_3234 | k__Bacteria; p__Chloroflexi; c__TK10; o__TK10; f__TK10; g__TK10 |
| OTU_3257 | k__Bacteria; p__Actinobacteriota; c__Thermoleophilia; o__Solirubrobacterales; f__Solirubrobacteraceae; g__Solirubrobacter |
| OTU_3276 | k__Bacteria; p__Actinobacteriota; c__Thermoleophilia; o__Solirubrobacterales; f__Solirubrobacteraceae; g__Conexibacter; s__ |
| OTU_3307 | k__Bacteria; p__Verrucomicrobiota; c__Verrucomicrobiae; o__Pedosphaerales; f__Pedosphaeraceae; g__Pedosphaera; s__ |
| OTU_3315 | k__Bacteria; p__Myxococcota; c__Polyangia; o__Haliangiales; f__Haliangiaceae; g__Haliangium; s__ |
| OTU_3326 | k__Bacteria; p__Actinobacteriota; c__Thermoleophilia; o__Solirubrobacterales; f__Solirubrobacteraceae; g__Conexibacter; s__ |
| OTU_3328 | k__Bacteria; p__Proteobacteria; c__Alphaproteobacteria; o__Micropepsales; f__Micropepsaceae; g__; s__ |
| OTU_3331 | k__Bacteria; p__Proteobacteria; c__Alphaproteobacteria |
| OTU_3376 | k__Bacteria; p__Actinobacteriota; c__Actinobacteria; o__Micromonosporales; f__Micromonosporaceae |
| OTU_3389 | k__Bacteria; p__Proteobacteria; c__Alphaproteobacteria; o__Elsterales; f__; g__; s__ |
| OTU_3437 | k__Bacteria; p__Proteobacteria; c__Alphaproteobacteria; o__Elsterales; f__; g__; s__ |
| OTU_3471 | k__Bacteria; p__Proteobacteria; c__Gammaproteobacteria; o__Legionellales; f__Legionellaceae; g__Legionella; s__Legionella_sp. |
| OTU_3485 | k__Bacteria; p__Myxococcota; c__Myxococcia; o__Myxococcales; f__Anaeromyxobacteraceae; g__Anaeromyxobacter; s__ |
| OTU_3486 | k__Bacteria; p__Verrucomicrobiota; c__Verrucomicrobiae; o__Chthoniobacterales; f__Chthoniobacteraceae; g__Candidatus_Udaeobacter; s__ |
| OTU_3491 | k__Bacteria; p__Proteobacteria; c__Alphaproteobacteria; o__Elsterales; f__; g__; s__ |
| OTU_3496 | k__Bacteria; p__Acidobacteriota; c__Acidobacteriae; o__Acidobacteriales; f__Acidobacteriaceae_(Subgroup_1); g__Granulicella; s__ |
| OTU_3508 | k__Bacteria; p__Proteobacteria; c__Gammaproteobacteria; o__Burkholderiales; f__Comamonadaceae; g__Ramlibacter |
| OTU_3535 | k__Bacteria; p__Actinobacteriota; c__Actinobacteria; o__Streptomycetales; f__Streptomycetaceae; g__Streptomyces |
| OTU_3563 | k__Bacteria; p__Proteobacteria; c__Alphaproteobacteria; o__Elsterales; f__; g__; s__ |
| OTU_3581 | k__Bacteria; p__Acidobacteriota; c__Acidobacteriae; o__Solibacterales; f__Solibacteraceae; g__Candidatus_Solibacter; s__ |
| OTU_3588 | k__Bacteria; p__Proteobacteria; c__Alphaproteobacteria; o__Elsterales; f__; g__; s__ |
| OTU_3608 | k__Bacteria; p__Proteobacteria; c__Gammaproteobacteria; o__Burkholderiales |
| OTU_3616 | k__Bacteria; p__Proteobacteria; c__Gammaproteobacteria; o__Burkholderiales; f__Oxalobacteraceae |
| OTU_3621 | k__Bacteria; p__Actinobacteriota; c__Actinobacteria; o__Frankiales; f__Acidothermaceae; g__Acidothermus; s__ |
| OTU_3623 | k__Bacteria; p__Actinobacteriota; c__Acidimicrobiia; o__IMCC26256; f__IMCC26256; g__IMCC26256; s__ |
| OTU_3629 | k__Bacteria; p__Actinobacteriota; c__Actinobacteria; o__Frankiales; f__Acidothermaceae; g__Acidothermus; s__ |
| OTU_3647 | k__Bacteria; p__Proteobacteria; c__Alphaproteobacteria; o__Rhizobiales |
| OTU_3693 | k__Bacteria; p__Actinobacteriota; c__Thermoleophilia; o__Gaiellales |
| OTU_3697 | k__Bacteria; p__Proteobacteria; c__Alphaproteobacteria; o__Rhizobiales |
| OTU_3702 | k__Bacteria; p__Proteobacteria; c__Alphaproteobacteria; o__Rhizobiales; f__Rhizobiaceae; g__Allorhizobium-Neorhizobium-Pararhizobium-Rhizobium |
| OTU_3707 | k__Bacteria; p__Actinobacteriota; c__Acidimicrobiia; o__IMCC26256; f__IMCC26256; g__IMCC26256; s__ |
| OTU_3717 | k__Bacteria; p__Acidobacteriota; c__Acidobacteriae; o__Subgroup_2; f__Subgroup_2; g__Subgroup_2; s__ |
| OTU_3724 | k__Bacteria; p__Bdellovibrionota; c__Oligoflexia; o__0319-6G20; f__0319-6G20; g__0319-6G20 |
| OTU_3729 | k__Bacteria; p__Actinobacteriota; c__Thermoleophilia; o__Gaiellales |
| OTU_3742 | k__Bacteria; p__Actinobacteriota; c__Actinobacteria; o__Frankiales; f__Frankiaceae; g__Frankia; s__Frankia_sp. |
| OTU_3771 | k__Bacteria; p__Proteobacteria; c__Gammaproteobacteria; o__Burkholderiales; f__Nitrosomonadaceae; g__IS-44; s__ |
| OTU_3787 | k__Bacteria; p__Actinobacteriota; c__Thermoleophilia; o__Solirubrobacterales; f__Solirubrobacteraceae; g__Conexibacter |
| OTU_3910 | k__Bacteria; p__Actinobacteriota; c__Actinobacteria; o__Propionibacteriales; f__Nocardioidaceae; g__Nocardioides |
| OTU_3937 | k__Bacteria; p__Proteobacteria; c__Alphaproteobacteria; o__Rhizobiales; f__Xanthobacteraceae; g__Bradyrhizobium |
| OTU_3943 | k__Bacteria; p__Actinobacteriota; c__Acidimicrobiia |
| OTU_3952 | k__Bacteria; p__Acidobacteriota; c__Acidobacteriae; o__Acidobacteriales |
| OTU_3962 | k__Bacteria; p__Acidobacteriota; c__Acidobacteriae; o__Solibacterales; f__Solibacteraceae; g__Candidatus_Solibacter; s__ |
| OTU_4049 | k__Bacteria; p__Proteobacteria; c__Alphaproteobacteria; o__Rhizobiales; f__Rhizobiales_Incertae_Sedis; g__; s__ |
| OTU_4078 | k__Bacteria; p__Acidobacteriota; c__Vicinamibacteria; o__Vicinamibacterales; f__; g__; s__ |
| OTU_4086 | k__Bacteria; p__Actinobacteriota; c__Thermoleophilia; o__Gaiellales; f__; g__; s__ |
| OTU_4093 | k__Bacteria; p__WPS-2; c__WPS-2; o__WPS-2; f__WPS-2; g__WPS-2; s__ |
| OTU_4162 | k__Bacteria; p__Gemmatimonadota; c__Gemmatimonadetes; o__Gemmatimonadales; f__Gemmatimonadaceae; g__; s__ |
| OTU_4175 | k__Bacteria; p__Actinobacteriota; c__Actinobacteria; o__Propionibacteriales; f__Nocardioidaceae; g__Marmoricola; s__ |
| OTU_4202 | k__Bacteria; p__Actinobacteriota; c__Actinobacteria; o__Pseudonocardiales; f__Pseudonocardiaceae; g__Actinomycetospora; s__Actinomycetospora_sp. |
| OTU_4234 | k__Bacteria; p__Proteobacteria; c__Alphaproteobacteria; o__Micropepsales; f__Micropepsaceae; g__; s__ |
| OTU_4240 | k__Bacteria; p__Proteobacteria; c__Alphaproteobacteria; o__Rickettsiales; f__SM2D12; g__SM2D12; s__ |
| OTU_4250 | k__Bacteria; p__Proteobacteria; c__Alphaproteobacteria; o__Elsterales; f__; g__; s__ |
| OTU_4257 | k__Bacteria; p__Proteobacteria; c__Gammaproteobacteria; o__Burkholderiales; f__Nitrosomonadaceae; g__mle1-7; s__ |
| OTU_4258 | k__Bacteria; p__Acidobacteriota; c__Vicinamibacteria; o__Vicinamibacterales; f__; g__; s__ |
| OTU_4259 | k__Bacteria; p__Bacteroidota; c__Bacteroidia; o__Sphingobacteriales; f__Sphingobacteriaceae; g__Mucilaginibacter; s__ |
| OTU_4263 | k__Bacteria; p__Acidobacteriota; c__Acidobacteriae; o__Subgroup_2; f__Subgroup_2; g__Subgroup_2; s__ |
| OTU_4278 | k__Bacteria; p__Actinobacteriota; c__Acidimicrobiia; o__Microtrichales |
| OTU_4281 | k__Bacteria; p__Planctomycetota; c__Planctomycetes; o__Gemmatales; f__Gemmataceae; g__; s__ |
| OTU_4282 | k__Bacteria; p__Actinobacteriota; c__Thermoleophilia; o__Gaiellales; f__; g__; s__ |
| OTU_4285 | k__Bacteria; p__Acidobacteriota; c__Acidobacteriae; o__Acidobacteriales |
| OTU_4324 | k__Bacteria; p__Acidobacteriota; c__Acidobacteriae; o__Acidobacteriales; f__; g__; s__ |
| OTU_4325 | k__Bacteria; p__Acidobacteriota; c__Holophagae; o__Subgroup_7; f__Subgroup_7; g__Subgroup_7; s__ |
| OTU_4329 | k__Bacteria; p__Acidobacteriota; c__Acidobacteriae; o__Acidobacteriales; f__; g__; s__ |
| OTU_4335 | k__Bacteria; p__Proteobacteria; c__Alphaproteobacteria; o__Rhizobiales; f__Xanthobacteraceae; g__; s__ |
| OTU_4416 | k__Bacteria; p__Desulfobacterota; c__; o__; f__; g__ |
| OTU_4422 | k__Bacteria; p__Proteobacteria; c__Alphaproteobacteria; o__Reyranellales; f__Reyranellaceae; g__Reyranella |
| OTU_4427 | k__Bacteria; p__Proteobacteria; c__Gammaproteobacteria; o__Burkholderiales; f__SC-I-84; g__SC-I-84; s__ |
| OTU_4444 | k__Bacteria; p__Proteobacteria; c__Alphaproteobacteria; o__Elsterales; f__; g__; s__ |
| OTU_4459 | k__Bacteria; p__Actinobacteriota; c__Thermoleophilia; o__Gaiellales |
| OTU_4495 | k__Bacteria; p__Actinobacteriota; c__Acidimicrobiia; o__Microtrichales; f__Ilumatobacteraceae; g__CL500-29_marine_group; s__ |
| OTU_4498 | k__Bacteria; p__Acidobacteriota; c__Acidobacteriae; o__Subgroup_2; f__Subgroup_2; g__Subgroup_2; s__ |
| OTU_4509 | k__Bacteria; p__Proteobacteria; c__Gammaproteobacteria; o__Gammaproteobacteria_Incertae_Sedis; f__Unknown_Family; g__Acidibacter; s__ |
| OTU_4535 | k__Bacteria; p__Bacteroidota; c__Bacteroidia; o__Cytophagales; f__Microscillaceae; g__; s__ |
| OTU_4546 | k__Bacteria; p__Acidobacteriota; c__Acidobacteriae; o__Bryobacterales; f__Bryobacteraceae; g__Bryobacter; s__ |
| OTU_4563 | k__Bacteria; p__Proteobacteria; c__Alphaproteobacteria; o__Elsterales; f__; g__; s__ |
| OTU_4564 | k__Bacteria; p__Actinobacteriota; c__Actinobacteria; o__Streptosporangiales; f__Thermomonosporaceae; g__Actinomadura; s__Actinomadura_sp. |
| OTU_4596 | k__Bacteria; p__Methylomirabilota; c__Methylomirabilia; o__Rokubacteriales; f__Rokubacteriales; g__Rokubacteriales; s__ |
| OTU_4620 | k__Bacteria; p__Bacteroidota; c__Bacteroidia; o__Chitinophagales; f__Chitinophagaceae; g__Puia; s__ |
| OTU_4654 | k__Bacteria; p__Acidobacteriota; c__Acidobacteriae; o__Bryobacterales; f__Bryobacteraceae; g__Bryobacter; s__ |
| OTU_4669 | k__Bacteria; p__Acidobacteriota; c__Acidobacteriae; o__Acidobacteriales; f__; g__; s__ |
| OTU_4680 | k__Bacteria; p__Acidobacteriota; c__Acidobacteriae; o__Acidobacteriales; f__; g__; s__ |
| OTU_4690 | k__Bacteria; p__Proteobacteria; c__Alphaproteobacteria; o__Acetobacterales; f__Acetobacteraceae; g__; s__ |
| OTU_4694 | k__Bacteria; p__Actinobacteriota; c__Thermoleophilia; o__Solirubrobacterales; f__Solirubrobacteraceae |
| OTU_4723 | k__Bacteria; p__Chloroflexi; c__P2-11E; o__P2-11E; f__P2-11E; g__P2-11E; s__ |
| OTU_4727 | k__Bacteria; p__Proteobacteria; c__Alphaproteobacteria; o__Acetobacterales; f__Acetobacteraceae; g__Acidiphilium |
| OTU_4731 | k__Bacteria; p__Proteobacteria; c__Alphaproteobacteria; o__Elsterales; f__; g__; s__ |
| OTU_4733 | k__Bacteria; p__Proteobacteria; c__Gammaproteobacteria; o__Burkholderiales; f__Nitrosomonadaceae; g__MND1; s__ |
| OTU_4735 | k__Bacteria; p__Proteobacteria; c__Alphaproteobacteria; o__Elsterales; f__; g__; s__ |
| OTU_4780 | k__Bacteria; p__Verrucomicrobiota; c__Verrucomicrobiae; o__Pedosphaerales; f__Pedosphaeraceae; g__Pedosphaeraceae; s__ |
| OTU_4799 | k__Bacteria; p__Actinobacteriota; c__Thermoleophilia; o__Solirubrobacterales; f__67-14; g__67-14; s__ |
| OTU_4825 | k__Bacteria; p__Proteobacteria; c__Alphaproteobacteria; o__Acetobacterales; f__Acetobacteraceae |
| OTU_4907 | k__Bacteria; p__Actinobacteriota; c__Actinobacteria; o__Frankiales |
| OTU_4921 | k__Bacteria; p__Acidobacteriota; c__Vicinamibacteria; o__Vicinamibacterales; f__; g__; s__ |
| OTU_4989 | k__Bacteria; p__Acidobacteriota; c__Acidobacteriae; o__Solibacterales; f__Solibacteraceae; g__Candidatus_Solibacter |
| OTU_5063 | k__Bacteria; p__Proteobacteria; c__Alphaproteobacteria; o__Rhodospirillales; f__; g__; s__ |
| OTU_5065 | k__Bacteria; p__Proteobacteria; c__Alphaproteobacteria; o__Rhizobiales; f__Beijerinckiaceae; g__Methylocapsa; s__ |
| OTU_5098 | k__Bacteria; p__Bacteroidota; c__Bacteroidia; o__Chitinophagales; f__Chitinophagaceae |
| OTU_5137 | k__Bacteria; p__Gemmatimonadota; c__Gemmatimonadetes; o__Gemmatimonadales; f__Gemmatimonadaceae; g__; s__ |
| OTU_5163 | k__Bacteria; p__Acidobacteriota; c__Vicinamibacteria; o__Vicinamibacterales; f__Vicinamibacteraceae; g__Vicinamibacteraceae; s__ |
| OTU_5170 | k__Bacteria; p__Acidobacteriota; c__Acidobacteriae; o__Bryobacterales; f__Bryobacteraceae; g__Bryobacter; s__ |
| OTU_5225 | k__Bacteria; p__Actinobacteriota; c__Actinobacteria; o__Micromonosporales; f__Micromonosporaceae; g__Actinoplanes |
| OTU_5254 | k__Bacteria; p__Bacteroidota; c__Bacteroidia; o__Chitinophagales; f__Chitinophagaceae |
| OTU_5256 | k__Bacteria; p__Acidobacteriota; c__Acidobacteriae; o__Acidobacteriales; f__; g__; s__ |
| OTU_5257 | k__Bacteria; p__Gemmatimonadota; c__Gemmatimonadetes; o__Gemmatimonadales; f__Gemmatimonadaceae; g__Gemmatimonas; s__ |
| OTU_5268 | k__Bacteria; p__Actinobacteriota; c__Actinobacteria; o__Frankiales; f__Geodermatophilaceae; g__Geodermatophilus; s__ |
| OTU_5285 | k__Bacteria; p__Nitrospirota; c__Nitrospiria; o__Nitrospirales; f__Nitrospiraceae; g__Nitrospira; s__ |
| OTU_5293 | k__Bacteria; p__Proteobacteria; c__Alphaproteobacteria; o__Rhizobiales; f__Xanthobacteraceae |
| OTU_5296 | k__Bacteria; p__Proteobacteria; c__Gammaproteobacteria; o__Gammaproteobacteria_Incertae_Sedis; f__Unknown_Family; g__Acidibacter; s__ |
| OTU_5322 | k__Bacteria; p__Proteobacteria; c__Alphaproteobacteria; o__Rhizobiales; f__Beijerinckiaceae; g__Roseiarcus; s__ |
| OTU_5326 | k__Bacteria; p__Verrucomicrobiota; c__Verrucomicrobiae; o__Pedosphaerales; f__Pedosphaeraceae; g__ADurb.Bin063-1; s__ |
| OTU_5339 | k__Bacteria; p__Proteobacteria; c__Alphaproteobacteria; o__Acetobacterales; f__Acetobacteraceae |
| OTU_5348 | k__Bacteria; p__Proteobacteria; c__Alphaproteobacteria; o__Rhizobiales; f__Xanthobacteraceae; g__; s__ |
| OTU_5360 | k__Bacteria; p__Acidobacteriota; c__Acidobacteriae; o__Acidobacteriales; f__Acidobacteriaceae_(Subgroup_1); g__Acidicapsa; s__Acidicapsa_sp. |
| OTU_5361 | k__Bacteria; p__Proteobacteria; c__Gammaproteobacteria; o__Burkholderiales; f__Nitrosomonadaceae; g__Ellin6067; s__ |
| OTU_5396 | k__Bacteria; p__Firmicutes; c__Bacilli; o__Paenibacillales; f__Paenibacillaceae; g__Paenibacillus; s__Paenibacillus_sinopodophylli |
| OTU_5439 | k__Bacteria; p__Actinobacteriota; c__Acidimicrobiia; o__IMCC26256; f__IMCC26256; g__IMCC26256; s__Aciditerrimonas_sp. |
| OTU_5446 | k__Bacteria; p__Proteobacteria; c__Alphaproteobacteria; o__Elsterales; f__; g__; s__ |
| OTU_5495 | k__Bacteria; p__Actinobacteriota; c__Thermoleophilia; o__Solirubrobacterales; f__67-14; g__67-14; s__ |
| OTU_5498 | k__Bacteria; p__WPS-2; c__WPS-2; o__WPS-2; f__WPS-2; g__WPS-2; s__ |
| OTU_5539 | k__Bacteria; p__Acidobacteriota; c__Acidobacteriae; o__Subgroup_2; f__Subgroup_2; g__Subgroup_2; s__ |
| OTU_5543 | k__Bacteria; p__Myxococcota; c__Polyangia; o__Polyangiales; f__Polyangiaceae; g__Minicystis; s__ |
| OTU_5560 | k__Bacteria; p__Actinobacteriota; c__Thermoleophilia; o__Solirubrobacterales; f__Solirubrobacteraceae |
| OTU_5570 | k__Bacteria; p__Acidobacteriota; c__Acidobacteriae; o__Acidobacteriales; f__Acidobacteriaceae_(Subgroup_1); g__Occallatibacter; s__ |
| OTU_5580 | k__Bacteria; p__Actinobacteriota; c__Actinobacteria; o__Frankiales; f__Frankiaceae; g__Jatrophihabitans; s__ |
| OTU_5597 | k__Bacteria; p__Actinobacteriota; c__Thermoleophilia; o__Gaiellales; f__; g__; s__ |
| OTU_5620 | k__Bacteria; p__Actinobacteriota; c__Acidimicrobiia; o__IMCC26256; f__IMCC26256; g__IMCC26256; s__ |
| OTU_5638 | k__Bacteria; p__Gemmatimonadota; c__Gemmatimonadetes; o__Gemmatimonadales; f__Gemmatimonadaceae; g__; s__ |
| OTU_5648 | k__Bacteria; p__Actinobacteriota; c__Actinobacteria |
| OTU_5657 | k__Bacteria; p__Actinobacteriota; c__Actinobacteria; o__Micrococcales; f__Microbacteriaceae; g__Microbacterium |
| OTU_5658 | k__Bacteria; p__Myxococcota; c__Polyangia; o__Polyangiales; f__BIrii41; g__BIrii41; s__ |
| OTU_5684 | k__Bacteria; p__Acidobacteriota; c__Acidobacteriae; o__Bryobacterales; f__Bryobacteraceae; g__Bryobacter; s__ |
| OTU_5699 | k__Bacteria; p__Chloroflexi; c__TK10; o__TK10; f__TK10; g__TK10; s__bacterium_Ellin6519 |
| OTU_5704 | k__Bacteria; p__Myxococcota; c__Myxococcia; o__Myxococcales; f__Anaeromyxobacteraceae; g__Anaeromyxobacter; s__ |
| OTU_5733 | k__Bacteria; p__Chloroflexi; c__KD4-96; o__KD4-96; f__KD4-96; g__KD4-96; s__ |
| OTU_5750 | k__Bacteria; p__Acidobacteriota; c__Acidobacteriae; o__Acidobacteriales; f__; g__; s__ |
| OTU_5754 | k__Bacteria; p__Gemmatimonadota; c__Gemmatimonadetes; o__Gemmatimonadales; f__Gemmatimonadaceae; g__; s__ |
| OTU_5864 | k__Bacteria; p__Acidobacteriota; c__Acidobacteriae; o__Solibacterales; f__Solibacteraceae; g__Candidatus_Solibacter; s__ |
| OTU_5889 | k__Bacteria; p__Proteobacteria; c__Alphaproteobacteria; o__Rhizobiales; f__Xanthobacteraceae; g__; s__ |
| OTU_5892 | k__Bacteria; p__Actinobacteriota; c__Thermoleophilia; o__Solirubrobacterales |
| OTU_5896 | k__Bacteria; p__Actinobacteriota; c__MB-A2-108; o__MB-A2-108; f__MB-A2-108; g__MB-A2-108; s__ |
| OTU_5967 | k__Bacteria; p__Actinobacteriota; c__Thermoleophilia; o__Gaiellales; f__Gaiellaceae; g__Gaiella; s__ |
| OTU_5974 | k__Bacteria; p__Acidobacteriota; c__Acidobacteriae; o__Acidobacteriales; f__; g__; s__ |
| OTU_5990 | k__Bacteria; p__Actinobacteriota; c__Thermoleophilia; o__Gaiellales; f__Gaiellaceae; g__Gaiella; s__ |
| OTU_6004 | k__Bacteria; p__Acidobacteriota; c__Acidobacteriae; o__Solibacterales; f__Solibacteraceae; g__Candidatus_Solibacter; s__ |
| OTU_6036 | k__Bacteria; p__Verrucomicrobiota; c__Verrucomicrobiae; o__Chthoniobacterales; f__Chthoniobacteraceae; g__Candidatus_Udaeobacter; s__ |
| OTU_6052 | k__Bacteria; p__Proteobacteria; c__Alphaproteobacteria; o__Elsterales; f__; g__; s__ |
| OTU_6053 | k__Bacteria; p__Gemmatimonadota; c__Gemmatimonadetes; o__Gemmatimonadales; f__Gemmatimonadaceae; g__; s__ |
| OTU_6071 | k__Bacteria; p__Proteobacteria; c__Gammaproteobacteria; o__Burkholderiales; f__Comamonadaceae; g__Piscinibacter; s__ |
| OTU_6093 | k__Bacteria; p__Proteobacteria; c__Gammaproteobacteria; o__Pseudomonadales; f__Pseudomonadaceae; g__Pseudomonas |
| OTU_6110 | k__Bacteria; p__Acidobacteriota; c__Acidobacteriae; o__Bryobacterales; f__Bryobacteraceae; g__Bryobacter; s__ |
| OTU_6153 | k__Bacteria; p__Proteobacteria; c__Gammaproteobacteria; o__Burkholderiales; f__Nitrosomonadaceae; g__MND1; s__ |
| OTU_6221 | k__Bacteria; p__Chloroflexi; c__Ktedonobacteria; o__Ktedonobacterales; f__JG30-KF-AS9; g__JG30-KF-AS9; s__ |
| OTU_6237 | k__Bacteria; p__Acidobacteriota; c__Acidobacteriae; o__Solibacterales; f__Solibacteraceae; g__Candidatus_Solibacter; s__ |
| OTU_6275 | k__Bacteria; p__Acidobacteriota; c__Acidobacteriae; o__Bryobacterales; f__Bryobacteraceae; g__Bryobacter; s__ |
| OTU_6322 | k__Bacteria; p__Proteobacteria; c__Gammaproteobacteria; o__Burkholderiales; f__Comamonadaceae |
| OTU_6368 | k__Bacteria; p__Proteobacteria; c__Alphaproteobacteria; o__Rhizobiales; f__Rhizobiales_Incertae_Sedis; g__Bauldia; s__ |
| OTU_6391 | k__Bacteria; p__Proteobacteria; c__Gammaproteobacteria; o__Burkholderiales; f__Nitrosomonadaceae; g__MND1; s__ |
| OTU_6423 | k__Bacteria; p__Myxococcota; c__Polyangia; o__Polyangiales; f__Polyangiaceae |
| OTU_6431 | k__Bacteria; p__Actinobacteriota; c__Actinobacteria; o__Frankiales; f__Frankiaceae; g__Jatrophihabitans; s__ |
| OTU_6452 | k__Bacteria; p__Proteobacteria; c__Alphaproteobacteria; o__Rhizobiales; f__Xanthobacteraceae |
| OTU_6525 | k__Bacteria; p__Actinobacteriota; c__MB-A2-108; o__MB-A2-108; f__MB-A2-108; g__MB-A2-108; s__ |
| OTU_6561 | k__Bacteria; p__Planctomycetota; c__Planctomycetes; o__Planctomycetales; f__; g__; s__ |
| OTU_6573 | k__Bacteria; p__Verrucomicrobiota; c__Verrucomicrobiae; o__Chthoniobacterales; f__Chthoniobacteraceae; g__Candidatus_Udaeobacter; s__ |
| OTU_6662 | k__Bacteria; p__Actinobacteriota; c__Thermoleophilia; o__Gaiellales; f__; g__; s__ |
| OTU_6663 | k__Bacteria; p__Actinobacteriota; c__Acidimicrobiia; o__IMCC26256; f__IMCC26256; g__IMCC26256; s__ |
| OTU_6718 | k__Bacteria; p__Acidobacteriota; c__Acidobacteriae; o__Solibacterales; f__Solibacteraceae; g__Candidatus_Solibacter; s__ |
| OTU_6744 | k__Bacteria; p__Acidobacteriota; c__Acidobacteriae; o__Subgroup_2; f__Subgroup_2; g__Subgroup_2; s__ |
| OTU_6757 | k__Bacteria; p__Myxococcota; c__Myxococcia; o__Myxococcales; f__Myxococcaceae |
| OTU_6781 | k__Bacteria; p__Proteobacteria; c__Alphaproteobacteria; o__Rhizobiales; f__Hyphomicrobiaceae; g__Hyphomicrobium |
| OTU_6786 | k__Bacteria; p__Actinobacteriota; c__Thermoleophilia; o__Solirubrobacterales; f__67-14; g__67-14; s__ |
| OTU_6804 | k__Bacteria; p__Firmicutes; c__Bacilli; o__Bacillales; f__Planococcaceae; g__Sporosarcina |
| OTU_6841 | k__Bacteria; p__Proteobacteria; c__Gammaproteobacteria; o__Burkholderiales; f__Nitrosomonadaceae; g__MND1; s__ |
| OTU_6863 | k__Bacteria; p__Actinobacteriota; c__Actinobacteria; o__Frankiales; f__Acidothermaceae; g__Acidothermus |
| OTU_6870 | k__Bacteria; p__Proteobacteria; c__Gammaproteobacteria; o__Burkholderiales; f__SC-I-84; g__SC-I-84; s__ |
| OTU_6871 | k__Bacteria; p__Myxococcota; c__bacteriap25; o__bacteriap25; f__bacteriap25; g__bacteriap25; s__ |
| OTU_6905 | k__Bacteria; p__Acidobacteriota; c__Acidobacteriae; o__Acidobacteriales; f__Acidobacteriaceae_(Subgroup_1); g__; s__ |
| OTU_6942 | k__Bacteria; p__Proteobacteria; c__Gammaproteobacteria; o__Gammaproteobacteria_Incertae_Sedis; f__Unknown_Family; g__Acidibacter; s__ |
| OTU_6943 | k__Bacteria; p__Myxococcota; c__Polyangia; o__Polyangiales; f__Polyangiaceae; g__Pajaroellobacter; s__ |
| OTU_6955 | k__Bacteria; p__Actinobacteriota; c__Actinobacteria; o__Frankiales; f__Acidothermaceae; g__Acidothermus; s__ |
| OTU_6996 | k__Bacteria; p__Actinobacteriota; c__MB-A2-108; o__MB-A2-108; f__MB-A2-108; g__MB-A2-108; s__ |
| OTU_7000 | k__Bacteria; p__Proteobacteria; c__Alphaproteobacteria; o__Elsterales; f__; g__; s__ |
| OTU_7011 | k__Bacteria; p__Proteobacteria; c__Alphaproteobacteria; o__; f__; g__ |
| OTU_7046 | k__Bacteria; p__Proteobacteria; c__Gammaproteobacteria; o__Burkholderiales; f__Nitrosomonadaceae; g__Ellin6067; s__ |
| OTU_7062 | k__Bacteria; p__Acidobacteriota; c__Acidobacteriae; o__Acidobacteriales; f__Acidobacteriaceae_(Subgroup_1); g__Occallatibacter |
| OTU_7084 | k__Bacteria; p__Actinobacteriota; c__Acidimicrobiia; o__IMCC26256; f__IMCC26256; g__IMCC26256; s__ |
| OTU_7221 | k__Bacteria; p__Acidobacteriota; c__Acidobacteriae; o__Acidobacteriales; f__Acidobacteriaceae_(Subgroup_1); g__Edaphobacter; s__ |
| OTU_7226 | k__Bacteria; p__Chloroflexi; c__TK10; o__TK10; f__TK10; g__TK10; s__bacterium_Ellin6519 |
| OTU_7232 | k__Bacteria; p__Proteobacteria; c__Alphaproteobacteria; o__Rhizobiales; f__Xanthobacteraceae |
| OTU_7233 | k__Bacteria; p__Acidobacteriota; c__Acidobacteriae; o__Acidobacteriales; f__; g__; s__ |
| OTU_7239 | k__Bacteria; p__Verrucomicrobiota; c__Verrucomicrobiae; o__Pedosphaerales; f__Pedosphaeraceae; g__; s__ |
| OTU_7240 | k__Bacteria; p__Actinobacteriota; c__Actinobacteria; o__Corynebacteriales; f__Nocardiaceae; g__Nocardia; s__Nocardia_vinacea |
| OTU_7250 | k__Bacteria; p__Acidobacteriota; c__Acidobacteriae; o__Acidobacteriales; f__; g__; s__ |
| OTU_7276 | k__Bacteria; p__Actinobacteriota; c__MB-A2-108; o__MB-A2-108; f__MB-A2-108; g__MB-A2-108; s__ |
| OTU_7420 | k__Bacteria; p__Chloroflexi; c__AD3; o__AD3; f__AD3; g__AD3; s__ |
