# Supplementary material for: Disclosing the native blueberry rhizosphere community in Portugal—an integrated metagenomic and isolation approach
Source: PeerJ. 2023 Jun 27;11:e15525. doi: 10.7717/peerj.15525 (PMC10312161; doi:10.7717/peerj.15525)
Supplement: Supplemental Information 3 [file peerj-11-15525-s003.docx]

**Table S3.** Descriptive list of the endophytic and epiphytic fungi isolated and respective BLAST results.

| Code | Type | Genus | Species | E-value | Query Cover | % Identity |
| --- | --- | --- | --- | --- | --- | --- |
| M1.1a2 | Epiphytic | *Trichoderma* | *Trichoderma* sp. | 0.0 | 100% | 100.00% |
| M1.1a4.1 | Epiphytic | *Trichoderma* | *Trichoderma crassum* | 0.0 | 100% | 100.00% |
| M1.1a4.2 | Epiphytic | *Trichoderma* | *Trichoderma* sp. | 0.0 | 100% | 100.00% |
| M1.1b1 | Endophytic | *Trichoderma* | *Trichoderma spirale* | 0.0 | 100% | 100.00% |
| M1.1b3 | Endophytic | *Mucor* | *Mucor moelleri* | 0.0 | 97% | 99.72% |
| M1.1Ea3 | Epiphytic | *Fusarium* | *Fusarium oxysporum* | 0.0 | 100% | 100.00% |
| M1.1Eb3 | Endophytic | *Trichoderma* | *Trichoderma* sp. | 0.0 | 100% | 100.00% |
| M1.2a1 | Epiphytic | *Trichoderma* | *Trichoderma spirale* | 9,00E-136 | 100% | 100.00% |
| M1.2a2 | Epiphytic | *Mucor* | *Mucor moelleri* | 0.0 | 97 | 99.72% |
| M1.2Ea2 | Epiphytic | *Trichoderma* | *Trichoderma* sp. | 0.0 | 100% | 100.00% |
| M1.2Eb3 | Endophytic | *Phomopsis* | *Phomopsis* sp. | 8,00E-116 | 100% | 99.57% |
| M2.1a3 | Epiphytic | *Cunninghamella* | *Cunninghamella elegans* | 0.0 | 100% | 100.00% |
| M2.1Eb1 | Endophytic | *Trichoderma* | *Trichoderma* sp. | 0.0 | 100% | 100.00% |
| M2.2a1 | Epiphytic | *Trichoderma* | *Trichoderma citrinoviride* | 0.0 | 100% | 100.00% |
| M2.2b | Endophytic | *Trichoderma* | *Trichoderma virens* | 8,00E-121 | 100% | 100.00% |
| M2.2Ea1 | Epiphytic | *Diaporthe* | *Diaporthe columnaris* | 0.0 | 100% | 100.00% |
| M2.2Eb2 | Endophytic | *Trametes* | *Trametes villosa* | 0.0 | 100% | 100.00% |
| M2.2Eb3 | Endophytic | *Diaporthe* | *Diaporthe* sp. | 0.0 | 100% | 100.00% |
| M3.2a1 | Epiphytic | *Trichoderma* | *Trichoderma* sp. | 0.0 | 100% | 100.00% |
| M3.2a2 | Epiphytic | *Trichoderma* | *Trichoderma atroviride* | 0.0 | 100% | 100.00% |
| M3.2b1 | Endophytic | *Trichoderma* | *Trichoderma koningii* | 0.0 | 100% | 100.00% |
| M3.2b2 | Endophytic | *Trichoderma* | *Trichoderma spirale* | 1,00E-160 | 100% | 100.00% |
| M3.2Ea1 | Epiphytic | *Macrophomina* | *Macrophomina phaseolina* | 0.0 | 100% | 100.00% |
| M3.2Ea3 | Epiphytic | *Fusarium* | *Fusarium oxysporum* | 0.0 | 100% | 100.00% |
| M3.2Eb | Endophytic | *Trichoderma* | *Trichoderma atroviride* | 0.0 | 100% | 100.00% |
| M4.1a | Epiphytic | *Trichoderma* | *Trichoderma hamatum* | 0.0 | 100% | 100.00% |
| M4.1Ea2 | Epiphytic | *Diaporthe* | *Diaporthe columnaris* | 0.0 | 100% | 100.00% |
| M4.1Eb1 | Endophytic | *Fusarium* | *Fusarium oxysporum* | 0.0 | 100% | 100.00% |
| M4.1Eb2 | Endophytic | *Phomopsis* | *Phomopsis* sp. | 0.0 | 100% | 100.00% |
| M4.2a1 | Epiphytic | *Trichoderma* | *Trichoderma sulphureum* | 0.0 | 100% | 100.00% |
| M4.2a2 | Epiphytic | *Trichoderma* | *Trichoderma* sp. | 1,00E-175 | 100% | 100.00% |
| M4.2Eb | Endophytic | *Trichoderma* | *Trichoderma hamatum* | 0.0 | 100% | 100.00% |
| M5.1a1 | Epiphytic | *Trichoderma* | *Trichoderma spirale* | 0.0 | 100% | 100.00% |
| M5.1b | Endophytic | *Trichoderma* | *Trichoderma* sp. | 0.0 | 100% | 100.00% |
| M5.2a3 | Epiphytic | *Trichoderma* | *Trichoderma crassum* | 0.0 | 100% | 100.00% |
| M5.2Ea | Epiphytic | *Phomopsis* | *Phomopsis columnaris* | 0.0 | 100% | 100.00% |
| M6.1a1 | Epiphytic | *Trichoderma* | *Trichoderma citrinoviride* | 7,00E-142 | 100% | 100.00% |
| M6.1Ea1 | Epiphytic | *Diaporthe* | *Diaporthe columnaris* | 0.0 | 100% | 100.00% |
| M6.1Eb | Endophytic | *Trichoderma* | *Trichoderma longibrachiatum* | 1,00E-123 | 100% | 100.00% |
| M6.2Ea | Epiphytic | *Oidiodendron* | *Oidiodendron maius* | 3,00E-166 | 100% | 100.00% |
| M6.2Eb | Endophytic | *Phomopsis* | *Phomopsis columnaris* | 0.0 | 100% | 100.00% |
| M7.1Ea2 | Epiphytic | *Phomopsis* | *Phomopsis columnaris* | 0.0 | 100% | 100.00% |
| M7.2Eb3 | Endophytic | *Rosellinia* | *Rosellinia necatrix* | 5,00E-179 | 100% | 100.00% |
| M8.1Ea2 | Epiphytic | *Phomopsis* | *Phomopsis columnaris* | 2,00E-177 | 100% | 100.00% |
| M8.2a1 | Epiphytic | *Trichoderma* | *Trichoderma* sp. | 0.0 | 100% | 100.00% |
| M8.2b2.1 | Endophytic | *Trichoderma* | *Trichoderma harzianum* | 0.0 | 100% | 100.00% |
| M8.2Ea | Epiphytic | *Fusarium* | *Fusarium oxysporum* | 0.0 | 100% | 100.00% |
| M8.2Ea3 | Epiphytic | *Penicillium* | *Penicillium paraherquei* | 4,00E-175 | 100% | 100.00% |
| M8.2Eb1 | Endophytic | *Fusarium* | *Fusarium* sp. | 0.0 | 100% | 100.00% |
| M8.2Eb2 | Endophytic | *Fusarium* | *Fusarium oxysporum* | 0.0 | 100% | 100.00% |
| M8.2Eb4 | Endophytic | *Fusarium* | *Fusarium diaminii* | 0.0 | 100% | 100.00% |
| M9.1a1 | Epiphytic | *Trichoderma* | *Trichoderma gamsii* | 4,00E-155 | 100% | 100.00% |
| M9.1a2.1 | Epiphytic | *Trichoderma* | *Trichoderma* sp. | 0.0 | 100% | 100.00% |
| M9.1a2.2 | Epiphytic | *Trichoderma* | *Trichoderma asperellum* | 0.0 | 100% | 100.00% |
| M9.1b2 | Endophytic | *Sclerotium* | *Sclerotium glucanicum* | 0.0 | 100% | 99.79% |
| M9.1b3 | Endophytic | *Trichoderma* | *Trichoderma hamatum* | 0.0 | 100% | 100.00% |
| M9.1b4 | Endophytic | *Trichoderma* | *Trichoderma* sp. | 0.0 | 100% | 100.00% |
| M9.1Ea3 | Epiphytic | *Fusarium* | *Fusarium oxysporum* | 0.0 | 100% | 100.00% |
| M9.1Eb | Endophytic | *Setophoma* | *Setophoma terrestris* | 0.0 | 100% | 100.00% |
| M9.2a2 | Epiphytic | *Mucor* | *Mucor moelleri* | 5,00E-76 | 97% | 99.72% |
| M9.2a4 | Epiphytic | *Fusarium* | *Fusarium oxysporum* | 0.0 | 100% | 100.00% |
| M9.2Ea3 | Epiphytic | *Phomopsis* | *Phomopsis* sp. | 3,00E-140 | 100% | 100.00% |
| M9.2Eb3 | Endophytic | *Penicillium* | *Penicillium adametzii* | 0.0 | 100% | 100.00% |
