# Supplementary material for: Disclosing the native blueberry rhizosphere community in Portugal—an integrated metagenomic and isolation approach
Source: PeerJ. 2023 Jun 27;11:e15525. doi: 10.7717/peerj.15525 (PMC10312161; doi:10.7717/peerj.15525)
Supplement: Supplemental Information 4 [file peerj-11-15525-s004.docx]

**Table S4.** Descriptive list of the endophytic and epiphytic bacteria isolated and respective BLAST results.

| Code | Genus | Species | E-value | Query Cover | % Identity |
| --- | --- | --- | --- | --- | --- |
| LB M1.1a4 | *Bacillus* | *Bacillus thurigiensis* | 4,00E-34 | 100% | 100.00% |
| LB M1.1a1 | *Priestia* | *Priestia megaterium* | 3,00E-57 | 100% | 100.00% |
| BHI M6.1a1 | *Bacillus* | *Bacillus* sp. | 0.0 | 100% | 100.00% |
| LB M8.2a2 | *Bacillus* | *Bacillus* sp. | 0.0 | 100% | 100.00% |
| BHI M6.1b2 | *Bacillus* | *Bacillus* sp. | 2,00E-131 | 100% | 100.00% |
| LB M4.1Eb2 | *Erwinia* | *Erwinia billingiae* | 0.0 | 100% | 100.00% |
| BHI M7.2Eb2 | *Bacillus* | *Bacillus* sp. | 0.0 | 100% | 100.00% |
| LB M1.1Eb3 | *Bacillus* | *Bacillus* sp. | 1,00E-154 | 100% | 100.00% |
| LB M2.2a3.2 | *Klebsiella* | *Klebsiella* sp. | 8,00E-58 | 100% | 100.00% |
| LB M1.1a2.1 | *Bacillus* | *Bacillus* sp. | 3,00E-150 | 100% | 100.00% |
| BHI M3.1a1 | *Bacillus* | *Bacillus* sp. | 0.0 | 99% | 99.81% |
| LB M5.1b1 | *Priestia* | *Priestia* sp. | 0.0 | 100% | 100.00% |
| LB M4.1Eb3 | *Pantoea* | *Pantoea* sp. | 0.0 | 100% | 99.88% |
| BHI M6.2a6.2 | *Lysinibacillus* | *Lysinibacillus* sp. | 0.0 | 100% | 100.00% |
| BHI M5.2b2.3 | *Pseudomonas* | *Pseudomonas* sp. | 0.0 | 100% | 100.00% |
| LB M8.2Eb1 | *Lysinibacillus* | *Lysinibacillus* sp. | 0.0 | 100% | 100.00% |
| LB M1.1b1 | *Priestia* | *Priestia* sp. | 0.0 | 100% | 100.00% |
| BHI M5.2a4 | *Priestia* | *Priestia* sp. | 0.0 | 100% | 99.21% |
| BHI M9.1Ea4 | *Leifsonia* | *Leifsonia* sp. | 0.0 | 100% | 100.00% |
| BHI M4.1Ea2.1.3 | *Pantoea* | *Pantoea* sp. | 0.0 | 100% | 99.89% |
| BHI M8.2a4 | *Citrobacter* | *Citrobacter* sp. | 0.0 | 100% | 100.00% |
| BHI M7.1b1 | *Bacillus* | *Bacillus* sp. | 0.0 | 100% | 100.00% |
| LB M9.1Ea3 | *Paenibacillus* | *Paenibacillus* sp. | 0.0 | 100% | 100.00% |
| BHI M1.2b1.1 | *Enterobacter* | *Enterobacter* sp. | 1,00E-114 | 100% | 100.00% |
| LB M1.1b2.1 | *Priestia* | *Priestia* sp. | 0.0 | 100% | 100.00% |
| LB M3.1b2 | *Pseudomonas* | *Pseudomonas* sp. | 0.0 | 100% | 100.00% |
| BHI M5.2b2.1 | *Lysinibacillus* | *Lysinibacillus* sp. | 2,00E-173 | 100% | 99.13% |
| BHI M8.1a1 | *Pseudomonas* | *Pseudomonas* sp. | 0.0 | 100% | 100.00% |
| BHI M7.2Eb1.1 | *Lysinibacillus* | *Lysinibacillus* sp. | 0.0 | 100% | 100.00% |
| BHI M9.1Eb1 | *Paenibacillus* | *Paenibacillus* sp. | 0.0 | 100% | 99.38% |
| BHI M2.1b4 | *Lysinibacillus* | *Lysinibacillus* sp. | 0.0 | 100% | 100.00% |
| BHI M6.2Eb2 | *Priestia* | *Priestia* sp. | 0.0 | 100% | 100.00% |
| LB M1.1a2.2 | *Staphylococcus* | *Staphylococcus* sp. | 0.0 | 100% | 100.00% |
| LB M8.2Ea5 | *Bacillus* | *Bacillus* sp. | 0.0 | 100% | 100.00% |
| BHI M9.1a2.1 | *Pseudomonas* | *Pseudomonas* sp. | 0.0 | 100% | 100.00% |
| LB M5.1a1 | *Bacillus* | *Bacillus* sp. | 8,00E-126 | 100% | 100.00% |
| BHI M5.2Eb2 | *Micrococcus* | *Micrococcus* sp. | 0.0 | 100% | 100.00% |
| BHI M6.1a2.3 | *Micrococcus* | *Micrococcus* sp. | 0.0 | 100% | 99.76% |
| LB M7.1a1 | *Stenotrophomonas* | *Stenotrophomonas* sp. | 0.0 | 100% | 100.00% |
| LB M8.1Eb1 | *Lysinibacillus* | *Lysinibacillus* sp. | 0.0 | 100% | 100.00% |
| BHI M2.2Ea2 | *Bacillus* | *Bacillus* sp. | 0.0 | 100% | 100.00% |
| LB M2.1a3.2 | *Leclercia* | *Leclercia* sp. | 0.0 | 100% | 98.34% |
| LB M3.2 b5 | *Pantoea* | *Pantoea* sp. | 1E-137 | 99% | 85.99% |
| LB M1.1a3 | *Bacillus* | *Bacillus* sp. | 0.0 | 100% | 100.00% |
| LB M2.2b3 | *Bacillus* | *Bacillus* sp. | 0.0 | 100% | 99.86% |
| LB M1.1Ea3 | *Bacillus* | *Bacillus* sp. | 0.0 | 100% | 100.00% |
| LB M7.1a2 | *Bacillus* | *Bacillus* sp. | 0.0 | 100% | 100.00% |
| BHI M9.1Ea1 | *Lysinibacillus* | *Lysinibacillus* sp. | 0.0 | 100% | 100.00% |
| BHI M3.1a2 | *Acinetobacter* | *Acinetobacter* sp. | 5E-174 | 100% | 100.00% |
| LB M2.2Eb2.2 | *Priestia* | *Priestia* sp. | 0.0 | 100% | 100.00% |
| LB M4.1a3 | *Pseudomonas* | *Pseudomonas* sp. | 2E-122 | 100% | 99.20% |
| LB M1.2b3 | *Lysinibacillus* | *Lysinibacillus* sp. | 0.0 | 95% | 95.54% |
| LB M7.1b1 | *Priestia* | *Priestia* sp. | 0.0 | 100% | 100.00% |
| LB M3.1b5 | *Bacillaceae* | *-* | 0.0 | 100% | 100.00% |
| BHI M5.2a3.3 | *Chryseobacterium* | *Chryseobacterium* sp. | 0.0 | 100% | 100.00% |
| BHI M1.2b1.2 | *Priestia* | *Priestia* sp. | 0.0 | 100% | 100.00% |
| LB M4.2b4.1 | *Priestia* | *Priestia* sp. | 0.0 | 99% | 100.00% |
| LB M9.1b1 | *Pseudomonas* | *Pseudomonas* sp. | 0.0 | 100% | 100.00% |
| LB M2.2a1 | *Lelliottia* | *Lelliottia amnigena* | 0.0 | 100% | 100.00% |
