# Supplementary figures and images for: Disclosing the native blueberry rhizosphere community in Portugal—an integrated metagenomic and isolation approach
Source: PeerJ. 2023 Jun 27;11:e15525. doi: 10.7717/peerj.15525 (PMC10312161; doi:10.7717/peerj.15525)

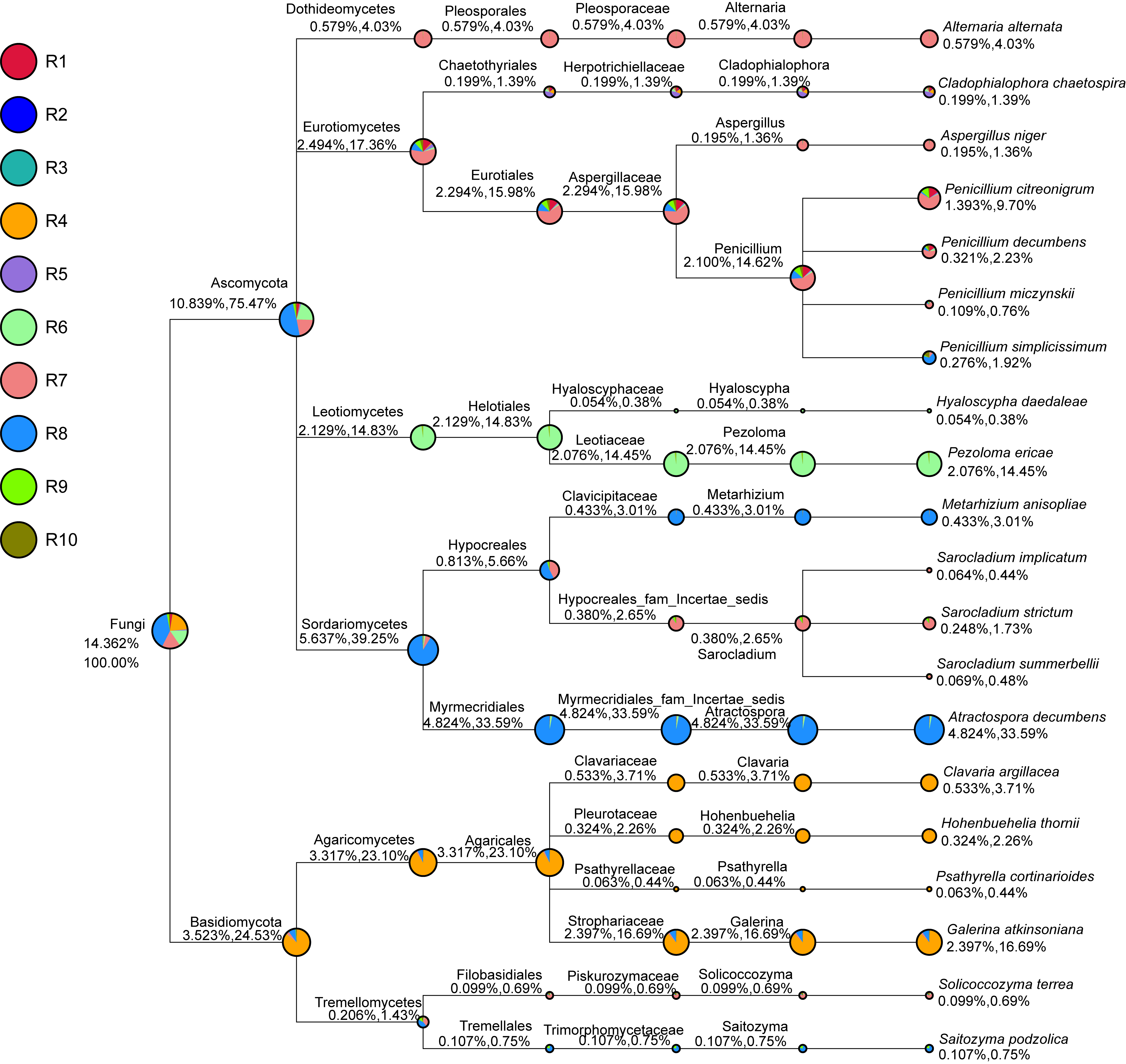

Supplement: Supplemental Information 5 [file peerj-11-15525-s005.png]

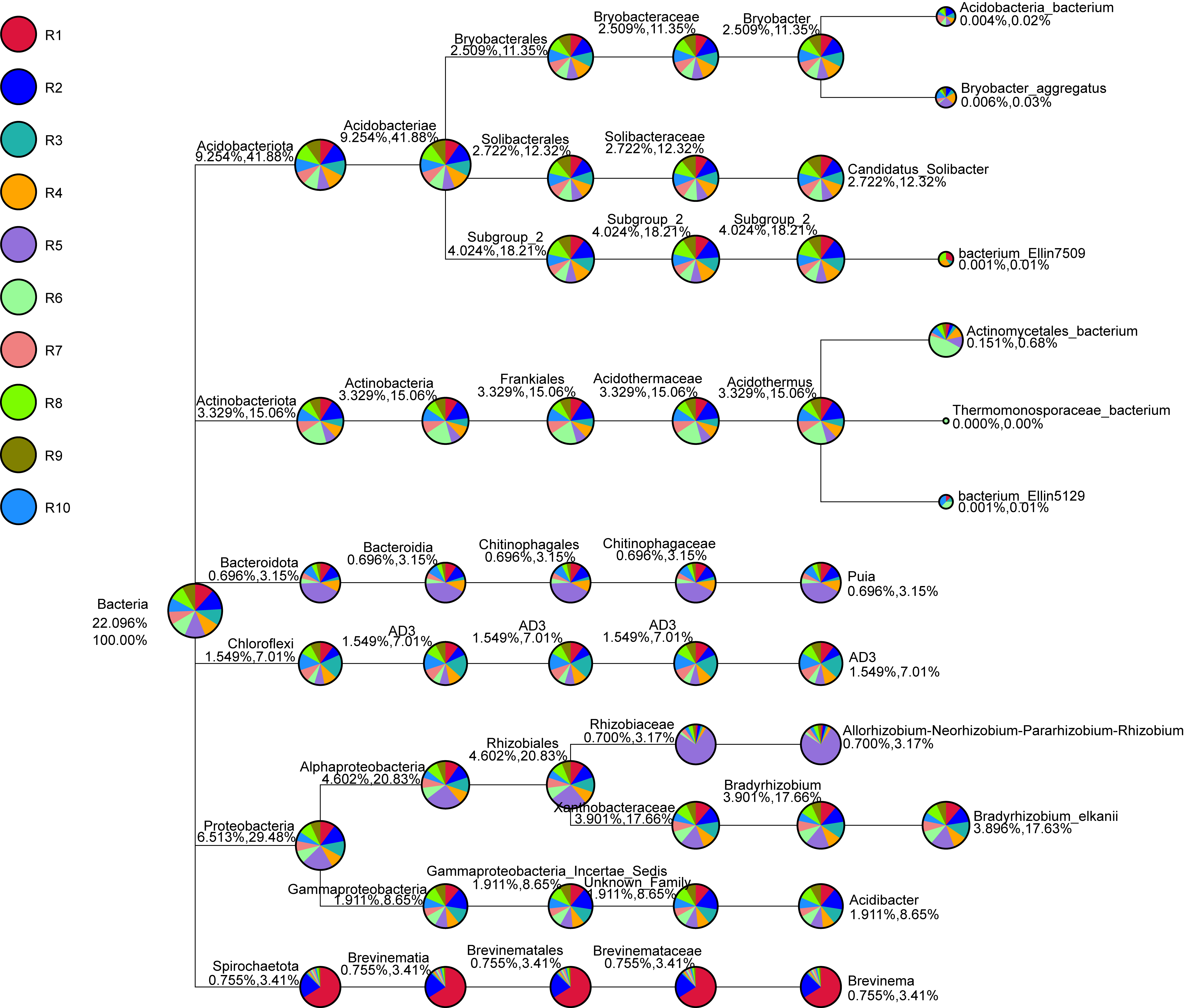

Supplement: Supplemental Information 6 [file peerj-11-15525-s006.png]
